# A multimodal sleep foundation model for disease prediction

**DOI:** 10.1038/s41591-025-04133-4

**Published:** 2026-01-06

**Authors:** Rahul Thapa, Magnus Ruud Kjaer, Bryan He, Ian Covert, Hyatt Moore IV, Umaer Hanif, Gauri Ganjoo, M. Brandon Westover, Poul Jennum, Andreas Brink-Kjaer, Emmanuel Mignot, James Zou

**Affiliations:** 1 Department of Biomedical Data Science, Stanford University, Stanford, CA USA; 2 Department of Computer Science, Stanford University, Stanford, CA USA; 3 Department of Psychiatry and Behavioral Sciences, Stanford University, Stanford, CA USA; 4 Department of Health Technology, Technical University of Denmark, Kongens Lyngby, Denmark; 5 Department of Clinical Neurophysiology, Danish Center for Sleep Medicine, Rigshospitalet, Glostrup, Denmark; 6 Department of Systems Engineering, Naval Postgraduate School, Monterey, CA USA; 7 Data Science, BioSerenity, Paris, France; 8 Department of Neurology, Beth Israel Deaconess Medical Center, Harvard Medical School, Boston, MA USA; 9 Department of Clinical Medicine, University of Copenhagen, Copenhagen, Denmark

**Keywords:** Diseases, Biomedical engineering

## Abstract

Sleep is a fundamental biological process with broad implications for physical and mental health, yet its complex relationship with disease remains poorly understood. Polysomnography (PSG)—the gold standard for sleep analysis—captures rich physiological signals but is underutilized due to challenges in standardization, generalizability and multimodal integration. To address these challenges, we developed SleepFM, a multimodal sleep foundation model trained with a new contrastive learning approach that accommodates multiple PSG configurations. Trained on a curated dataset of over 585,000 hours of PSG recordings from approximately 65,000 participants across several cohorts, SleepFM produces latent sleep representations that capture the physiological and temporal structure of sleep and enable accurate prediction of future disease risk. From one night of sleep, SleepFM accurately predicts 130 conditions with a C-Index of at least 0.75 (Bonferroni-corrected *P* < 0.01), including all-cause mortality (C-Index, 0.84), dementia (0.85), myocardial infarction (0.81), heart failure (0.80), chronic kidney disease (0.79), stroke (0.78) and atrial fibrillation (0.78). Moreover, the model demonstrates strong transfer learning performance on a dataset from the Sleep Heart Health Study—a dataset that was excluded from pretraining—and performs competitively with specialized sleep-staging models such as U-Sleep and YASA on common sleep analysis tasks, achieving mean *F*_1_ scores of 0.70–0.78 for sleep staging and accuracies of 0.69 and 0.87 for classifying sleep apnea severity and presence. This work shows that foundation models can learn the language of sleep from multimodal sleep recordings, enabling scalable, label-efficient analysis and disease prediction.

## Abstract (web summary)

A deep learning-based model, developed using the rich, multimodal data available from polysomnography-derived sleep recordings, performs well on common sleep analysis tasks and predicts future disease risk across a range of diseases.

## Main

Sleep is a complex process characterized by intricate interactions across physiological systems, including brain, heart, respiratory and muscle activity^1^. PSG—the gold standard for sleep evaluation—captures these interactions through recordings of several modalities, including brain activity signals (BAS, including electroencephalogram (EEG) and electrooculogram (EOG)), electrocardiography (ECG), electromyography (EMG) and respiratory signals^2^.

Sleep disorders affect millions of people and are increasingly recognized as indicators of, and contributors to, various health conditions^3^. Sleep disturbances often precede the clinical onset of numerous conditions, such as psychiatric disorders^4^, neurodegenerative diseases^5^ and cardiovascular disorders^6^. These associations highlight the important role sleep plays in maintaining overall health and underscores its predictive potential across a wide spectrum of diseases. However, most existing studies have focused on identifying links between sleep and specific diseases using isolated metrics or manual annotations, leaving much of the complexity of sleep physiology, as captured in PSG, underutilized.

Recent advances in deep learning have enabled the use of PSG’s multimodal data for tasks ranging from sleep staging and apnea detection to predicting conditions such as atrial fibrillation, biological aging and narcolepsy^3,7–10^. Despite this progress, current approaches face key limitations: they focus on individual outcomes, depend on supervised learning with expert-labeled data and are trained on relatively small datasets (2,500–15,913 recordings)^3,7,9–11^. Manual annotations are time consuming and prone to inter-rater variability, making scaling difficult. Moreover, existing models lack flexibility across recording environments, generalize poorly across cohorts and often fail to exploit the richness of multimodal sleep signals. There remains a need for robust, generalizable architectures and systematic evaluation of sleep’s predictive value across a broad range of health conditions.

Foundation models have emerged as a transformative approach in machine learning, enabling robust representation learning from large-scale, unlabeled data^12^. By leveraging self-supervised learning, these models can be fine-tuned efficiently for diverse applications. In biomedicine, foundation models have demonstrated remarkable capabilities in analyzing complex, heterogeneous datasets, driving advances in disease prediction, patient stratification and therapeutic discovery^13,14^. Their ability to extract meaningful patterns from large-scale data has addressed many challenges associated with the diverse and high-dimensional nature of clinical datasets.

Despite these successes, their application to sleep remains limited. Sleep data, particularly from PSG, presents unique challenges due to its complexity and variability, including differences in the number and types of recording channel across clinical cohorts. Most sleep studies have focused narrowly on sleep-specific outcomes, constraining the broader potential of foundation models for disease prediction. In preliminary work, we explored self-supervised learning on PSG data in a smaller cohort of participants^11^. Although this effort highlighted the potential of foundation models for analyzing sleep data, it targeted primarily sleep-specific outcomes and lacked the flexibility to accommodate the diverse configurations of PSG recordings. These limitations emphasize the need for models that can generalize across heterogeneous datasets and systematically uncover the role of sleep in predicting a wider range of diseases.

In this paper we present SleepFM, a foundation model trained on over 585,000 h of PSG data from 65,000+ participants. SleepFM captures the diverse information present in multimodal sleep recordings—integrating EEG, ECG, EMG and respiratory signals. Its channel-agnostic architecture enables joint learning across several modalities, producing representations that generalize across environments. We also introduce a new leave-one-out (LOO) contrastive learning (CL) (LOO-CL) algorithm that aligns information across modalities during pretraining while remaining resilient to missing or heterogeneous channels during inference. Our model uses 5–25 times more data than previously trained supervised sleep^3,7,9,10^ or biosignal models^15,16^.

Inspired by phenome-wide association studies (PheWAS)^17^, we examined whether sleep characteristics, as captured by SleepFM, can predict the onset of a wide range of diseases. Leveraging electronic health record (EHR) disease codes, we develop a framework to systematically explore predictive associations between multimodal sleep and diverse health conditions.

## Dataset and SleepFM architecture

We describe our dataset and training procedures in detail in Methods. Briefly, we used PSG data from four primary cohorts: Stanford Sleep Clinic (SSC)^11^, BioSerenity^18,19^, the Multi-Ethnic Study of Atherosclerosis (MESA)^20,21^ and the Outcomes of Sleep Disorders in Older Men (MrOS)^20,22^. SSC includes 35,052 studies from participants aged 1–100 years; BioSerenity adds 18,900 studies from people aged 7–90 years; MESA and MrOS contribute 2,237 and 3,930 PSGs, respectively, from older adults. Together, these cohorts span 65,000 participants and more than 585,000 h of sleep recordings. We further evaluated generalization using the Sleep Heart Health Study (SHHS)^20,23^—a multicenter dataset of 6,441 adults aged 40 years and older, held out from pretraining and used solely for transfer learning. Dataset distributions postfiltering are shown in Table 1. Demographics for SSC and BioSerenity appear in Extended Data Tables 1 and 2, whereas details for SHHS, MrOS and MESA are available in their respective publications.

**Table 1:** Distribution of PSG recordings across cohorts and data splits

Split	SSC	BioSerenity	MESA	MROS	SHHS	Total
Train	24,137	18,869	1,747	3,340	3,291	51,384
Validation	764	100	10	18	500	1,392
Test	5,019	–	150	286	2,000	7,455
Temporal test	5,132	–	–	–	–	5,132
Total	35,052	18,969	1,907	3,644	5,791	65,363

The model was first pretrained on SSC, BioSerenity, MESA and MROS data, following which these same recordings were used for task-specific fine-tuning. The SHHS dataset is reserved exclusively for evaluating transfer learning capabilities and was used only during fine-tuning not during pretraining. The temporal test set consists of SSC recordings from 2020 onwards, used to evaluate model robustness to temporal distribution shifts. Dashes (–) indicate that no data is available for that split.

Our preprocessing pipeline begins by resampling all signals to 128 Hz for consistency across cohorts. Signals are then segmented into 5-s windows, which serve as the model’s fundamental input tokens. The architecture includes one-dimensional (1D) convolutional layers for feature extraction, followed by channel-agnostic attention pooling to address variability in channel number and order across cohorts. A transformer block captures temporal dependencies over a 5-min context window. During pretraining, we use a multimodal CL objective to align representations across all modalities. The robustness of the model stems from its channel-agnostic design, enabling it to accommodate missing channels, varying channel counts and heterogeneous signal types.

For downstream tasks, we leverage the pretrained model’s embeddings through lightweight fine-tuning. The token embeddings from different modalities are pooled again and processed by a two-layer long short-term memory (LSTM) network before passing through task-specific output heads. For patient-level prediction tasks (for example, disease prediction), an additional temporal pooling layer before the output layer compresses all token embeddings into a single 128-dimensional embedding.

To evaluate model performance across tasks, we use appropriate task-specific metrics. For classification tasks such as sex classification, we report area under the receiver operating characteristic curve (AUROC) and area under the precision-recall curve (AUPRC); for sleep apnea classification we show confusion matrices and report accuracy; for age estimation, we use mean absolute error (MAE) and Pearson correlation. Sleep staging is evaluated using the *F*_1_ score, which is well suited for class-imbalanced settings. For disease prediction, we report AUROC and Harrell’s concordance index (C-Index)—a standard survival analysis metric that measures the proportion of correctly ranked risk pairs. All metrics range from 0 to 1, with higher values indicating better performance; 95% confidence intervals (CIs) are computed using bootstrapping.

## SleepFM supports standard sleep analysis tasks

After pretraining SleepFM, we assessed the general utility of its learned representations by fine-tuning on four common benchmark tasks: age estimation, sex classification, sleep stage classification and sleep apnea classification. Although these tasks are not the main focus of our work, they are useful validations showing that the model captures fundamental sleep patterns. For all tasks, we trained lightweight LSTM-based heads on top of the frozen multimodal embeddings derived from entire nights of PSG data.

**Extended Data Fig. 1:**
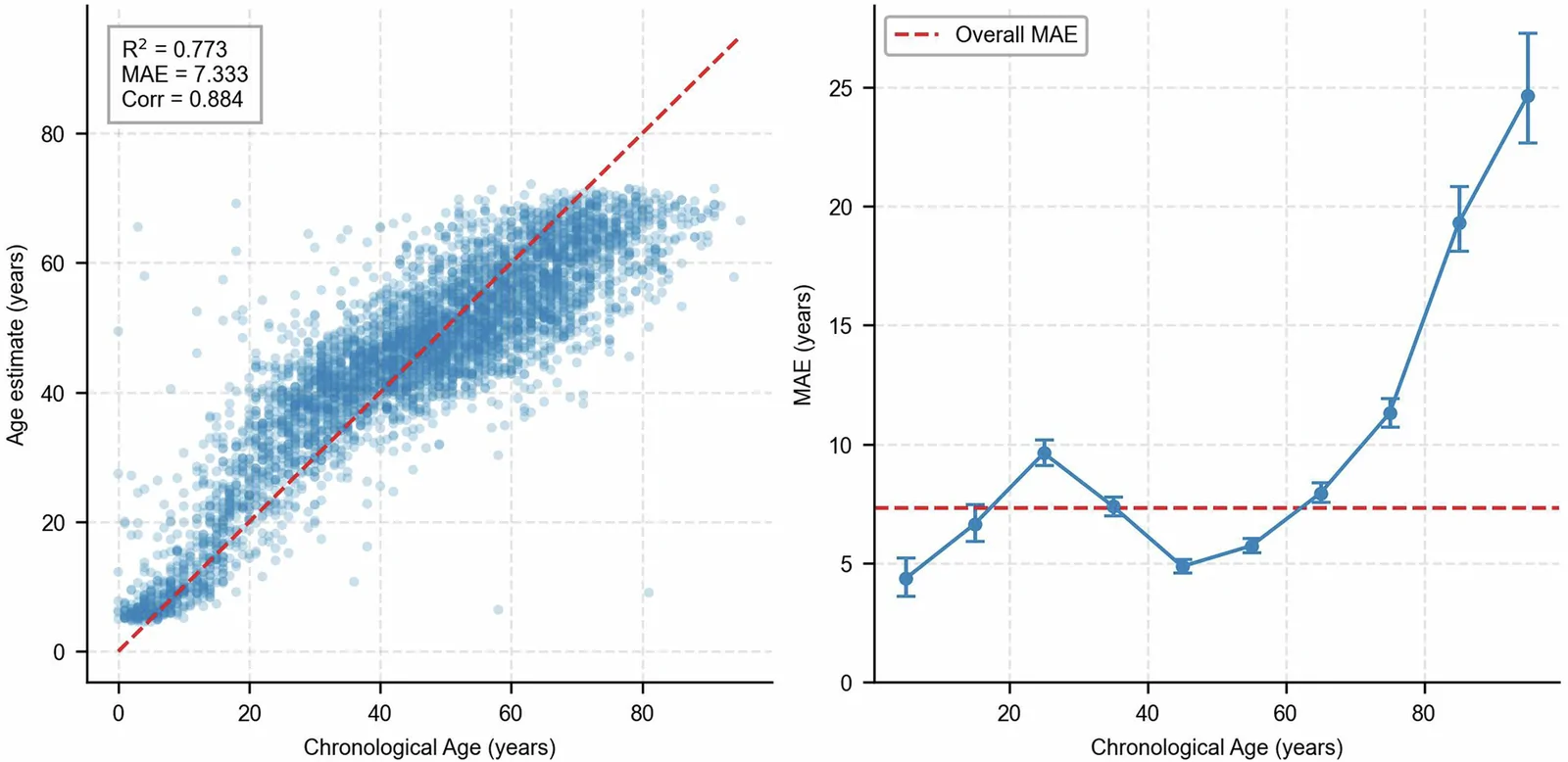
Age estimation performance on the Stanford cohort. Left: Scatterplot showing predicted versus chronological age across all patients (n=5,019), with the diagonal line representing perfect prediction. The coefficient of determination (R^2^), mean absolute error (MAE), and Pearson correlation coefficient (Corr) are shown in the top left corner. Right: Mean Absolute Error (MAE) across chronological age groups, with vertical error bars indicating the standard error of the mean (SEM) within each age bin. The horizontal dashed line represents the overall MAE. Our model achieves an MAE comparable to state-of-the-art models and demonstrates improved age estimation performance for younger age groups compared to older ones.

For age estimation, we assessed the ability of the model to predict chronological age. Overall performance is shown in Extended Data Fig. 1, with the model achieving a MAE of 7.33 years and a correlation coefficient of 0.88. Performance varied across age groups, with higher accuracy in pediatric and middle-aged participants and greater error in elderly adults, suggesting that age prediction is more challenging at the extremes of the age spectrum. Sex classification yielded an AUROC of 0.86 (0.85–0.87) and AUPRC of 0.90 (0.89–0.91). For sleep stage classification, we fine-tuned a LSTM-based classifier to distinguish Wake, Stage 1, Stage 2, Stage 3 and rapid eye movement (REM) using 5-s windows—a more granular resolution than the standard 30-s epochs, which has been shown to improve precision in certain conditions (for example, narcolepsy^10^). As shown in Supplementary Fig. 1, SleepFM performs well on Wake, Stage 2 and REM, with expected confusion in transitional stages like Stage 1—consistent with known human scoring variability. We report results across SSC, MESA, MrOS and SHHS, where SleepFM achieves competitive performance compared to U-Sleep^7^, YASA^24^, GSSC^25^ and STAGES^10^—state-of-the-art sleep staging models, as shown in Extended Data Tables 3 and 4. Furthermore, we compare SleepFM to three PhysioEx^26^ models on the public datasets DCSM^27^ and HMC^28^ in a fully external validation setting, achieving an *F*_1_ score of 0.68 on DCSM—outperforming all models—and 0.55 on HMC (Supplementary Table 1). Although the source alone has little impact, using several datasets for pretraining and fine-tuning improves generalization, boosting macro *F*_1_ by around 0.1 (Supplementary Tables 2, 3 and 4), consistent with previous work^26^.

**Fig. 1:**
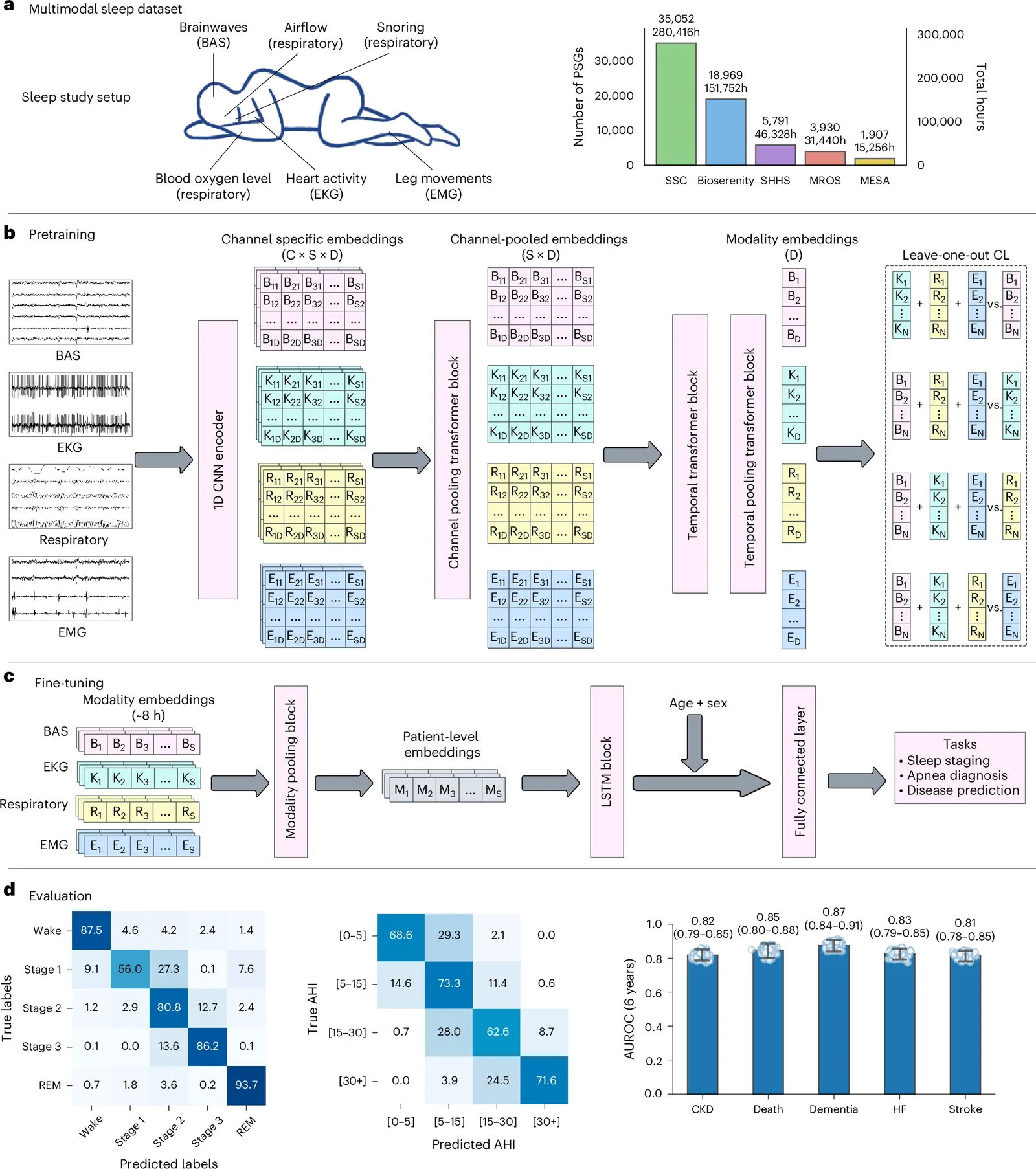
Overview of SleepFM framework. **a**, PSG setup and dataset statistics across several sleep centers. Bars show the number of independent PSG recordings (participants) per cohort and the corresponding total recording hours. **b**, Multimodal contrastive pretraining: raw signals from each modality are encoded by a CNN, channel embeddings are pooled within modality and a temporal transformer with temporal pooling yields sequence-level representations for LOO-CL. C: channels, S: sequence length, D: embedding dimension. **c**, Fine-tuning using frozen embeddings for downstream tasks (sleep staging, apnea detection, disease prediction). Eight hours of multimodal embeddings are aggregated to patient-level representations, concatenated with age and sex, and passed to an LSTM followed by a fully connected layer. **d**, Evaluation across representative tasks and clinical applications. Left and middle: confusion matrices for sleep staging (SHHS) and AHI categories (SSC) shown as row-normalized percentages. Right: disease prediction performance on the Stanford cohort (*n* = 5,019 participants). Box plots summarize 1,000 patient-level bootstrap resamples: faint dots (individual bootstrap draws), and vertical line with end caps (95% bootstrap percentile CI). Numeric labels are means. Number of positive samples for each disease: CKD (354), death (224), dementia (221), HF (283) and stroke (297).

For sleep apnea classification, we performed patient-level severity classification to distinguish between four commonly used severity groups on the basis of the apnea–hypopnea index (AHI): none (AHI < 5), mild (5 ≤ AHI < 15), moderate (15 ≤ AHI < 30) and severe (AHI ≥ 30). Across MESA, MrOs and SHHS, we observe competitive performance, with a severity classification accuracy of 0.69 and a presence classification accuracy (none/mild versus moderate/severe) of 0.87. The confusion matrix for apnea classification is shown in Fig. 1.

## SleepFM enables comprehensive disease prediction from sleep data

To enable disease prediction, we paired SSC data with EHRs, extracting all diagnostic codes (International Classification of Diseases, ninth revision (ICD-9) and International Classification of Diseases, tenth revision (ICD-10)) and their timestamps. These codes were mapped to phecodes—a hierarchical system of 1,868 disease categories designed for PheWAS^29^. The timestamp of each phecode was defined as the earliest among its corresponding ICD codes. Positive cases were defined as patients whose first phecode instance occurred more than 7 days after the sleep study, avoiding trivial associations. We excluded phecodes with prevalence below 1.5% to ensure statistical power, resulting in 1,041 phecodes for evaluation. For model fine-tuning, we used a multilabel extension of the Cox proportional hazards (CoxPH) loss, averaging independent losses computed for each label.

**Fig. 2:**
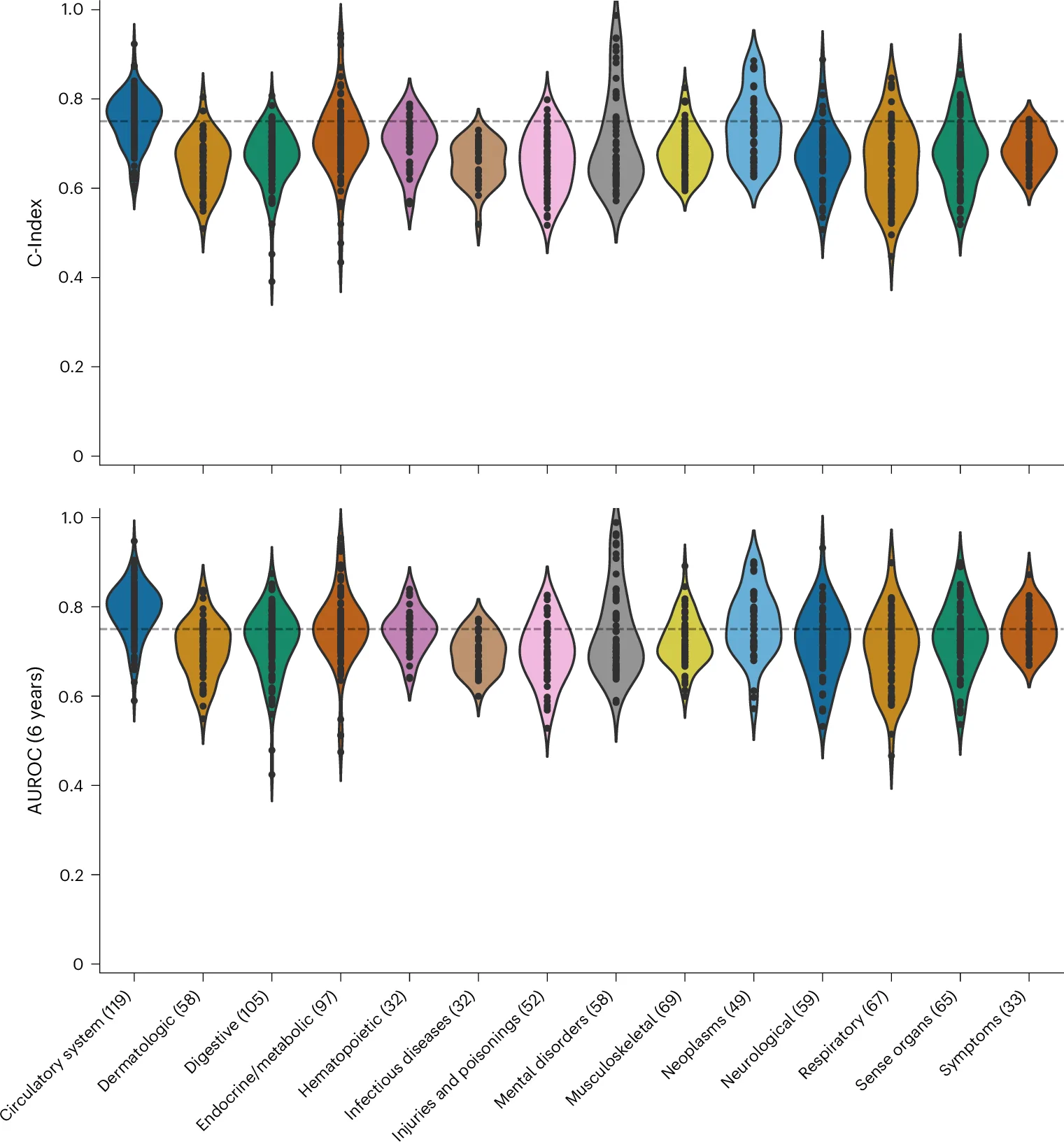
Performance of SleepFM on the held-out test set (*n* = 5,019) as stratified by disease category. Individual dots represent a disease within a category. The results are evaluated using two metrics: the C-Index, which measures the model’s ability to rank patient risk accurately, and the 6-year AUROC, which assesses the model’s discrimination performance by evaluating its ability to distinguish between patients who experience the event of interest and those who do not within a 6-year prediction window. For reference, the horizontal dashed line indicates a threshold of 0.75.

Figure 2 illustrates the performance of SleepFM across disease categories on the test set. Although performance varies across categories, SleepFM demonstrates strong results in several areas, including neoplasms, pregnancy complications, circulatory conditions and mental disorders. Overall, 130 future diseases achieved a C-Index and AUROC of at least 0.75 on held-out participants (Bonferroni-corrected *P* < 0.01), as summarized in Supplementary Table 5. AUROC was calculated using a 6-year horizon, meaning a condition is considered positive if the patient develops the disease within 6 years of their PSG study. The 6-year horizon for AUROC calculation was chosen to balance performance and account for both long-term and short-term conditions. Supplementary Fig. 2 shows AUROC values across 1–6 year horizons for several conditions.

The model showed high accuracy for mild cognitive impairment (AUROC 0.84 (0.80–0.880)), aligning with studies showing sleep disturbances as early markers of cognitive decline^30^. Strong performance was observed for Parkinson’s disease (0.93 (0.89–0.96)), where sleep disorders are increasingly recognized as potential early indicators^31^, and developmental delays and disorders (0.84 (0.79–0.87)). Among circulatory conditions, the model effectively predicted hypertensive heart disease (0.88 (0.85–0.91)) and intracranial hemorrhage (0.82 (0.73–0.90)), consistent with established links between sleep disorders and cardiovascular risk^32^. In the Neoplasm category, the model showed strong predictive performance for several cancers: prostate cancer (0.90 (0.87–0.93)), breast cancer (0.90 (0.86–0.93)) and melanomas of skin (0.83 (0.76–0.90)). These findings align with existing literature linking sleep patterns to cancer risk^33,34^.

**Extended Data Fig. 2:**
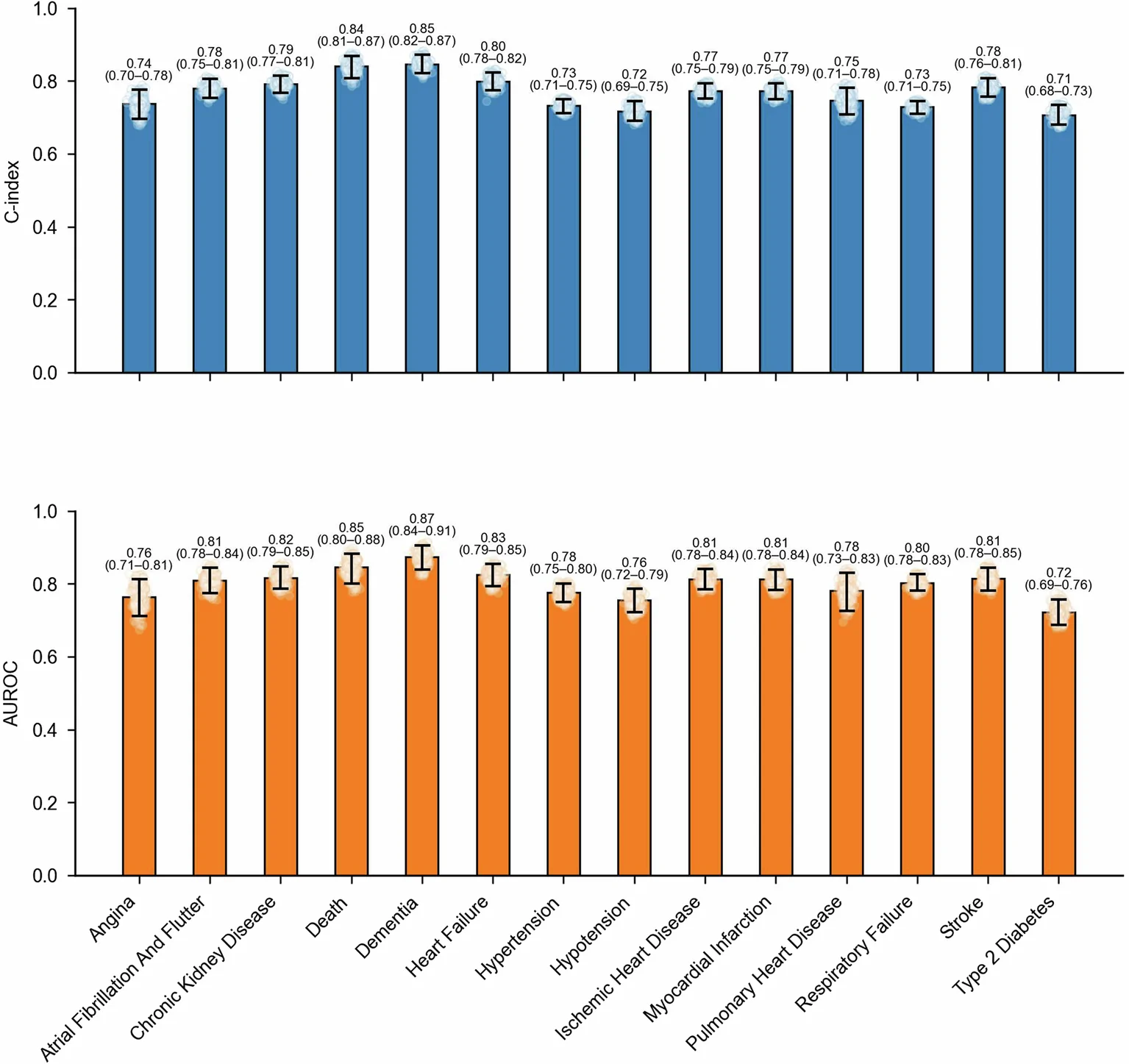
Performance across clinically relevant diseases evaluated on Stanford data (n=5019). Performance is evaluated using multiple metrics: C-Index and AUROC. The selected conditions include critical health outcomes such as death, heart failure, stroke, and dementia. Each panel uses violin/point plots derived from 1000 patient-level bootstrapping: the violin encodes the distribution of bootstrap estimates, faint points are individual bootstrap draws, the filled dot is the mean, and the vertical line with end caps marks the 95% bootstrap percentile CI. Numbers above violins report the mean. Metrics are C-index (top) and AUROC at 6 years (bottom).

Drawing on sleep expertise and previous literature, we identified 14 conditions with strong potential links to sleep patterns. Previous studies associate sleep regularity with mortality^35^, prolonged sleep with early neurodegeneration^36^ and sleep disturbances with dementia^37^, stroke^38^ and cardiovascular outcomes^9^. Related phecodes were grouped into unified disease categories in consultation with a medical doctor (Supplementary Table 6). Results for selected conditions—including death, stroke, heart failure (HF) and dementia—are shown in Extended Data Fig. 2. SleepFM demonstrates strong predictive performance, with particularly high accuracy for death (AUROC 0.84 (0.80–0.88)), HF (0.83 (0.79–0.86)), chronic kidney disease (CKD) (0.82 (0.79–0.85)), dementia (0.87 (0.84–0.91)) and stroke (0.81 (0.78–0.85)). All reported associations are statistically significant (*P* < 0.01, Bonferroni-corrected).

To better understand the physiological basis of disease prediction, we analyzed model performance stratified by both sleep stages and signal modalities. We found that although most sleep stages contribute similarly to disease prediction, certain stages such as Stage 1/2 and REM can offer slightly better predictive power for specific conditions, including cardiovascular and neurodegenerative diseases. Likewise, different signal modalities showed nuanced differences, with BAS signals better capturing mental and neurological conditions, respiratory signals more predictive of respiratory and metabolic disorders, and electrocardiogram (EKG) signals more informative for circulatory diseases. Although these differences align with known physiology, the overall predictive performance was highest when combining all modalities. Full results and condition-specific breakdowns are provided in Supplementary Figs. 3 and 4 and Supplementary Tables 7 and 8. Furthermore, we trained separate SleepFM models on each modality to directly assess modality-level importance. Performance comparisons stratified by disease category, presented in Supplementary Tables 9 and 10, further confirm that combining all modalities yields the optimal performance.

## SleepFM demonstrates robust generalization across time and cohorts

**Extended Data Fig. 3:**
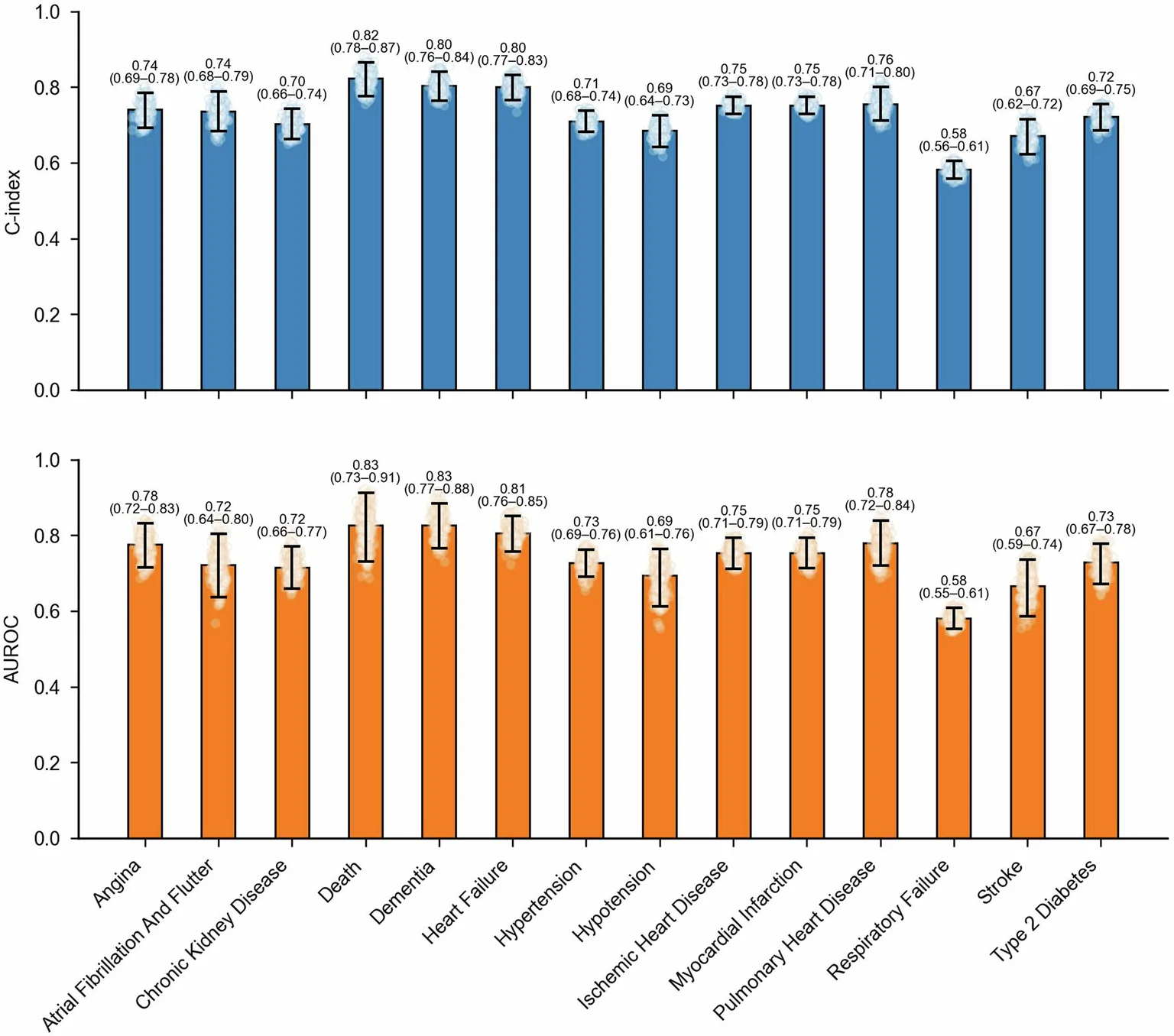
Performance of SleepFM for key clinical outcomes on the temporal test set. Metrics include C-Index and AUROC for critical conditions such as death, heart failure, chronic kidney disease, dementia, and stroke. Each panel uses violin/point plots derived from 1000 patient-level bootstrapping: the violin encodes the distribution of bootstrap estimates, faint points are individual bootstrap draws, the filled dot is the mean, and the vertical line with end caps marks the 95% bootstrap percentile CI. Numbers above violins report the mean. Metrics are C-index (top) and AUROC (bottom). All conditions are statistically significant with a p-value < 0.01 after Bonferroni correction.

We evaluate the generalization capabilities of SleepFM across temporal distribution shifts and external site validation. For temporal generalization, we test the model on a separate cohort comprising Stanford patients from 2020 onwards. All model pretraining and training was done on data from before 2020. Despite the limited follow-up period, SleepFM maintains strong predictive performance. Extended Data Fig. 3 shows results for our 14 selected conditions, with particularly robust and statistically significant performance (Bonferroni-corrected *P* < 0.01) for death (0.83 (0.73–0.91)), HF (0.80 (0.75–0.85)) and dementia (0.83 (0.76–0.89)). Comprehensive temporal-split performance across all disease phenotypes and categories is provided in Supplementary Figs. 5 and 6. Supplementary Fig. 7 further reports temporal-split performance comparisons with baseline models, stratified by disease category.

To assess cross-site generalization, we evaluate SleepFM’s transfer learning capabilities on SHHS—a dataset entirely excluded from the pretraining phase. We use the pretrained model to extract embeddings and then fine-tune it on a subset of SHHS. Specifically, the SHHS fine-tuning set includes 3,291 participants, and the test set includes 2,000 participants. Due to differences in task availability between SSC and SHHS, our evaluation focuses on six overlapping cardiovascular conditions. This setup mimics real-world deployment scenarios where foundation models must be adapted to new clinical sites with minimal supervision.

**Fig. 3:**
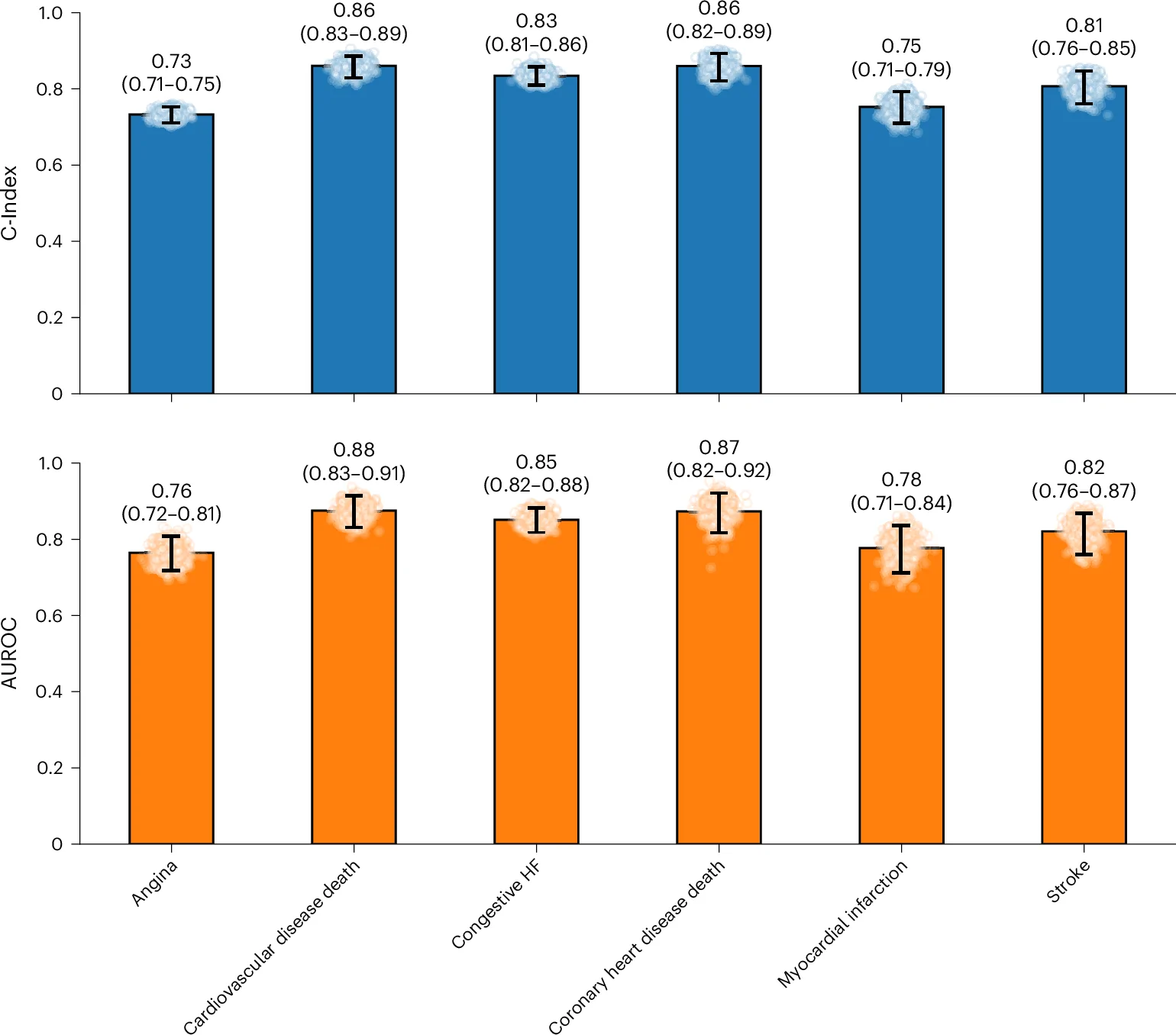
SleepFM prediction performance on the SHHS test set (*n* = 2,000 participants). Due to differences in available outcome data between SHHS and Stanford datasets, evaluation was limited to a subset of conditions. Results demonstrate transfer learning capabilities across these key clinical outcomes, including stroke, congestive HF and cardiovascular disease-related mortality. Each panel uses barplots derived from 1,000 patient-level bootstrapping: faint points are individual bootstrap draws, and the vertical line with end caps marks the 95% bootstrap percentile CI. Numbers above bars report the mean. Metrics are C-Index (top) and AUROC at 6 years (bottom). The number of positive samples for each outcome is as follows: angina (704), cardiovascular disease death (128), congestive HF (190), coronary heart disease death (80), myocardial infarction (103) and stroke (95). All conditions are statistically significant with a *P* value <0.01 after Bonferroni correction.

As shown in Fig. 3, SleepFM demonstrates strong transfer learning performance across key outcomes. For example, the model achieves statistically significant predictive accuracy (Bonferroni-corrected *P* < 0.01) for stroke (0.82 (0.76–0.87)), congestive HF (0.85 (0.82–0.88)) and mortality related to cardiovascular disease (0.88 (0.83–0.91)).

## SleepFM surpasses supervised baselines in disease prediction

**Fig. 4:**
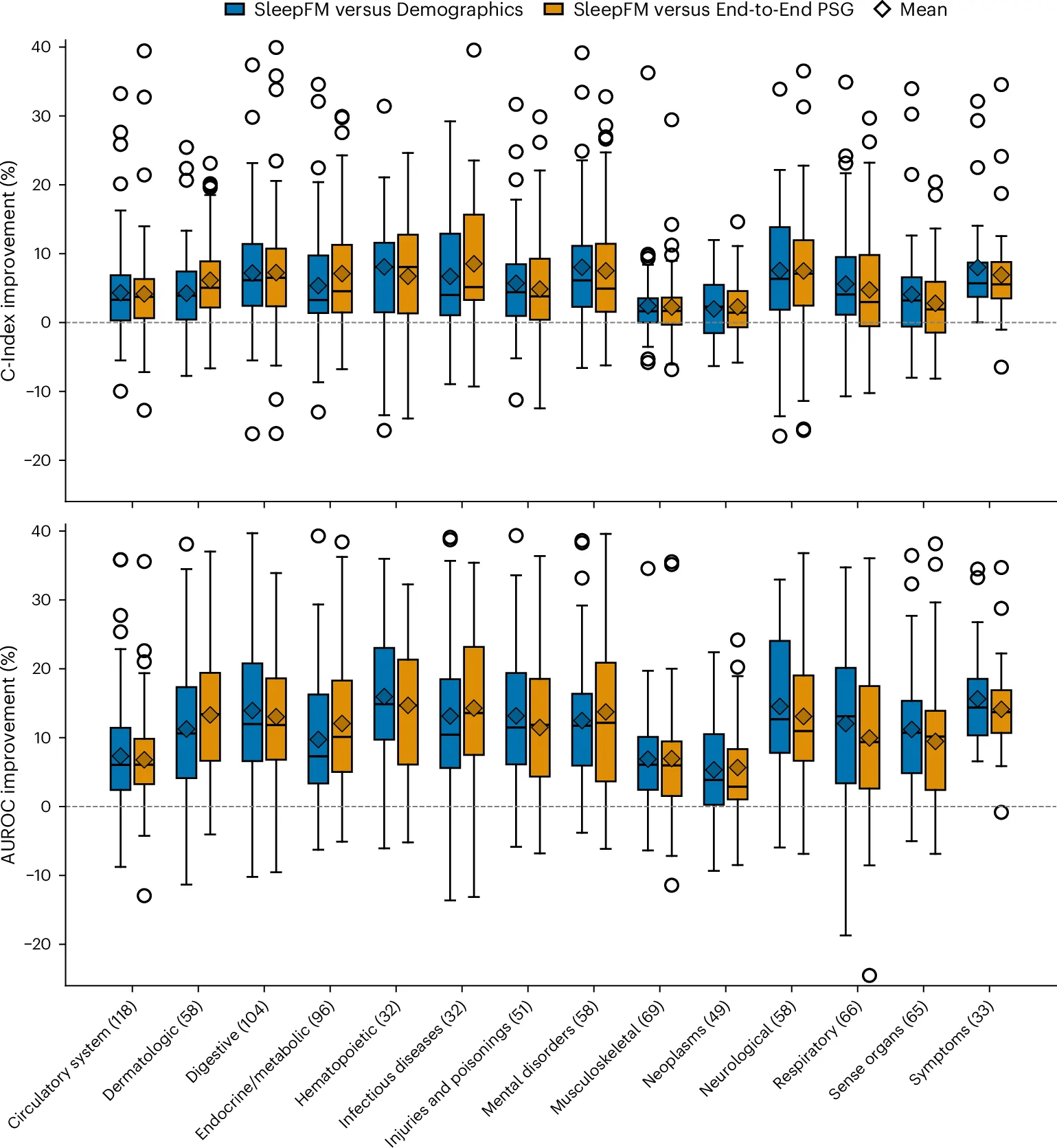
Performance improvements of SleepFM over baseline models across disease categories on Stanford test set (*n* = 5,019 participants). SleepFM and the End-to-End PSG model include age and sex demographic features, whereas the demographics-only model includes age, sex, BMI and race/ethnicity. Each box shows the distribution of disease-level percentage improvements of SleepFM relative to each baseline within the indicated disease category. Improvements are shown for both C-Index (top) and 6-year AUROC (bottom) metrics. Boxes represent the interquartile range (IQR), with whiskers extending to 1.5× IQR and outliers shown as points. Diamonds denote the mean improvement within each category. The horizontal dashed line at zero indicates no improvement.

We compare SleepFM against two supervised baselines: Demographics and End-to-End PSG. The demographics baseline is a multilayer perceptron (MLP) trained on structured clinical features (age, sex, race/ethnicity and body mass index (BMI)). This baseline includes more demographic variables than the SleepFM-based models, which only use age and sex. The End-to-End PSG model is trained directly on raw PSG data using the same architecture and parameter count as SleepFM, and it includes age and sex but does not use any pretraining. From Fig. 4, we observe that the percentage difference in AUROC between SleepFM and both baseline models ranges from 5% to 17%. The magnitude of improvement varies across disease categories; for example, gains are more pronounced in neurological and hematopoietic conditions, whereas in neoplasm-related conditions the improvements are comparatively modest. Supplementary Fig. 8 reports the overall test-set performance comparison between SleepFM and the baseline models across all disease phenotypes.

Next, we evaluated three different variants of SleepFM using identical training configurations, as shown in Table 2 and Extended Data Table 5. SleepFM-LSTM (without Demo) uses SleepFM embeddings with a two-layer LSTM fine-tuning head but no demographic features. SleepFM-Linear uses SleepFM embeddings with a simple linear prediction head and includes age and sex. Finally, SleepFM-LSTM, combines the pretrained SleepFM embeddings with a two-layer LSTM head and includes age and sex.

**Table 2:** Comparison of category-averaged AUROC across SleepFM variants and baselines

Category	Demo	E2E-PSG	SleepFM-1	SleepFM-2	SleepFM-3
Circulatory system	0.74_(0.73, 0.74)_	0.74_(0.73, 0.75)_	0.78_(0.77, 0.78)_	0.79_(0.78, 0.80)_	0.79_(0.78, 0.80)_
Dermatologic	0.64_(0.63, 0.65)_	0.63_(0.62, 0.64)_	0.68_(0.67, 0.69)_	0.71_(0.70, 0.72)_	0.70_(0.70, 0.71)_
Digestive	0.63_(0.62, 0.64)_	0.64_(0.63, 0.65)_	0.69_(0.69, 0.70)_	0.72_(0.71, 0.73)_	0.72_(0.71, 0.73)_
Endocrine/metabolic	0.68_(0.68, 0.69)_	0.67_(0.66, 0.68)_	0.74_(0.73, 0.75)_	0.75_(0.74, 0.76)_	0.75_(0.74, 0.76)_
Hematopoietic	0.64_(0.63, 0.66)_	0.66_(0.64, 0.67)_	0.73_(0.72, 0.75)_	0.75_(0.73, 0.76)_	0.74_(0.73, 0.76)_
Infectious diseases	0.62_(0.61, 0.64)_	0.62_(0.60, 0.63)_	0.67_(0.65, 0.69)_	0.70_(0.68, 0.71)_	0.70_(0.68, 0.71)_
Injuries and poisonings	0.62_(0.61, 0.63)_	0.63_(0.61, 0.64)_	0.68_(0.67, 0.69)_	0.70_(0.69, 0.71)_	0.70_(0.69, 0.71)_
Mental disorders	0.66_(0.65, 0.67)_	0.66_(0.66, 0.67)_	0.72_(0.71, 0.73)_	0.74_(0.73, 0.75)_	0.74_(0.74, 0.75)_
Musculoskeletal	0.68_(0.67, 0.68)_	0.68_(0.67, 0.69)_	0.70_(0.69, 0.71)_	0.72_(0.72, 0.73)_	0.72_(0.71, 0.73)_
Neoplasms	0.73_(0.71, 0.74)_	0.73_(0.71, 0.74)_	0.73_(0.72, 0.74)_	0.76_(0.75, 0.77)_	0.76_(0.75, 0.77)_
Neurological	0.62_(0.61, 0.63)_	0.63_(0.62, 0.64)_	0.70_(0.69, 0.71)_	0.72_(0.71, 0.73)_	0.72_(0.71, 0.73)_
Respiratory	0.63_(0.62, 0.64)_	0.64_(0.63, 0.65)_	0.69_(0.68, 0.70)_	0.69_(0.69, 0.70)_	0.70_(0.69, 0.71)_
Sense organs	0.66_(0.65, 0.67)_	0.67_(0.66, 0.68)_	0.71_(0.70, 0.72)_	0.73_(0.72, 0.74)_	0.73_(0.72, 0.74)_
Symptoms	0.65_(0.64, 0.66)_	0.66_(0.64, 0.67)_	0.72_(0.71, 0.73)_	0.75_(0.74, 0.76)_	0.75_(0.74, 0.76)_

Category-averaged 6-year AUROC (mean_(95% CI)_) comparing SleepFM variants with two baselines across disease categories on Stanford cohort (*n* = 5,019). The Demographics baseline (Demo) uses only structured clinical features (age, sex, BMI and race/ethnicity). The End-to-End PSG baseline (E2E-PSG) is trained directly on raw PSG signals with age and sex, without any pretraining. SleepFM-1 denotes SleepFM-LSTM (without Demo), using two LSTM layers in the fine-tuning prediction module and no demographic features. SleepFM-2 denotes SleepFM-Linear, a linear prediction module on SleepFM embeddings with age and sex. SleepFM-3 denotes SleepFM-LSTM, which uses two LSTM layers in the fine-tuning prediction module with age and sex. Values are averaged within each category across conditions. Uncertainty is estimated by nonparametric bootstrapping (*n* = 1,000 resamples): for each resample, conditions within a category are sampled with replacement and the category mean is computed; 95% CIs are the 2.5th–97.5th percentiles across resamples.

As seen in Table 2, the demographics-only baseline performs well, reflecting the fact that many diseases are associated strongly with age, sex, BMI and race/ethnicity. For example, in the Neoplasm category, older age is a strong predictor of cancer risk. Nevertheless, all SleepFM-based models, including the SleepFM-LSTM (without Demo) variant, consistently outperform the demographics and End-to-End PSG baselines across most disease categories. This demonstrates the benefit of using pretrained SleepFM embeddings for disease prediction. Furthermore, SleepFM-LSTM (without Demo) achieves over +5 AUROC points in 9 out of 14 conditions, whereas SleepFM-Linear and SleepFM-LSTM achieve over +5 AUROC points in 12 out of 14 conditions, compared to supervised demographics baseline. As seen from the 95% CI bars, these improvements are robust, with most differences being larger than the uncertainty intervals. Finally, SleepFM-Linear performs comparably to SleepFM-LSTM, suggesting that the strength of the model lies in the pretrained embeddings rather than the complexity of the downstream head. Percentage improvement comparisons across models are provided in Supplementary Fig. 9, and a scatterplot comparison of all disease phenotypes across different fine-tuning architectures on top of SleepFM is shown in Supplementary Fig. 10.

To further examine disease-specific performance, full results are provided in Supplementary Tables 11, 12 and 13, and clinician-selected conditions are presented in Supplementary Fig. 11. These comparisons show that SleepFM achieves substantial gains across several neurological, mental, circulatory, endocrine/metabolic and respiratory conditions. For neurological and mental disorders, SleepFM attains higher C-Index scores for senile dementia (0.99 (0.98–1.00) versus 0.87 (0.75–0.96)), myoneural disorders (0.81 (0.73–0.88) versus 0.42 (0.28–0.55)) and developmental delays (0.80 (0.77–0.84) versus 0.58 (0.51–0.64)). For circulatory diseases, SleepFM outperforms in atherosclerosis (0.92 (0.88–0.95) versus 0.74 (0.64–0.89)) and acute pulmonary heart disease (0.80 (0.75–0.85) versus 0.74 (0.68–0.80)). Improvements in endocrine/metabolic conditions include diabetes type 2 with circulatory complications (0.87 (0.83–0.91) versus 0.79 (0.74–0.85)) and diabetic retinopathy (0.81 (0.77–0.85) versus 0.75 (0.69–0.80)). For respiratory conditions, SleepFM achieves higher C-Index in respiratory insufficiency (0.79 (0.72–0.85)] versus 0.59 (0.51–0.67)) and failure (0.77 (0.73–0.80) versus 0.70 (0.65–0.74)). These findings highlight the versatility of SleepFM in predicting a broad range of diseases beyond what is captured by demographics alone.

Similarly, full comparisons with the End-to-End PSG model are provided in Supplementary Table 14. This comparison highlights the value of foundation model pretraining: although both models share similar architecture and input signals, SleepFM benefits from self-supervised pretraining, enabling more robust and informative representations. This advantage is reflected in consistent performance gains across neurological, circulatory, endocrine/metabolic and respiratory conditions. For neurological and mental disorders, SleepFM outperforms the end-to-end model in myoneural disorders (0.84 (0.75–0.91) versus 0.54 (0.40–0.69)), developmental delays (0.84 (0.79–0.87) versus 0.61 (0.52–0.69)) and speech/language disorders (0.83 (0.74–0.90) versus 0.71 (0.60–0.83)). For circulatory conditions, improvements are observed in atherosclerosis of native arteries of the extremities (0.95 (0.92–0.98) versus 0.65 (0.61–0.69)), atherosclerosis of the extremities (0.84 (0.75–0.90) versus 0.78 (0.71–0.85)) and acute pulmonary heart disease (0.84 (0.77–0.90) versus 0.76 (0.69–0.83)). In endocrine/metabolic disorders, SleepFM demonstrates stronger performance for predicting diabetes with circulatory complications (0.89 (0.85–0.93) versus 0.79 (0.70–0.87)), neurological manifestations (0.86 (0.81–0.90) versus 0.73 (0.67–0.78)) and diabetic retinopathy (0.84 (0.79, 0.89) versus 0.76 (0.69–0.82)). Respiratory conditions also benefit, with better performance in predicting respiratory insufficiency (0.82 (0.72–0.91) versus 0.64 (0.54–0.73)) and respiratory failure (0.76 (0.71–0.82) versus 0.68 (0.62–0.74)). In predicting all-cause mortality, SleepFM achieves a AUROC of 0.85 (0.80–0.89), outperforming both the Demographic baseline and End-to-End PSG model, which achieve AUROC of 0.78 (0.72–0.82).

**Extended Data Fig. 4:**
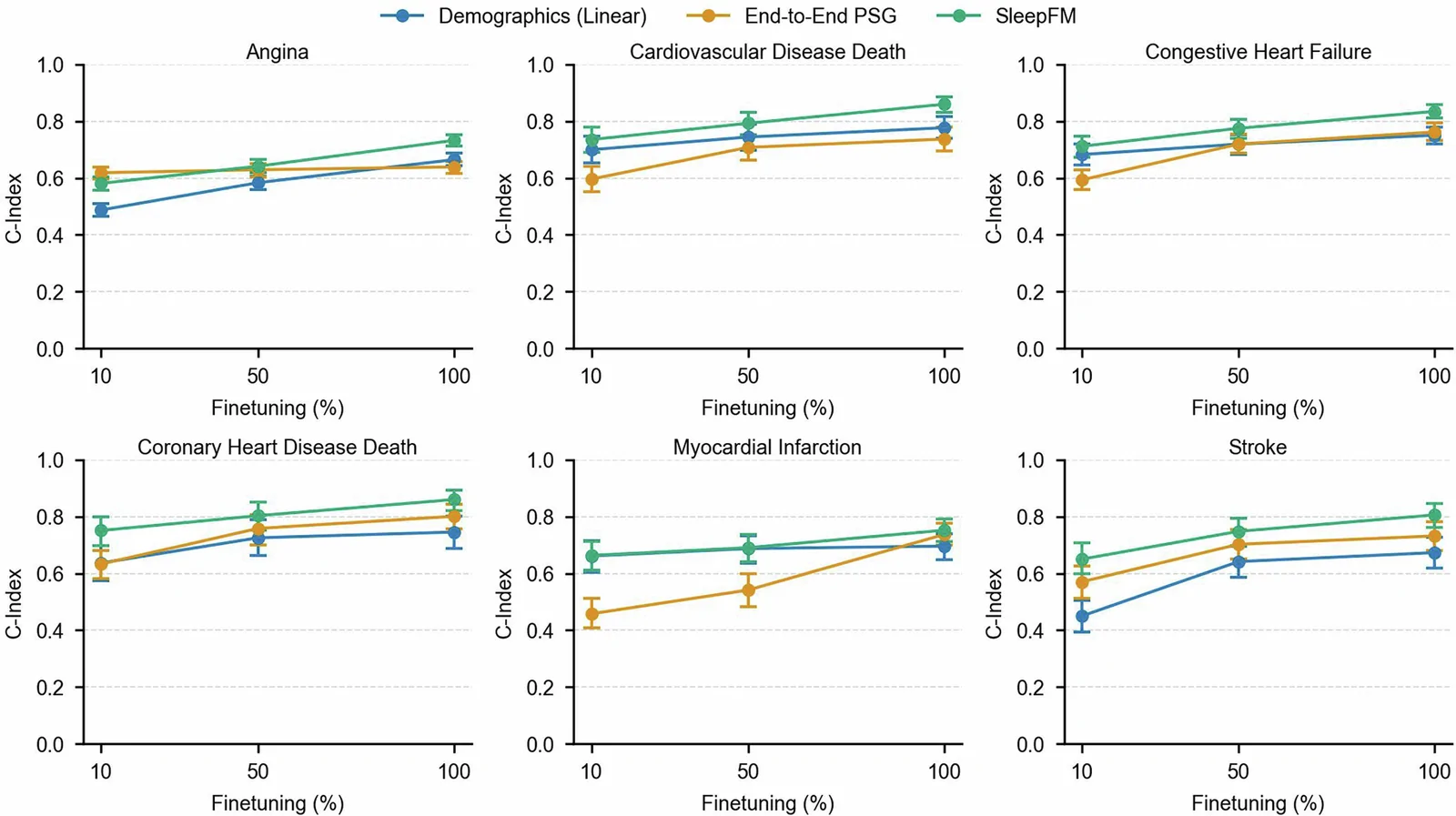
Scaling behavior of fine-tuning SleepFM on the SHHS dataset. Scaling behavior of fine-tuning SleepFM on the SHHS dataset (test size = 2,000 participants). We progressively increased the percentage of labeled SHHS data used during fine-tuning from 10% to 100%. The plots show C-Index performance across six cardiovascular outcomes, comparing SleepFM with Demographics and End-to-End PSG baselines. Error bars indicate 95% confidence intervals derived from 1,000 participant-level bootstrap resamples with replacement. Even with as little as 10% of training data (330 samples), SleepFM demonstrates strong predictive accuracy and consistent performance improvements as more labeled data becomes available. SleepFM outperforms both baseline models in most conditions, particularly when the dataset size is smaller, and its performance scaling is more stable across all outcomes.

**Extended Data Fig. 5:**
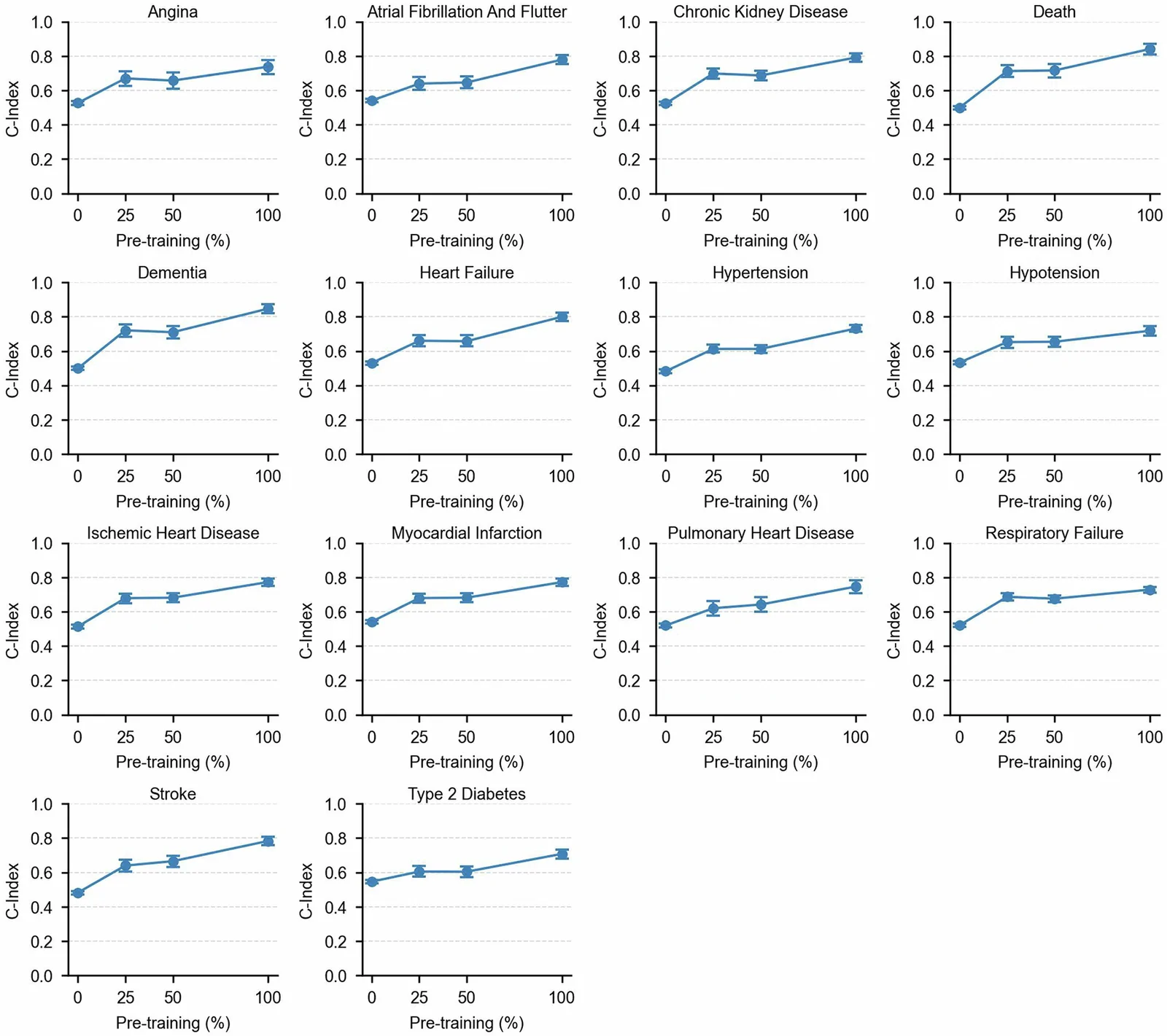
Impact of pretraining dataset size on downstream performance. Stanford cohort is used for this analysis. Each subplot shows C-Index performance for a specific disease as a function of the percentage of pretraining data used (0%, 25%, 50%, 100%). The downstream fine-tuning and test datasets are held constant. Error bars represent 95% confidence intervals estimated via 1,000 participant-level bootstrap resamples with replacement. The 100% mark corresponds to a full epoch of pretraining on the entire dataset (n=24,137). Intermediate checkpoints at 25% and 50% represent models saved partway through that epoch, while the 0% point denotes a model with no pretraining, resulting in near-random performance. Performance improves consistently with more pretraining data, highlighting the value of large-scale self-supervised pretraining across diverse phenotypes, including cardiovascular, metabolic, neurological, and respiratory conditions.

Finally, we compare fine-tuning scalability by evaluating SleepFM alongside two baseline models as we increase the amount of fine-tuning data and measure performance on the same test set. These results are shown in Extended Data Fig. 4 for SHHS and Extended Data Fig. 5 and Supplementary Fig. 12 for SSC. In both plots, the key observation is that SleepFM consistently outperforms the supervised baselines, with its performance improving steadily as more data are used, remaining above the baseline curves for nearly all conditions. For SHHS, SleepFM surpasses the Demographics baseline in five out of six conditions across all data percentages, with particularly large improvements in smaller dataset splits. For example, SleepFM trained on just 10% of the data outperforms the Demographics baseline trained on five times more data across all conditions in SSC and four out of six conditions in SHHS (for example, cardiovascular disease death, congestive HF, myocardial infarction and stroke). SleepFM also outperforms the End-to-End PSG baseline in five out of six conditions, although the gap is slightly smaller than with the Demographics baseline. SleepFM exhibits stable scaling behavior across data percentages, with smoother performance improvements, whereas the baseline models show greater variability.

## Discussion

This study presents a large-scale foundation model for sleep analysis, developed on more than 585,000 h of PSG data from 65,000 participants. Our work makes several contributions. First, we address challenges in sleep analysis by leveraging self-supervised learning to train a foundation model that learns from unlabeled data and is agnostic to channel type and number, enabling broad exploration of sleep data across diverse clinical settings. Second, through extensive evaluation across 1,041 disease phenotypes, we demonstrate sleep’s broad predictive power for diverse health outcomes. The model shows strong performance in predicting death (C-Index 0.84), dementia (0.85), HF (0.80) and CKD (0.79). Third, we demonstrated transfer learning capabilities through strong performance on the SHHS dataset. Despite SHHS being entirely excluded from pretraining, our model maintains robust predictive power for key outcomes such as stroke (C-Index 0.81), congestive HF (0.83) and death related to cardiovascular disease (0.86). Finally, SleepFM achieves competitive performance on standard sleep analysis tasks, including sleep staging and apnea detection, with mean *F*_1_ scores ranging from 0.70 to 0.78 across cohorts—comparable to state-of-the-art models such as U-Sleep^7^, GSSC^25^, STAGES^10^ and YASA^24^. Furthermore, in a fully external validation setting, SleepFM outperforms all models on DCSM (*F*_1_ = 0.68) and is competitive with the PhysioEx^26^ models. For apnea classification, SleepFM achieves 87% accuracy in MESA, MrOS and SHHS, comparable to state-of-the-art models^8^.

SleepFM predicts all-cause mortality more accurately than both the Demographics-based model and the End-to-End PSG model, achieving a higher C-Index of 0.84 (0.81–0.87), compared to 0.79 (0.75–0.82). This indicates that pretraining efficiently captures subtle mortality-related signals in the PSG data. Research shows strong association between all-cause mortality and sleep-related factors, including high arousal burden^39^, low REM sleep^40^, sleep-disordered breathing^41^, hypoxemia and low sleep efficiency^42^. Increased ‘brain age’ derived from EEG has also been identified as an important predictor of mortality^3^. SleepFM probably integrates these multifactorial contributors, capturing respiratory events, sleep fragmentation, arousal burden and sleep efficiency, along with markers of cardiovascular, metabolic and other diseases.

Predictive and prognostic models for neurological and mental disorders are advancing rapidly, offering the potential for earlier and more individualized treatment. Among the top conditions predicted by SleepFM were Alzheimer’s disease and Parkinson’s disease, with C-Indices of 0.91 (0.87–0.98) and 0.89 (0.85–0.92), respectively. Sleep disorders are associated strongly with preclinical Alzheimer’s disease^43^, including abnormalities in non-REM sleep, such as reduced slow-wave activity^44^, REM sleep disturbances^45^ and decreased spindle activity^46^. In early Alzheimer’s disease, REM sleep abnormalities have been linked to basal forebrain cholinergic lesions, which probably contribute to cognitive decline^47^. Similarly, Parkinson’s disease is frequently preceded by REM sleep behavior disorder, characterized by REM sleep without atonia and abnormalities in BAS and ECG patterns^48^. Recent studies have also shown that respiratory signals can capture phenotypes specific to Parkinson’s disease^49^.

Consistent with these findings, SleepFM identified BAS as the strongest predictor of neurological and mental disorders, whereas respiratory signals were particularly effective in predicting senile dementia. Most studies in this domain rely on imaging modalities such as magnetic resonance imaging (MRI) and functional MRI (fMRI) to predict dementia. For example, one study using hippocampal MRI achieved a C-Index of 0.86 (ref. ^50^), whereas another using fMRI reported an AUROC of 0.82 for predicting dementia up to 9 years in advance^51^. Although direct performance comparisons are challenging due to differences in sample distributions, the ability of SleepFM to leverage PSG data to predict neurological and mental disorders underscores its potential as an alternative to imaging-based approaches.

Other established biomarkers for Alzheimer’s disease—such as amyloid PET, decreased cerebrospinal fluid β-amyloid_42_, and increased cerebrospinal fluid phosphorylated tau (for example, p-tau_129_)^52,53^—have been used widely for diagnosis and prognosis. More recently, plasma p-tau_217_ has emerged as a promising less invasive marker^54^. Sleep biomarkers from PSG data offer a complementary, noninvasive tool for the prognosis of dementia and mild cognitive impairment.

SleepFM accurately modeled cardiovascular disease in both the SSC and SHHS datasets, leveraging data-driven methods commonly used in prognostic modeling of cardiovascular disease, particularly with ECG data^55^ and lead II ECG from PSG studies^9^. Foundation models have demonstrated state-of-the-art performance with ECG data in various cross-sectional tasks^15^. For predicting cardiovascular mortality over 10 years, a previous study reported an AUROC of 0.84 (0.78–0.89) in a subset of SHHS participants with sleep apnea, whereas SleepFM achieved a slightly higher AUROC of 0.88 (0.83–0.91). Similarly, for atrial fibrillation, earlier work reported an AUROC of 0.82 (ref. ^9^), which aligns with SleepFM’s performance of 0.81 (0.78–0.84). Our ablation study further demonstrated that both ECG and respiratory signals contribute to the prediction of circulatory system phenotypes, suggesting that SleepFM integrates information on sleep apnea and heart activity in ways that are consistent with known disease mechanisms^56^.

Most disease categories, including neurological, circulatory, hematopoietic, mental disorders and endocrine/metabolic, were predicted with notably improved performance by SleepFM compared to the Demographics-based and End-to-End PSG baseline models. Many of these diseases are either associated with sleep (for example, type 2 diabetes^57^) or influenced directly by the signal modalities (for example, heart arrhythmia). Disrupted and unhealthy sleep contributes to dysfunction across several physiological systems, increasing the risk of diseases such as obesity, type 2 diabetes, hypertension, stroke and cardiovascular disease^58^. Sleep-specific conditions, including sleep apnea^56^ and less conclusively periodic leg movements^59^, are also linked to cardiovascular outcomes. Furthermore, specific EEG waveforms, such as coupled slow-wave and spindle activity, have been identified as markers of next-day blood glucose regulation^60^.

Despite these promising results, several limitations should be acknowledged. Although our dataset is large, it consists primarily of patients referred for sleep studies due to suspected sleep disorders or other medical conditions requiring overnight monitoring. This selection bias means our cohort is not representative of the general population, as people without sleep complaints or those with limited access to specialized sleep clinics are underrepresented. The model’s performance shows some degradation in temporal test sets, highlighting the challenge of maintaining predictive accuracy over time as clinical practices and patient populations evolve. Furthermore, interpreting the predictions made by SleepFM is inherently challenging due to the complexity of the learned features during training by a deep model. To mitigate this, we stratified the model’s performance across sleep stages and data modalities, and conducted evaluations on temporal test sets and unseen datasets to gain insights into its behavior. However, further work is needed to enhance case-level interpretability and understand the specific sleep patterns and features driving these predictions.

In building our model, we selected hyperparameters for SleepFM based on previous work and ensured all training converged in loss; more extensive hyperparameter searches may further boost performance. Furthermore, although we evaluated SleepFM’s transfer learning performance on an independent dataset, SHHS, only a subset of the full 1,041 conditions could be assessed in this sample due to limited diagnostic overlap with SSC; this prevented a comprehensive evaluation of generalization across the full spectrum of diseases. Our sleep apnea analysis was limited to binary and four-class classification on the basis of AHI thresholds; we did not explore more granular formulations such as AHI regression or event detection, we leave this for future research. Similarly, although SleepFM achieves competitive results on sleep staging tasks across most datasets, it lags behind specialized sleep staging models on certain external validation datasets (for example, HMC). Further specialized modeling may be necessary to optimize SleepFM for sleep staging.

This study underscores the potential of sleep-based foundation models for risk stratification and longitudinal health monitoring. By integrating several physiological signals and leveraging large-scale pretraining, SleepFM performs consistently well across diverse disease categories and outperforms supervised baselines. Its stable performance across fine-tuning splits suggests that pretraining may improve model generalizability, particularly in clinical contexts with limited labeled data. These results suggest that SleepFM can complement existing risk assessment tools and help identify early signs of diseases. As wearable sleep technologies continue to advance, models such as SleepFM may offer opportunities for noninvasive, real-time health monitoring. Future efforts should explore how combining sleep-based models with data from EHRs, omics and imaging can further enhance their utility.

## Methods

### Dataset and preprocessing

Our dataset includes PSG recordings from four different sites: SSC, BioSerenity, MESA^20,21^ and MROS^20,22^, with SHHS^20,23^ serving as an external validation dataset. Among these, MESA, MROS and SHHS are publicly available datasets, whereas SSC is our proprietary dataset. The BioSerenity dataset, provided by the BioSerenity company, contains 18,869 overnight recordings lasting 7–11 h each. This dataset is a subset of a larger collection from SleepMed and BioSerenity sleep laboratories, gathered between 2004 and 2019 across 240 US facilities^19^. At the time of this study, approximately 20,000 deidentified PSGs were available for analysis. The dataset distribution across different splits is shown in Fig. 1, with SSC constituting the largest cohort. To prevent data leakage, participants with several PSG recordings were assigned to a single split. For MESA, MROS and SHHS details, we refer readers to their original publications. Below, we describe our internal SSC dataset in more detail.

The SSC dataset comprises 35,052 recordings, each lasting approximately 8 h overnight. It includes diverse waveforms such as BAS, ECG, EMG and respiratory channels, making it a high-quality resource for sleep-related research. The dataset spans recordings from 1999 to 2024 and includes participants aged 2 to 96 years. The patient demographic statistics for SSC and BioSerenity are summarized in Extended Data Tables 1 and 2, respectively.

Our preprocessing strategy minimizes alterations to preserve raw signal characteristics crucial for identifying nuanced patterns. Each recording contains up to four modalities: BAS, ECG, EMG and respiratory, with variable numbers of channels. For BAS, we allowed up to ten channels, for ECG two channels, for EMG four channels and for respiratory seven channels. The number and type of channels vary across sites and even between patients within the same site, depending on the study type. The types of channel available across sites are described in Supplementary Tables 15–19. BAS includes channels that measure brain activity from different regions (frontal, central, occipital) as well as EOG for eye movements. EMG records electrical activity in muscles, whereas ECG captures cardiac electrical function. Respiratory channels measure chest and abdominal movements, pulse readings and nasal/oral airflow. These channels were selected based on their relevance to sleep studies, guided by sleep experts^1^.

Each PSG recording is resampled to 128 Hz to standardize sampling rates across participants and sites. Before downsampling, we utilized a fourth-order low-pass Butterworth filter to prevent aliasing, applied in a zero-phase setting to avoid phase distortion. Finally, we standardized the signal to have zero mean and unit variance. For any signals that needed to be upsampled, this was done using linear interpolation. Due to the channel-agnostic model design, we did not need any other data harmonization. Signals are segmented into 5-s patches, with each segment embedded into a vector representation for transformer model processing. To prevent data leakage, PSGs were split into pretrain, train, validation, test and temporal test sets early in the preprocessing pipeline. Although there is overlap between the pretraining and training sets, no overlap exists with the validation, test or temporal test sets. The SHHS serves as an independent dataset not used during pretraining, instead being used to evaluate the model’s ability to adapt to a new site through lightweight fine-tuning.

During pretraining, the only required labels are the modality types of the signals. A self-supervised CL objective is employed for pretraining. For downstream evaluations, we consider canonical tasks such as age/sex prediction, sleep stage classification, sleep apnea classification and various patient conditions extracted from EHR data. Sleep staging and apnea labels for SSC, MESA, MROS and SHHS were annotated by sleep experts. To ensure consistency across and within datasets, Rechtschaffen and Kales labels were converted to American Academy of Sleep Medicine standard by mapping Rechtschaffen and Kales stages 3 and 4 to American Academy of Sleep Medicine standard N3. SHHS also includes diagnostic information for conditions such as myocardial infarction, stroke, angina, congestive heart failure and death. For SSC, we paired PSG data with Stanford EHR data using deidentified patient IDs to extract demographic and diagnostic information. As BioSerenity lacks associated labels, it was used exclusively for pretraining.

### SleepFM model architecture

Our model architecture is illustrated in Fig. 1. The architecture includes several key components that differ slightly between the pretraining and fine-tuning stages. During pretraining, we employ CL as the objective function for representation learning. A single model processes all four modalities.

The first component of the architecture is the *Encoder*, a 1D convolutional neural network (CNN) that processes raw signal data for each modality separately. The encoder takes raw input vectors, where the length of each vector corresponds to a 5-s segment of the signal, referred to as a token. The input dimensions are (*B*, *T*, *C*), where *B* is the batch size, *T* is the raw temporal length of the input and *C* is the number of channels for each modality. These inputs are reshaped into (*B*, *C*, *S*, *L*), where *S* is the sequence length representing the number of tokens (*S* = *T*/*L*) and *L* corresponds to the raw vector length for a single token (for example, 640 samples). Each token is then processed individually through a stack of six convolutional layers, each followed by normalization and ELU activation layers. These layers progressively reduce the temporal resolution while increasing the number of feature channels, converting the input from 1 channel to 128 channels. After this, adaptive average pooling further reduces the temporal dimensions, and a fully connected layer compresses the representation into a 128-dimensional embedding for each token. The final output of the encoder has dimensions (*B*, *C*, *S*, *D*), where *D* = 128.

Following the encoder, a sequence of transformer-based operations is applied to extract and aggregate modality-specific and temporal features. The first step is channel pooling, which aggregates token embeddings from all channels within a given modality. This operation uses an attention pooling mechanism based on a transformer layer to compute attention scores for each channel and produces a single aggregated embedding per time segment by averaging over the channel dimension. The resulting embeddings, with dimensions (*B*, *S*, *D*), are then passed through a temporal transformer, which operates along the temporal dimension to capture dependencies between tokens. The temporal transformer applies sinusoidal positional encoding to the token embeddings, followed by two transformer blocks consisting of self-attention and feedforward layers, enabling the model to learn contextual relationships across the sequence. After temporal modeling, the embeddings are processed through temporal pooling, which aggregates token embeddings over the sequence length (*S*) for each modality. Similar to channel pooling, temporal pooling uses an attention mechanism to compute weighted averages, generating a compact representation of size (*B*, 128) per modality. These steps collectively ensure that the model captures both spatial and temporal dependencies while reducing dimensionality for computational efficiency.

The final output is a single 128-dimensional embedding for each modality, used for CL during pretraining. Whereas the 5-min recordings are used exclusively for pretraining, we retain the 5-s-level embeddings for each modality for downstream tasks such as sleep staging and disease classification.

### Baseline models

We evaluate SleepFM against two carefully chosen baseline approaches to demonstrate the value of our foundation model methodology.

The first baseline is a simple demographic model that processes only patient characteristics, including age, sex, BMI and race/ethnicity information. This demographic baseline is implemented as a one-layer MLP to establish a minimum performance threshold using only basic patient data available in most clinical settings.

The second baseline is the more sophisticated End-to-End PSG model that directly processes raw sleep recordings. This model uses the same architecture as SleepFM, including the 1D CNN encoder, channel pooling transformer block, temporal transformer block, temporal pooling transformer block and the LSTM layers, and is trained from scratch on the same dataset used for downstream evaluation. It also includes age and sex demographic features to ensure a fair comparison, but does not leverage any pretraining, serving to isolate the benefit of task-specific supervised learning on PSG signals without a foundation model.

All baseline models were trained using dataset splits shown in Table 1. The foundation model was first pretrained on the training dataset using a self-supervised objective, and subsequently fine-tuned on the same data. In contrast, the supervised baseline models were trained end-to-end without any pretraining. Although all models share identical architectures, training objectives and data splits, SleepFM consistently outperforms both baselines across a range of clinical prediction tasks. Although this may seem counterintuitive—given that the supervised PSG baseline is trained on the same data—these results align with well-established benefits of pretraining in representation learning. Self-supervised pretraining enables the model to learn more generalizable physiological representations, facilitates better convergence through improved initialization and makes more efficient use of sparse or noisy supervision during fine-tuning, as demonstrated in previous work^11^.

### Model training

Model training can be categorized into two segments: pretraining and fine-tuning. The pretraining stage involves self-supervised representation learning with a CL objective and fine-tuning involves training the model with supervised learning objective for specific tasks such as sleep stage classification, sleep apnea classification and disease prediction. We describe these in more details below.

#### Pretraining

Model pretraining is performed using a self-supervised learning objective called CL. Specifically, we employ a CL objective for several modalities, referred to as LOO-CL. The key idea behind CL is to bring positive pairs of embeddings from different modalities closer in the latent space while pushing apart negative pairs. Positive pairs are derived from temporally aligned 5-min aggregated embeddings, obtained after temporal pooling, across four different modalities. All other nonmatching instances within a training batch are treated as negative pairs.

In LOO-CL, we define a predictive task where an embedding from one modality attempts to identify the corresponding embeddings from the remaining modalities. For each modality *i*, we construct an embedding ${\bar{x}}_{k}^{-i}$ by averaging over embeddings from all other modalities, excluding modality *i*. We then apply a contrastive loss between the embedding of modality *i* and this LOO representation:

$${{\mathcal{L}}}_{i,k}=-\log \frac{\exp \left({\rm{sim}}({x}_{k}^{i},{\bar{x}}_{k}^{-i})/\tau \right)}{\mathop{\sum }\nolimits_{m = 1}^{N}\exp \left({\rm{sim}}({x}_{k}^{i},{\bar{x}}_{m}^{-i})/\tau \right)},$$

where ${{\mathcal{L}}}_{i,k}$ is the loss for a sample *k* from modality *i* in a given batch, ${\rm{sim}}(\cdot )$ represents a similarity function (for example, cosine similarity) and *τ* is a temperature scaling parameter. The numerator computes the similarity between the embedding of modality *i* and the LOO representation of the corresponding sample, whereas the denominator sums the similarities across all samples within the batch. The motivation behind the LOO method is to encourage each embedding to align semantically with all other modalities.

#### Fine-tuning

After pretraining with the CL objective, we extract 5-s embeddings for all patient PSG data across modalities. We standardize the temporal context to 9 h for all patients—longer recordings are cropped and shorter ones are zero-padded to ensure consistent input dimensions. For example, for a patient’s standardized 9-h sleep data, the resulting patient matrix has dimensions (4 × 6,480 × 128), where 4 represents the number of modalities, 6,480 is the number of 5-s embeddings for 9 h of sleep and 128 is the embedding vector dimension.

During fine-tuning, we first apply a channel pooling operation across different modalities, reducing the dimensions to (6,480 × 128) for our example patient matrix. The pooled embeddings are then processed through a two-layer LSTM block, which is designed to handle temporal sequences. For sleep staging tasks, these 5-s embeddings are passed directly through a classification layer. For all other tasks, the embeddings are first pooled along the temporal dimension before being passed through an output layer.

For disease classification, we append age and sex features to the mean-pooled embedding vector after the LSTM block, before passing it to the final output layer. This addition empirically improves performance and surpasses the demographic baseline.

The fine-tuning objective for disease prediction uses the CoxPH loss function—a standard approach in survival analysis for modeling time-to-event data. The CoxPH loss maximizes the partial likelihood and is defined for a single label as:

$${{\mathcal{L}}}_{{\rm{CoxPH}}}=-\frac{1}{{N}_{e}}\mathop{\sum }\limits_{i=1}^{n}{\delta }_{i}\left({h}_{i}-\log \sum _{j\in R({t}_{i})}\exp ({h}_{j})\right),$$

where *h*_*i*_ is the predicted hazard for the *i*th patient, *δ*_*i*_ is the event indicator (1 for event occurrence, 0 otherwise), *t*_*i*_ is the event or censoring time, *R*(*t*_*i*_) represents the risk set of all patients with event times greater than or equal to *t*_*i*_, *n* is the total number of patients and ${N}_{e}=\mathop{\sum }\nolimits_{i = 1}^{n}{\delta }_{i}$ is the number of events.

For our multilabel setup with 1,041 labels, we extend the CoxPH loss by computing it independently for each label and summing the results:

$${{\mathcal{L}}}_{{\rm{total}}}=\mathop{\sum }\limits_{k=1}^{L}{{\mathcal{L}}}_{{\rm{CoxPH}}}^{(k)},$$

where *L* is the total number of labels.

Given the large dataset size, computing the loss for all patients in a single batch is computationally infeasible. Therefore, we calculate the loss in smaller batches of 32 samples, with patients sorted by event time in descending order to ensure correct computation of the partial likelihood. This batching strategy, combined with the summation of per-label losses, provides an efficient and scalable approach for multilabel time-to-event modeling.

#### Architectural details

We provide additional implementation-level details to clarify how SleepFM is constructed and trained. The design of SleepFM was developed through an empirical and iterative process, informed by domain knowledge and guided by practical training considerations. Although we did not perform an exhaustive hyperparameter search, we systematically evaluated architectural variants through trial-and-error by monitoring loss convergence, training stability and downstream performance.

Each 5-s segment of raw PSG signals (640 timepoints at 128 Hz) is passed through a tokenizer composed of six convolutional layers with increasing feature maps: 1 → 4 → 8 → 16 → 32 → 64 → 128. Each convolutional block includes BatchNorm, ELU activation and LayerNorm. After convolution, adaptive average pooling reduces the temporal axis to 1, and a linear layer projects the features to a fixed 128-dimensional token embedding. The resulting output shape is (*B*, *C*, *S*, 128), where *C* is the number of channels and *S* is the number of 5-s tokens.

To accommodate variability in the number and ordering of channels across different PSG datasets, we introduced an attention-based spatial pooling layer that operates across channels using a transformer encoder. This design makes the model robust to inconsistencies in recording configurations across sites. Specifically, embeddings from several channels within a modality are pooled using multihead self-attention, producing a modality-specific sequence of shape (*B*, *S*, 128).

To capture long-range temporal dependencies in sleep signals, the pooled token sequence is passed through three transformer encoder layers (each with eight heads, batch-first configuration and a dropout rate of 0.3), along with sinusoidal positional encoding and LayerNorm. This component enables modeling of contextual relationships across the sleep sequence. The output shape remains (*B*, *S*, 128).

An additional attention-based pooling layer aggregates the temporal sequence across timesteps, resulting in a single 128-dimensional embedding for each modality (for example, BAS, ECG, EMG or respiratory). These fixed-size modality-specific embeddings are used for pretraining with a self-supervised CL objective.

For downstream disease prediction, 5-s token embeddings spanning a standardized 9-h window are processed by a fine-tuning head. This head includes spatial pooling followed by a two-layer bidirectional LSTM (hidden size: 64). Temporal mean pooling is applied across valid timesteps, and normalized age and sex features are concatenated with the pooled output. The combined vector is then passed through a final linear layer to generate hazard scores for each disease. The total number of learnable parameters in this setup is approximately 0.91 million.

The supervised baseline model uses the same architecture as SleepFM but is trained from scratch without pretraining. The demographics-only baseline passes four input features—age, sex, BMI and race/ethnicity—through a shallow MLP with dimensions 4 → 128 → output.

#### Implementation details

All implementations were carried out using PyTorch, a library used widely for deep learning. The PSG data was gathered and processed within a HIPAA-compliant and secure compute cluster on Google Cloud Platform. Patient EHR data was likewise stored and analyzed exclusively within this secure environment.

For pretraining, the model was trained with a batch size of 32, a learning rate of 0.001, eight pooling heads, three transformer layers and a dropout rate of 0.3. As previously described, each patch size corresponds to a 5-s segment, and the total sequence length is 5 min for the transformer model. The total parameter count for the model was approximately 4.44 million. Pretraining was performed on 432,000 h of sleep data collected from 48,000 participants for one epoch, using an NVIDIA A100 GPU. The entire pretraining process took approximately 15 h.

For fine-tuning, the batch size was also set to 32, with a learning rate of 0.001, four pooling heads, two LSTM layers and a dropout rate of 0.3. The fine-tuned model had approximately 0.91 million learnable parameters. Training was conducted on patient data, with each token embedding represented as a 128-dimensional vector, over ten epochs. The fine-tuning process was performed on an NVIDIA A100 GPU, with the total training time per epoch ranging from 2 to 5 min, depending on the task.

All data analysis and preprocessing were performed using Python (v.3.10.14) and its data analysis libraries, including Pandas (v.2.1.1), NumPy (v.1.25.2), SciPy (v.1.11.3), scikit-survival (v.0.23.0), scikit-learn (v.1.5.2) and PyTorch (v.2.0.1).

### Reporting summary

Further information on research design is available in the Nature Portfolio Reporting Summary linked to this article.

## Online content

Any methods, additional references, Nature Portfolio reporting summaries, source data, extended data, supplementary information, acknowledgements, peer review information; details of author contributions and competing interests; and statements of data and code availability are available at https://doi.org/10.1038/s41591-025-04133-4.

## Supplementary information

Supplementary Information Supplementary Figs. 1–12 and Tables 1–19.

Reporting Summary

## Figures and Tables

**Extended Data Table 1:**
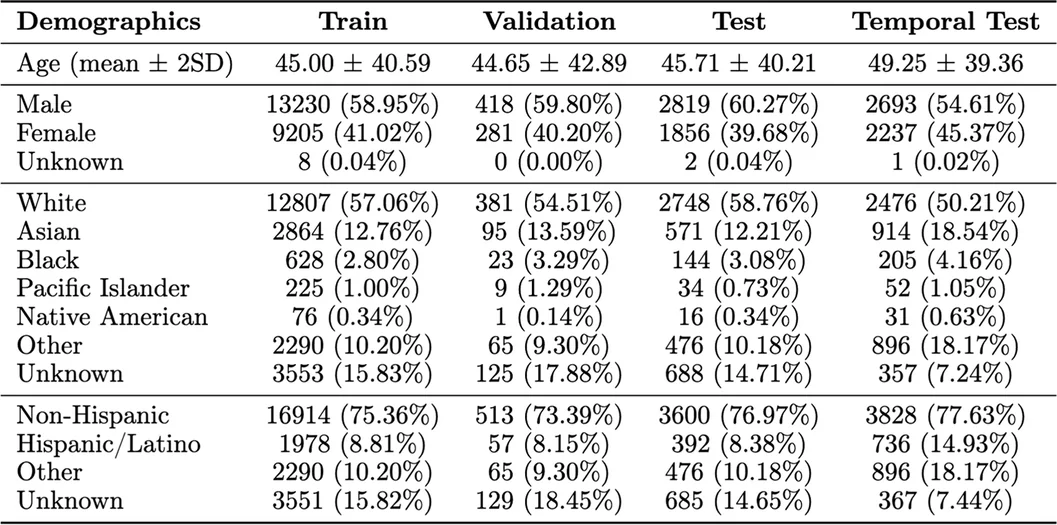
Demographic characteristics of the Stanford Sleep Clinic (SSC) cohort

Values are shown by dataset split and reported as mean +- 2 standard deviations for age, and as counts (percentages) for categorical variables.

**Extended Data Table 2:**
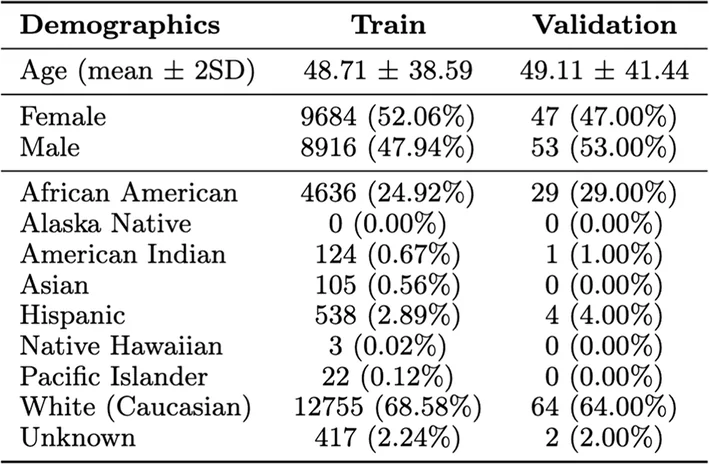
Demographic characteristics of the Bioserenity cohort

Values are presented as mean +- 2 standard deviations for age, and as counts (percentages) for categorical variables.

**Extended Data Table 3:**
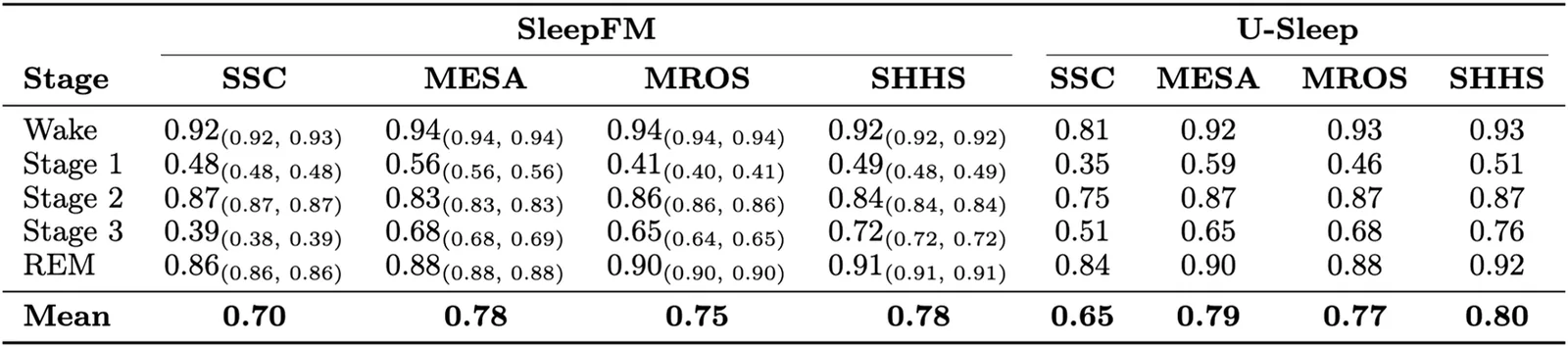
Per-sleep-stage F1 performance of SleepFM across four cohorts

Per-sleep-stage F1 scores for SleepFM across four cohorts (SSC, MESA, MrOS and SHHS), with comparison to U-Sleep. Values for SleepFM are mean F1 with 95% confidence intervals from 1,000 bootstrap resamples of test recordings (CIs shown in parentheses beneath each estimate). The bold ‘Mean’ row reports the macro-average across stages. U-Sleep values are the corresponding F1 scores reported for the same cohorts; confidence intervals were not available.

**Extended Data Table 4:**
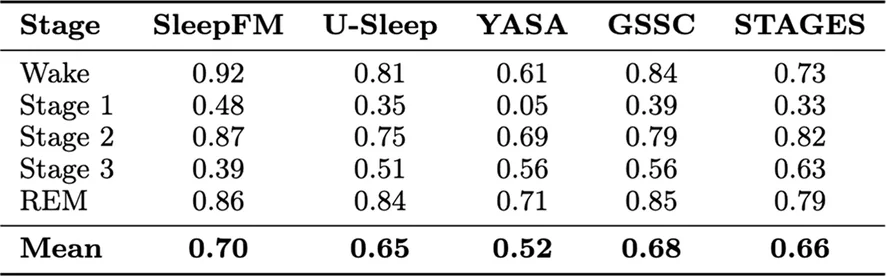
Sleep staging performance on the SSC cohort

Sleep staging results (F1) for SleepFM, U-Sleep, YASA, GSSC, and STAGES on the SSC cohort. The bold bottom row reports the macro-average (mean) across stages.

**Extended Data Table 5:**
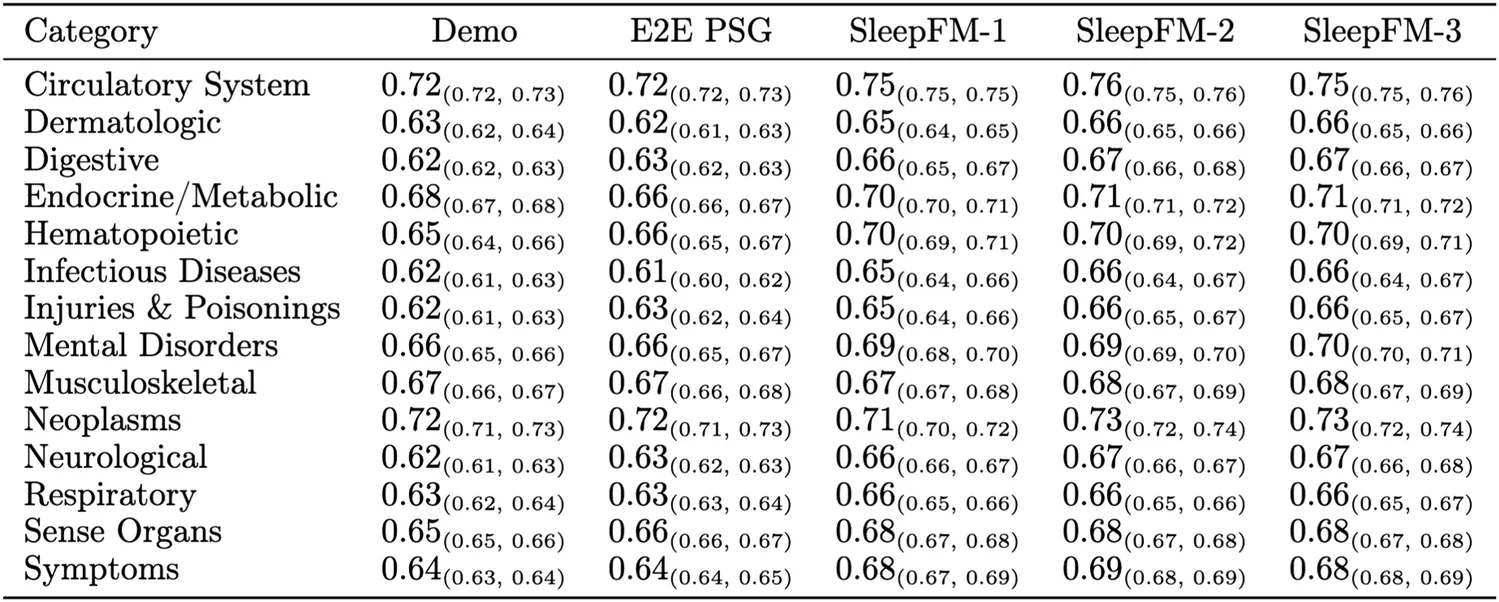
Comparison of category-averaged C-Index across SleepFM baseline

Category-averaged C-Index (mean with 95% CI) comparing SleepFM variants with two baselines across disease categories on Stanford cohort (n=5,019). The Demographics baseline (‘Demo’) uses only structured clinical features (age, sex, BMI, and race/ethnicity). The End-to-End PSG baseline (‘E2E-PSG’) is trained directly on raw PSG signals with age and sex, without any pretraining. SleepFM-1 denotes SleepFM-LSTM (w/o Demo), using two LSTM layers in the fine-tuning prediction module and no demographic features. SleepFM-2 denotes SleepFM-Linear, a linear prediction module on SleepFM embeddings with age and sex. SleepFM-3 denotes SleepFM-LSTM, which uses two LSTM layers in the fine-tuning prediction module with age and sex. Values are averaged within each category across conditions. Uncertainty is estimated via nonparametric bootstrapping (n=1000 resamples): for each resample, conditions within a category are sampled with replacement and the category mean is computed; 95% CIs are the 2.5th-97.5th percentiles across resamples.

## Data Availability

Of the five data sources used in this study, four datasets are available publicly and can be accessed at the following links: SHHS (https://sleepdata.org/datasets/shhs), MrOS (https://sleepdata.org/datasets/mros), MESA (https://sleepdata.org/datasets/mesa) and SSC (https://sleepdata.org/datasets/ssc). The BioSerenity dataset is proprietary and owned by BioSerenity, which has granted Stanford University access under a research and development agreement; please contact BioSerenity directly for data agreement. Stanford sleep data is available upon publication at https://bdsp.io/content/hsp/2.0/. Access to these data is provided solely for research purposes and is subject to data use restrictions that prohibit redistribution or sharing with third parties.

## Code availability

All of the SleepFM code is open source and available at https://github.com/zou-group/sleepfm-clinical.
