# Supplementary material for: A multimodal sleep foundation model for disease prediction
Source: Nat Med. 2026 Jan 6;32(2):752–62. doi: 10.1038/s41591-025-04133-4 (PMC12920147; doi:10.1038/s41591-025-04133-4)
Supplement: Supplementary file 1 — Supplementary Figs. 1–12 and Tables 1–19. [file 41591_2025_4133_MOESM1_ESM.pdf]

nature medicine

Supplementary information https://doi.org/10.1038/s41591-025-04133-4

A multimodal sleep foundation model for disease prediction

In the format provided by the authors and unedited

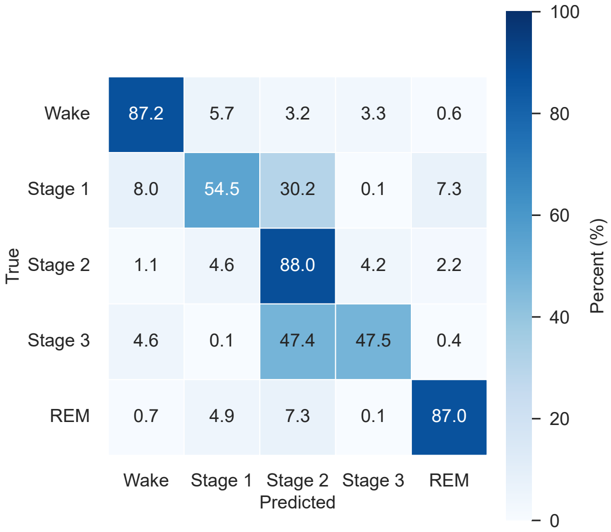

(a) SSC

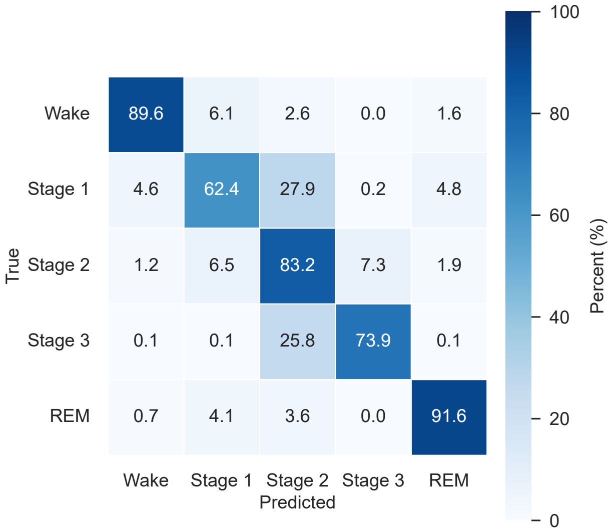

(b) MESA

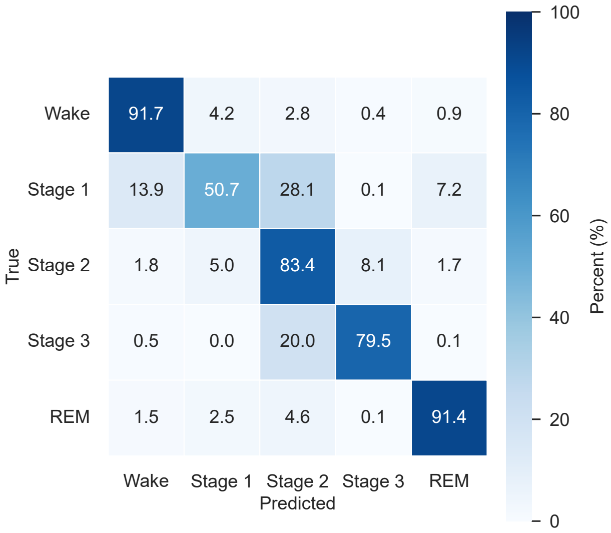

(c) MROS

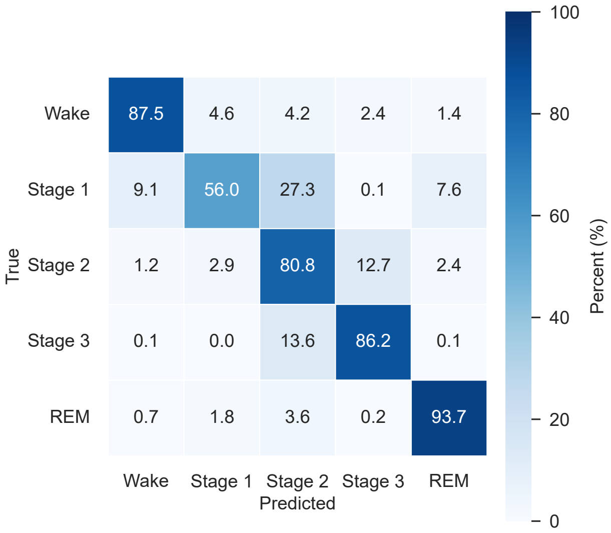

(d) SHHS

Supplementary Figure 1. SleepFM sleep staging confusion matrices for the Stanford, MESA, MROS, and SHHS cohorts. SleepFM generally performs well in differentiating between sleep stages, with mild confusion observed between Stage 1 and Stage 2, as well as between Stage 2 and Stage 3.

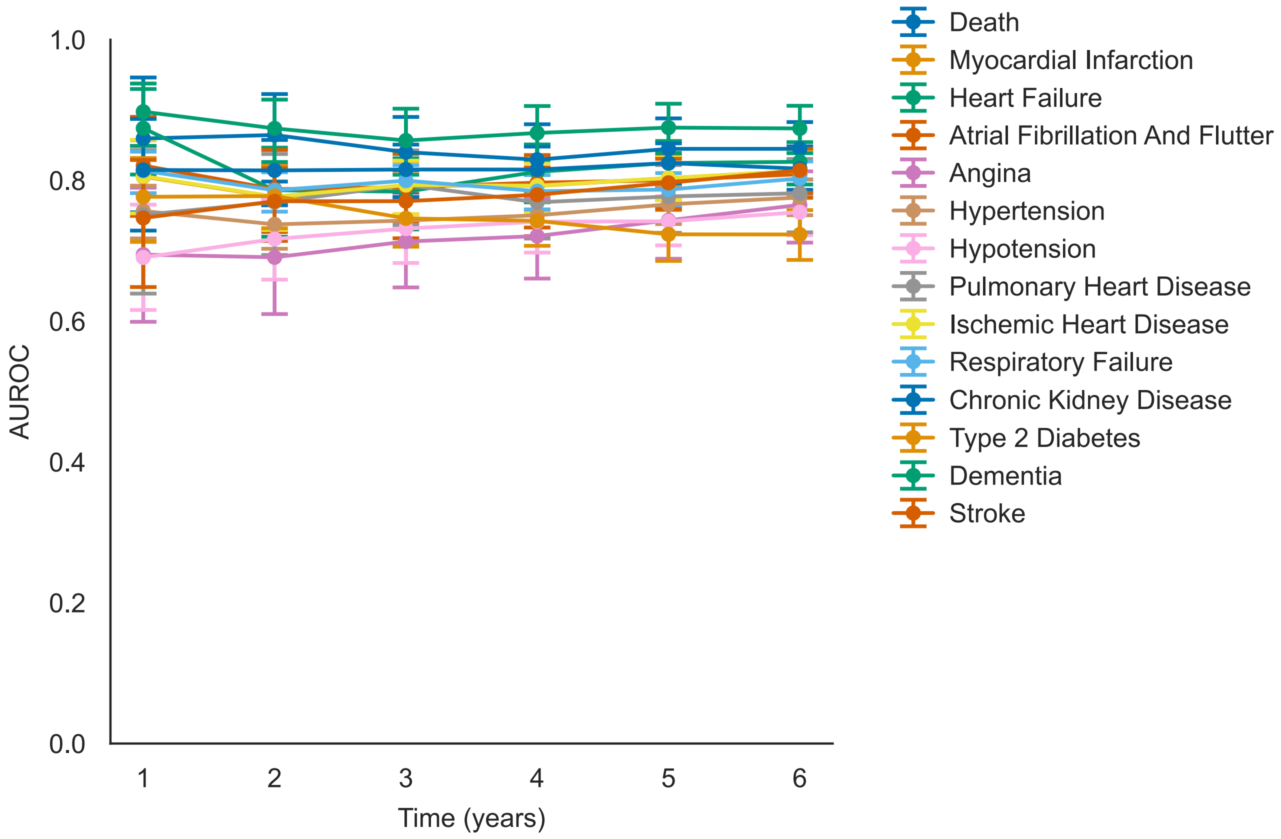

Supplementary Figure 2. AUROC trends across prediction windows from 1 to 6 years. While we report 6-year AUROC as our primary metric to accommodate the range of conditions—both acute and chronic—we acknowledge that different time horizons may be more appropriate for specific conditions. The plot above demonstrates that AUROC remains robust across multiple conditions over the 1–6 year range.

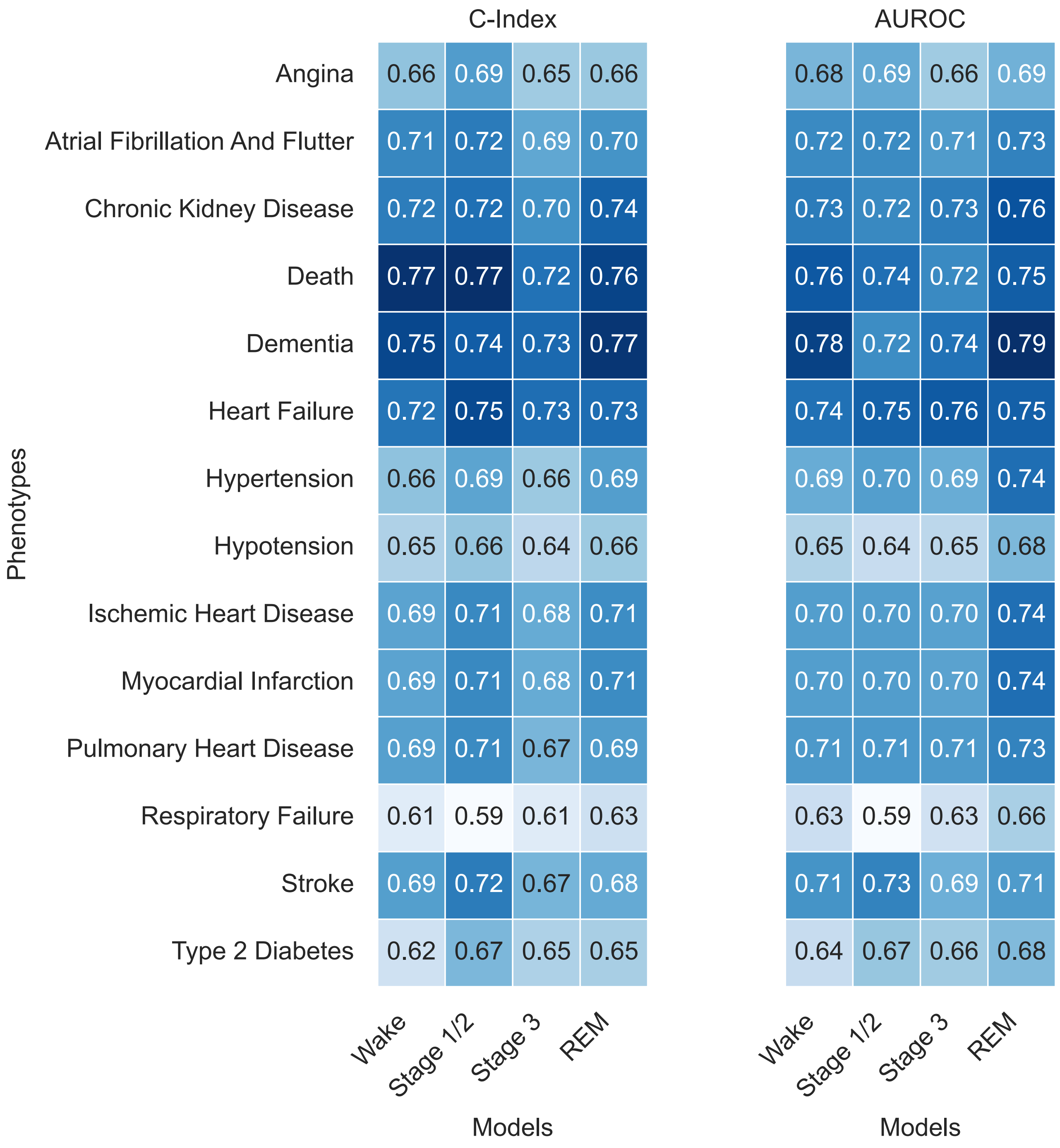

Supplementary Figure 3. Performance comparison of models trained on different sleep stages across selected conditions. Metrics presented are C-Index and AUROC.

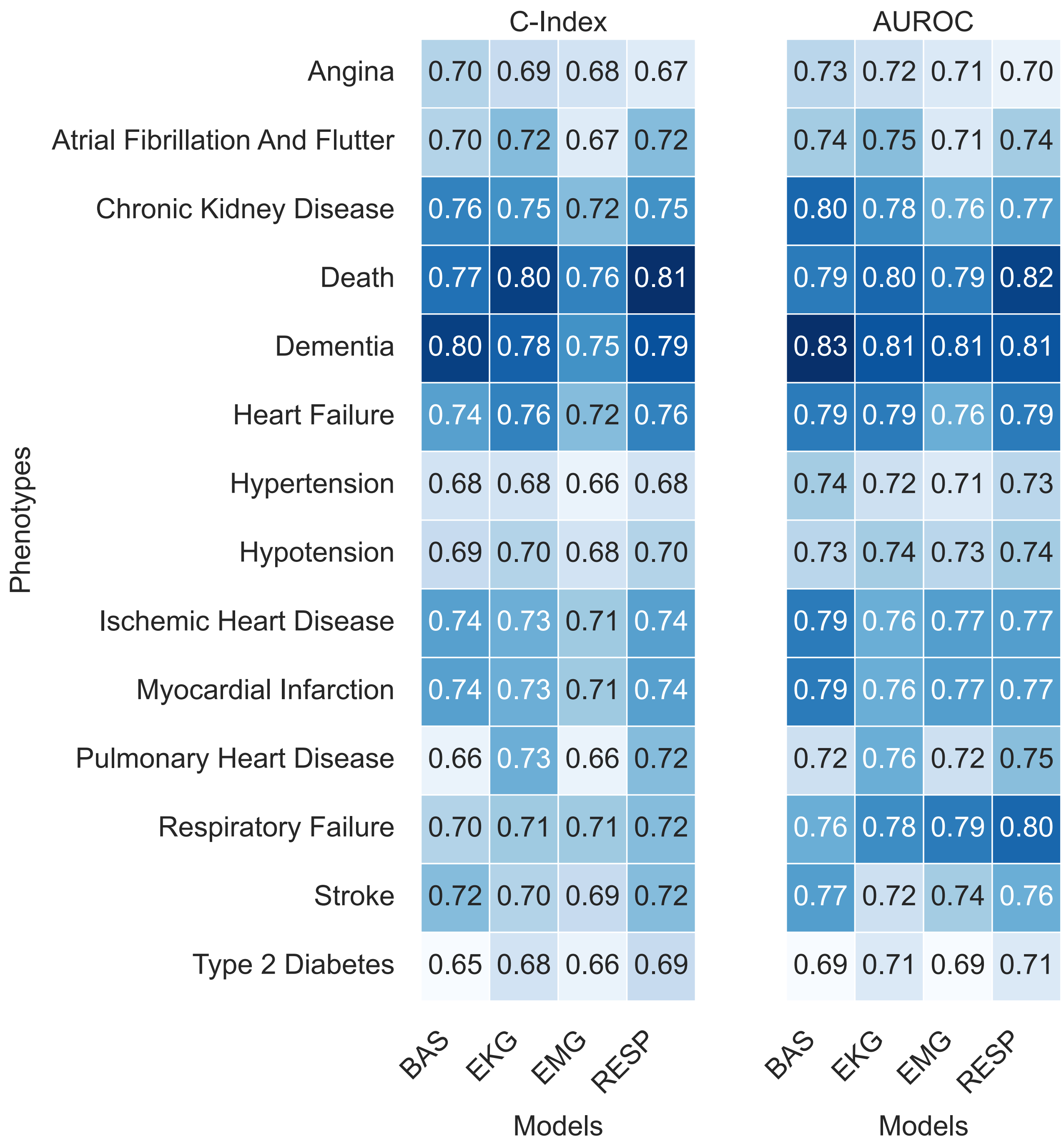

Supplementary Figure 4. Performance comparison of models trained on different sleep modalities across selected conditions. BAS, EKG, and RESP exhibit similar predictive performance across various conditions, while EMG is the least predictive.

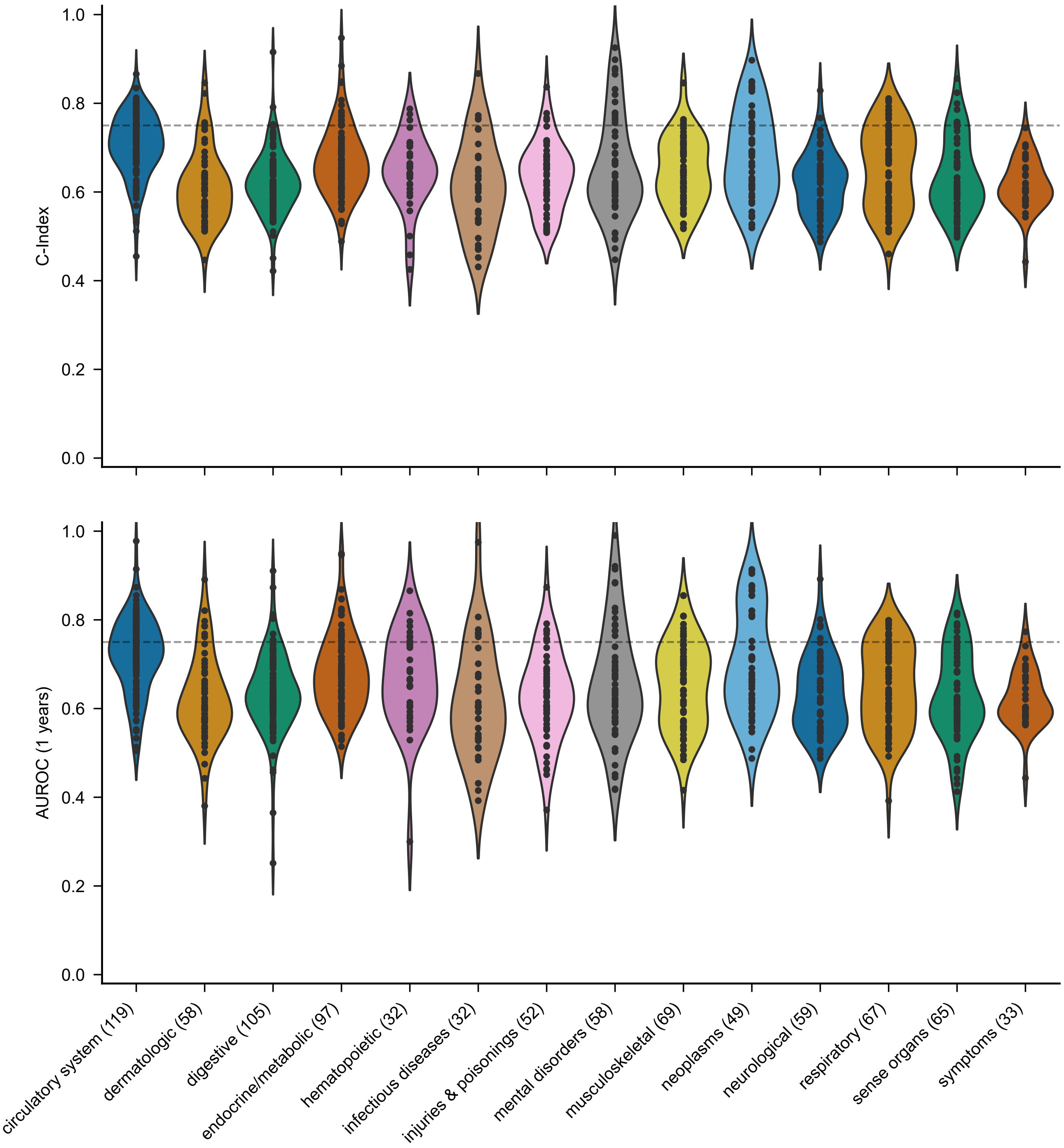

Supplementary Figure 5. SleepFM performance across disease categories on the temporal test set (Stanford data from 2020 onwards). Performance is measured using C-Index and 6-year AUROC metrics to assess the model's robustness to temporal distribution shifts. Despite these shifts, SleepFM maintains strong performance across a broad spectrum of diseases.

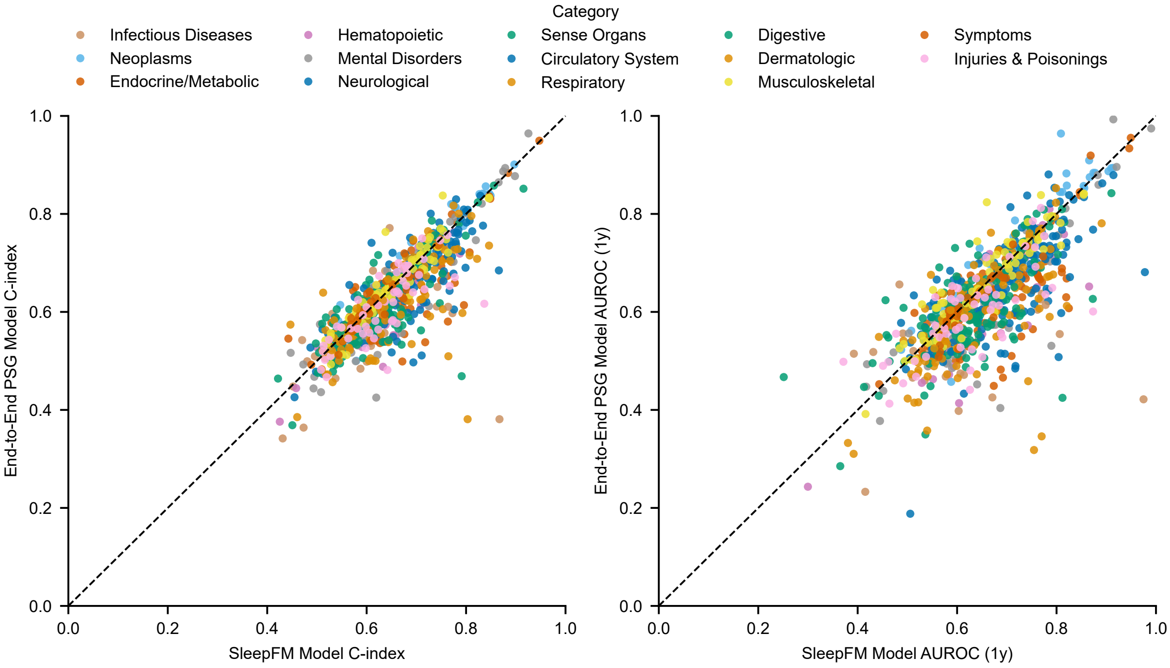

(a) Comparison with demographics-only baseline

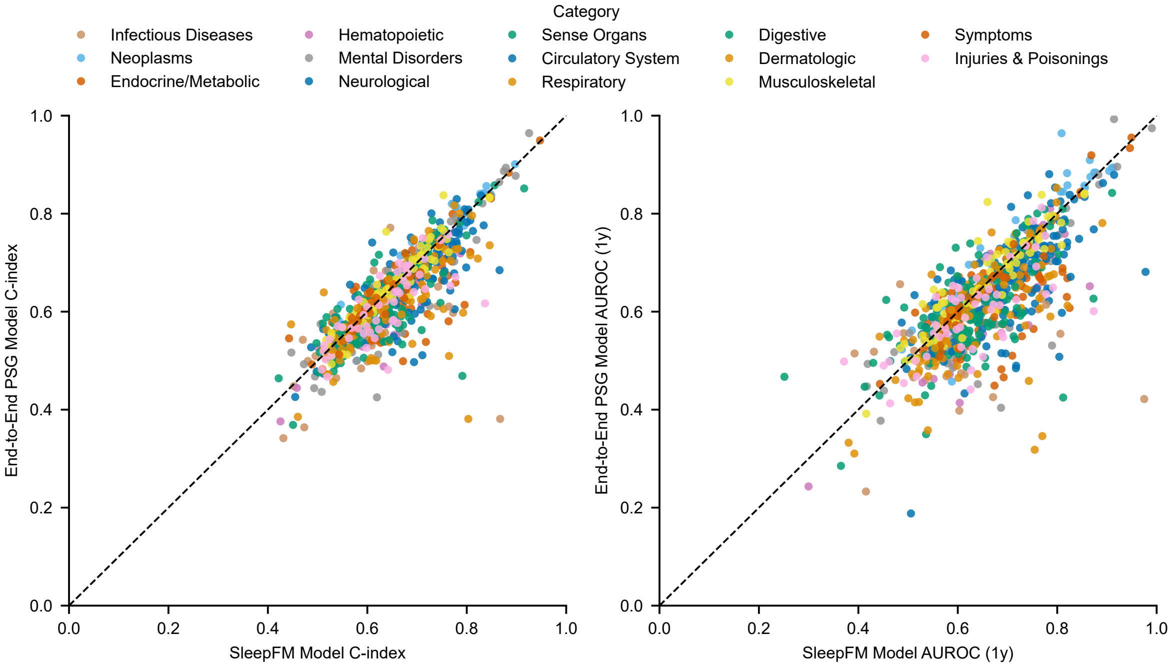

(b) Comparison with End-to-End PSG model

Supplementary Figure 6. Performance comparison on the temporal test set (Stanford data from 2020 onwards) using C-Index and 6-year AUROC metrics. Each point represents a disease phenotype. (a) Comparison against demographics baseline shows points below the diagonal, indicating SleepFM's superior performance. (b) Comparison against End-to-End PSG model demonstrates the benefits of foundation model pre-training even under temporal distribution shift. These results highlight SleepFM's robust generalization to more recent patients. Overall, most points fall below the diagonal, demonstrating that SleepFM outperforms the baseline models for the majority of conditions.

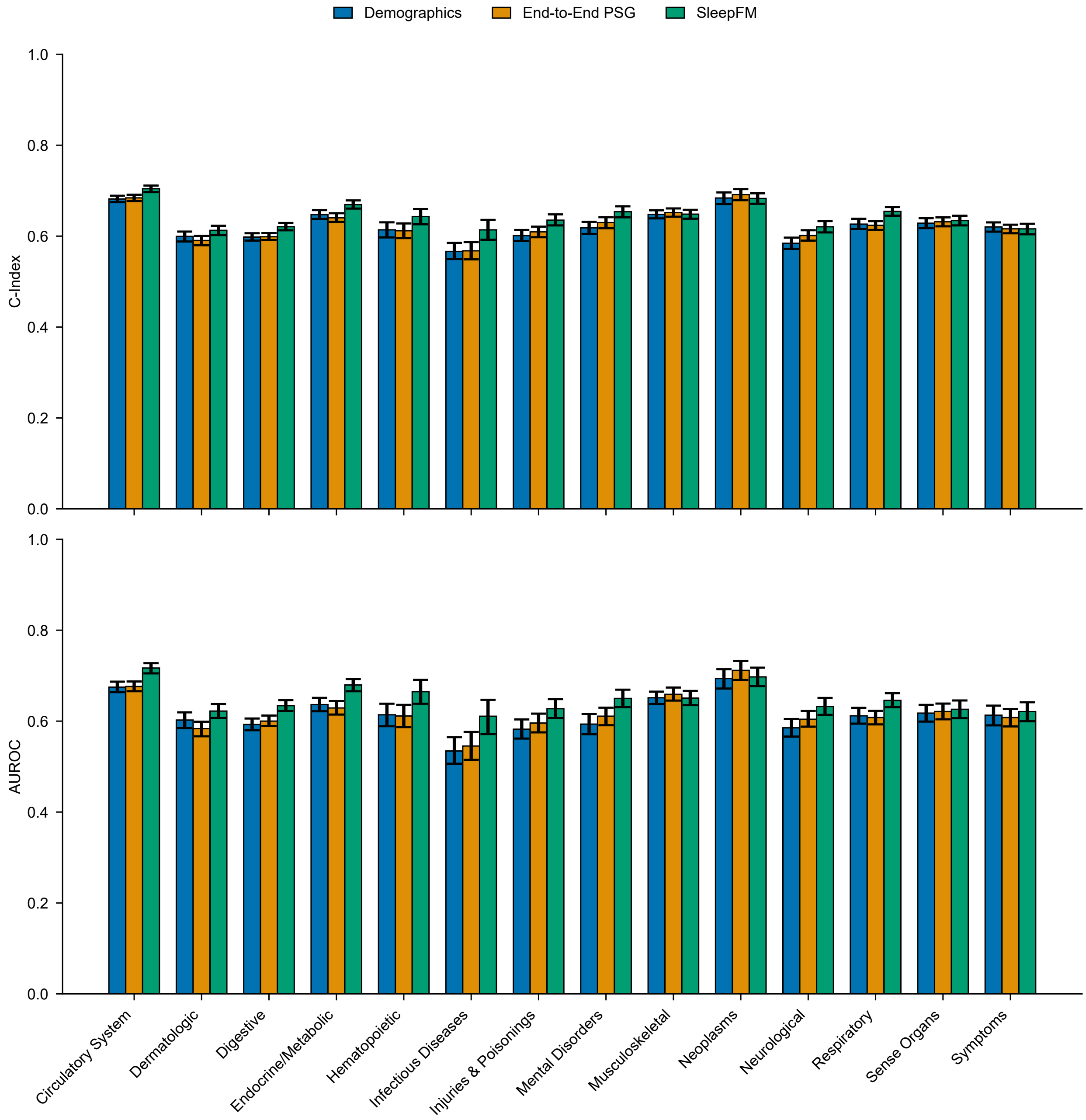

Supplementary Figure 7. Performance comparison of SleepFM with baseline models on the temporal test set (2020 onwards), showing average C-Index and 6-year AUROC metrics across disease categories. The demographics baseline uses only clinical features (age, gender, BMI, and race/ethnicity), while the End-to-End PSG baseline is trained directly on raw PSG signals without pre-training. Despite the temporal distribution shift, SleepFM maintains its performance advantages over both baselines across most disease categories, demonstrating robust generalization to more recent patients.

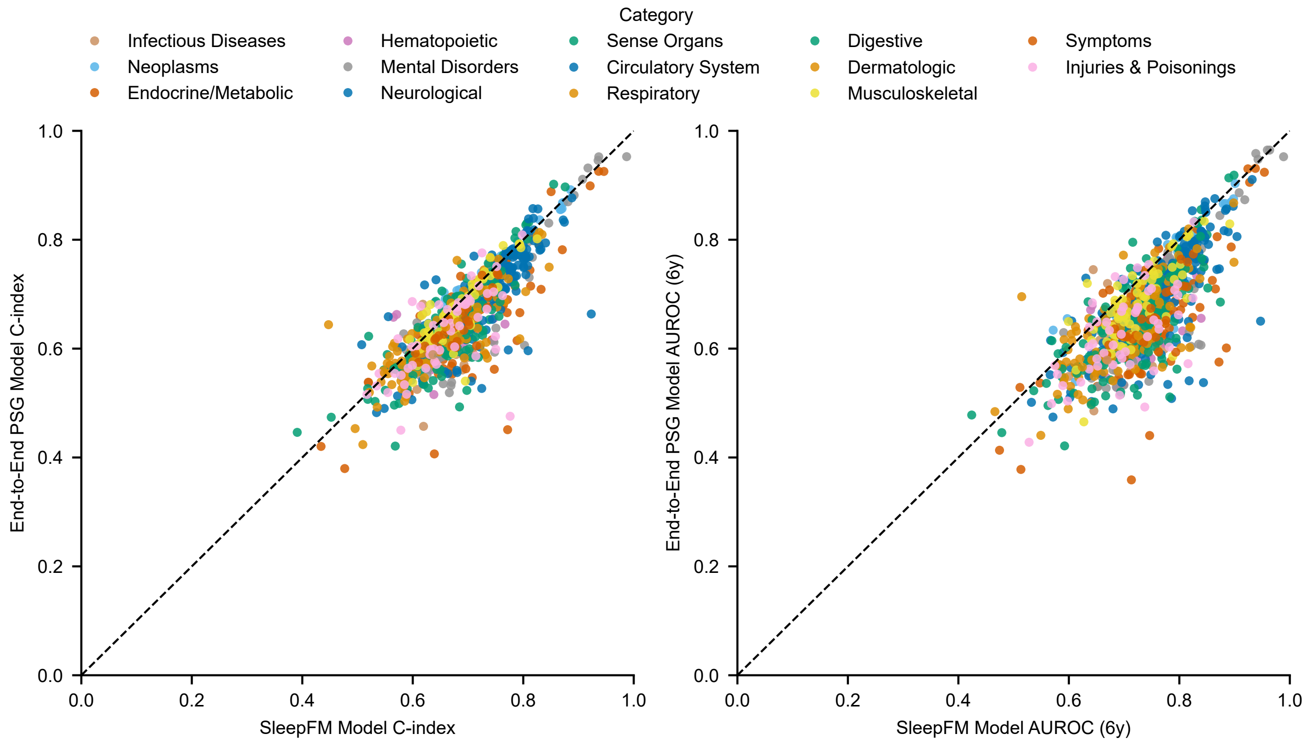

(a) Comparison with demographics-only baseline

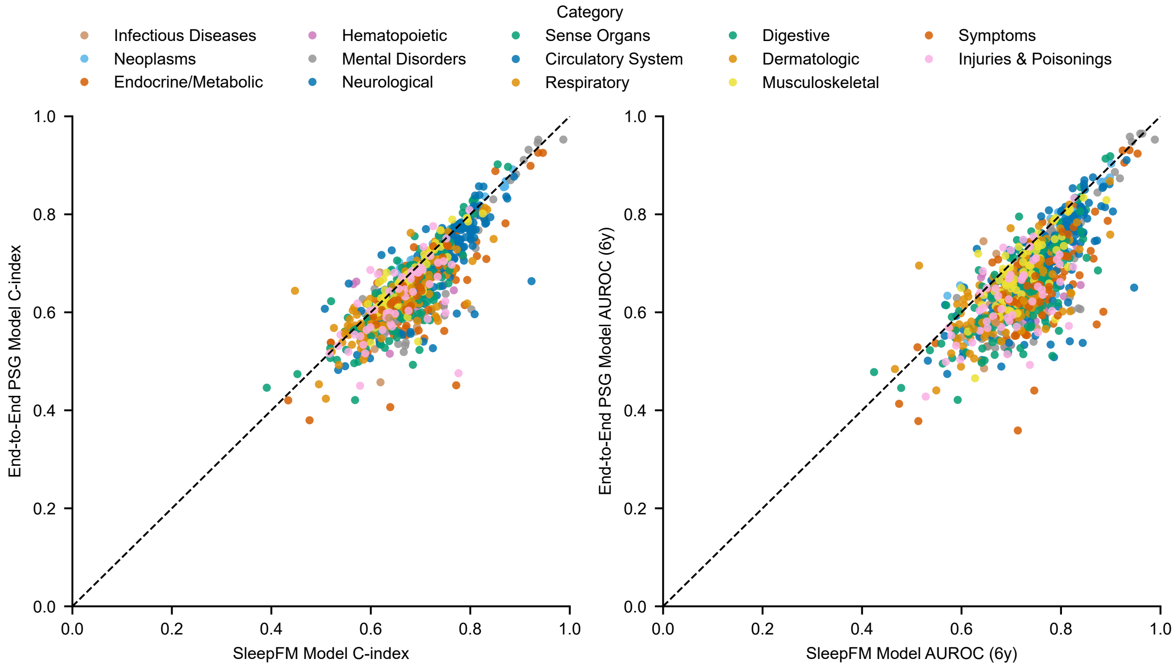

(b) Comparison with End-to-End PSG model

Supplementary Figure 8. Performance comparison of SleepFM with baseline models on the test set using C-Index and 6-year AUROC metrics. Each point represents a disease phenotype. (a) Comparison against the demographics-only baseline shows points consistently below the diagonal, indicating superior performance by SleepFM. (b) Comparison against the End-to-End PSG model highlights the advantages of foundation model pre-training over direct supervised learning from raw PSG signals. Overall, most points fall below the diagonal, demonstrating that SleepFM outperforms the baseline models for the majority of conditions.

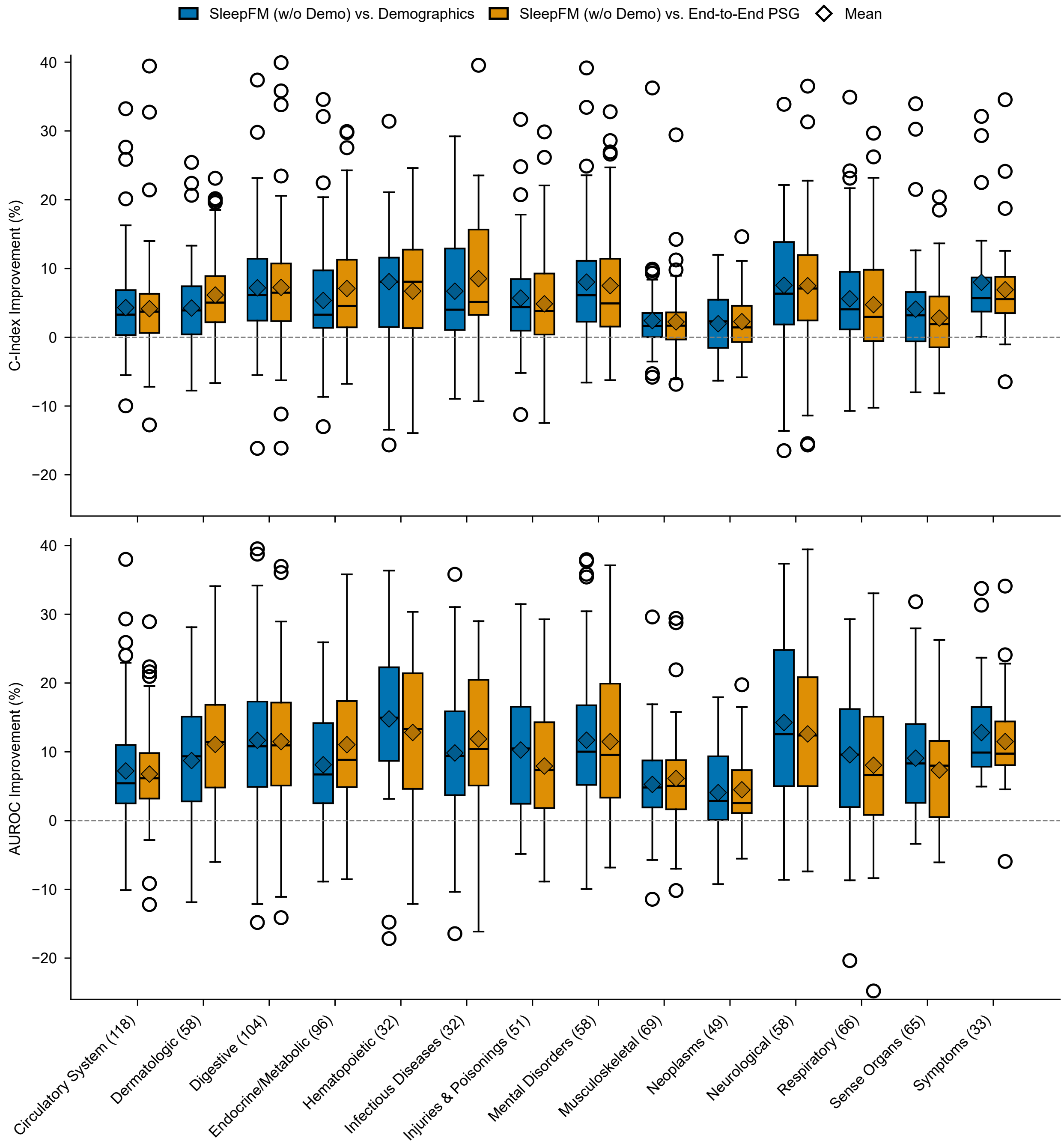

Supplementary Figure 9. Performance improvements of SleepFM over baseline models across disease categories. This version of SleepFM excludes demographic inputs, while the End-to-End PSG model includes age and sex. Results show percentage improvements in C-Index and 6-year AUROC on the test set. Compared with the demographics-only model (age, sex, BMI, and race/ethnicity), SleepFM achieves broad gains, especially for neurological and hematopoietic conditions. The comparison with the End-to-End PSG model underscores the value of foundation model pre-training for robust sleep representations. Each box shows the distribution of disease-level percentage improvements by category, with C-Index (top) and AUROC (bottom). Boxes indicate interquartile ranges (IQR), whiskers extend to 1.5×IQR, outliers are points, and diamonds mark mean improvements. The dashed line at zero denotes no improvement.

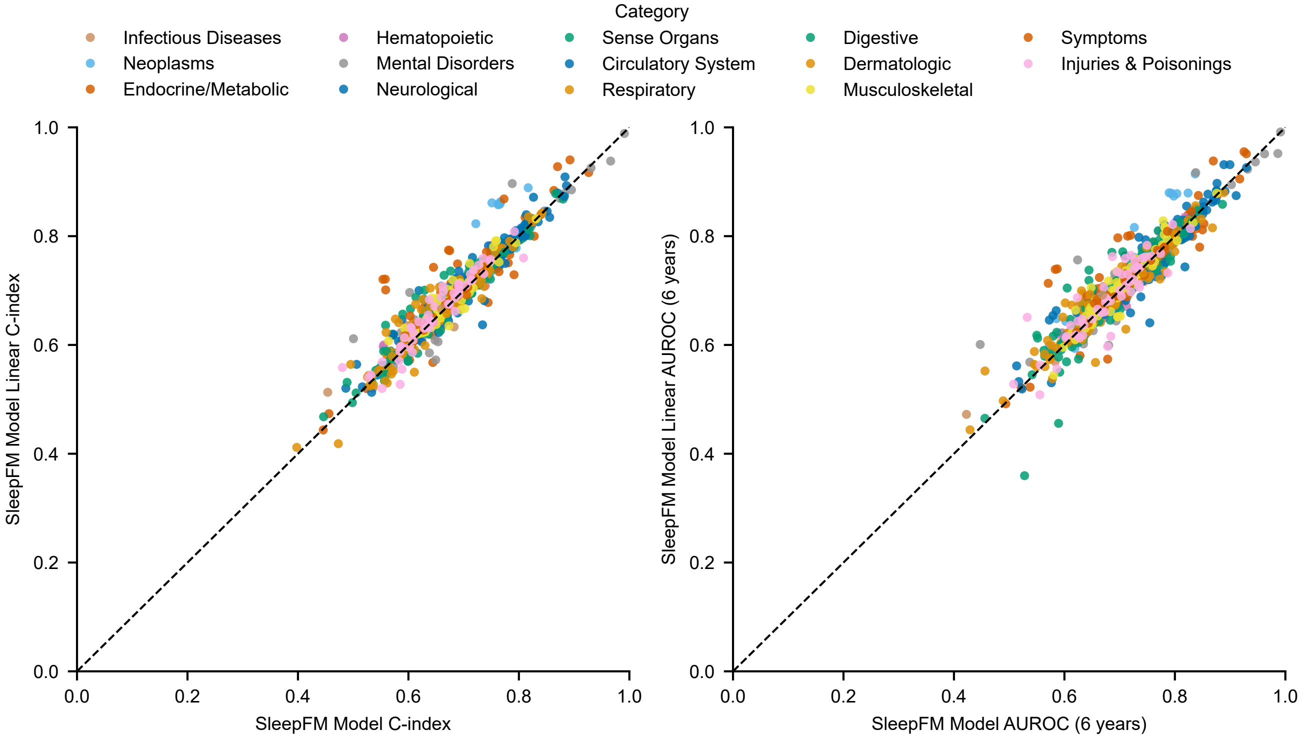

(a) Comparison between LSTM and linear finetuning heads for SleepFM.

Supplementary Figure 10. Performance comparison between different finetuning heads for SleepFM using C-Index and 6-year AUROC metrics. Each point represents a disease phenotype. The LSTM-based head shows only a modest advantage over the linear classifier, with an average improvement of 1.98% in C-Index and 1.72% in AUROC. This suggests that the strength of SleepFM lies primarily in the expressiveness of its pretrained embeddings, rather than complexity in the downstream architecture.

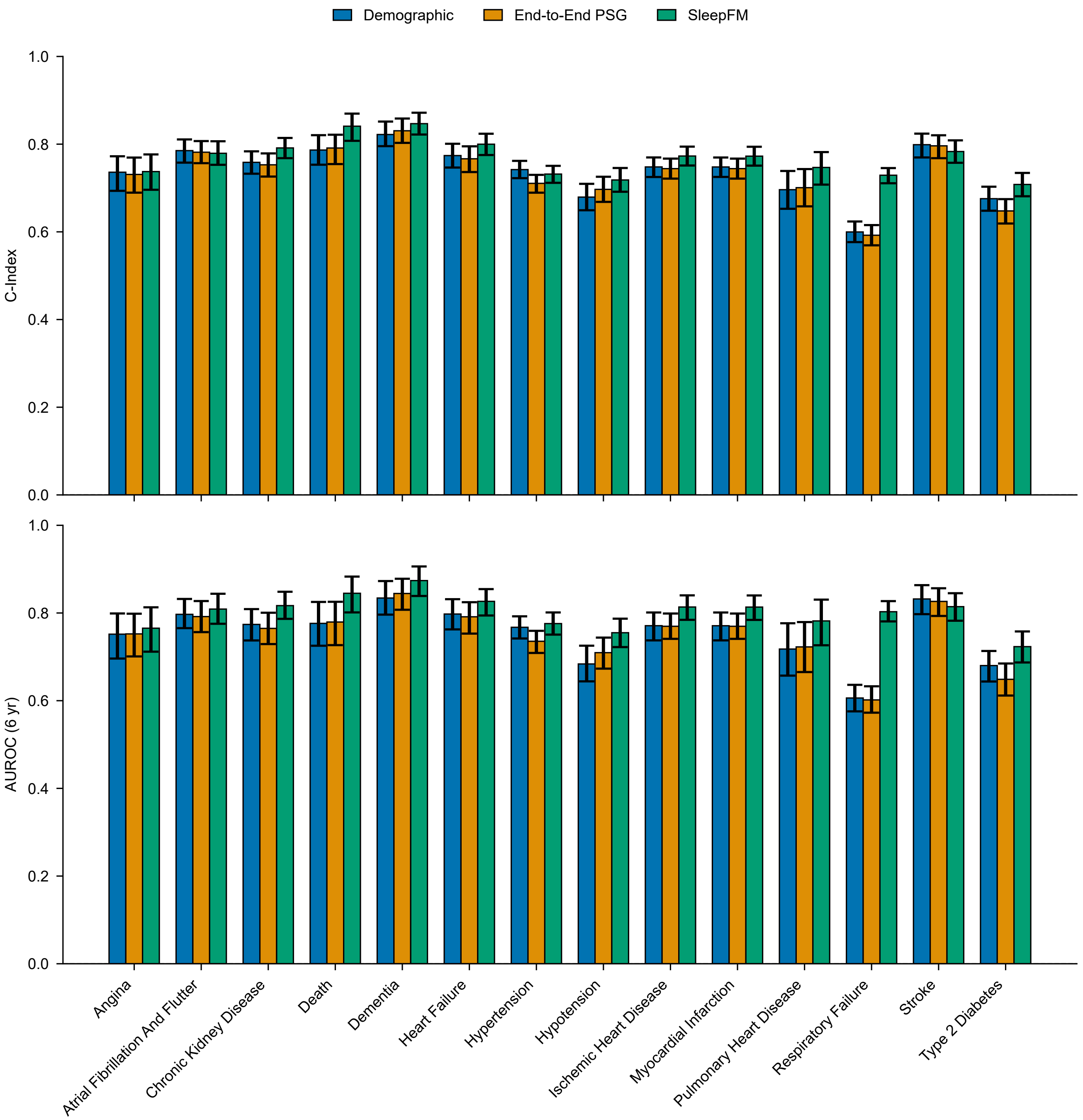

Supplementary Figure 11. Performance comparison across clinically relevant diseases selected in consultation with sleep experts. The plot compares SleepFM against two baselines: a demographics-only model using clinical features (age, gender, BMI, and race/ethnicity) and an End-to-End PSG model trained directly on raw PSG signals. Performance is evaluated on the test set using multiple metrics: C-Index and 6-year AUROC. The selected conditions include critical health outcomes such as death, heart failure, stroke, and dementia. SleepFM demonstrates superior predictive performance across these important clinical endpoints compared to both baseline approaches.

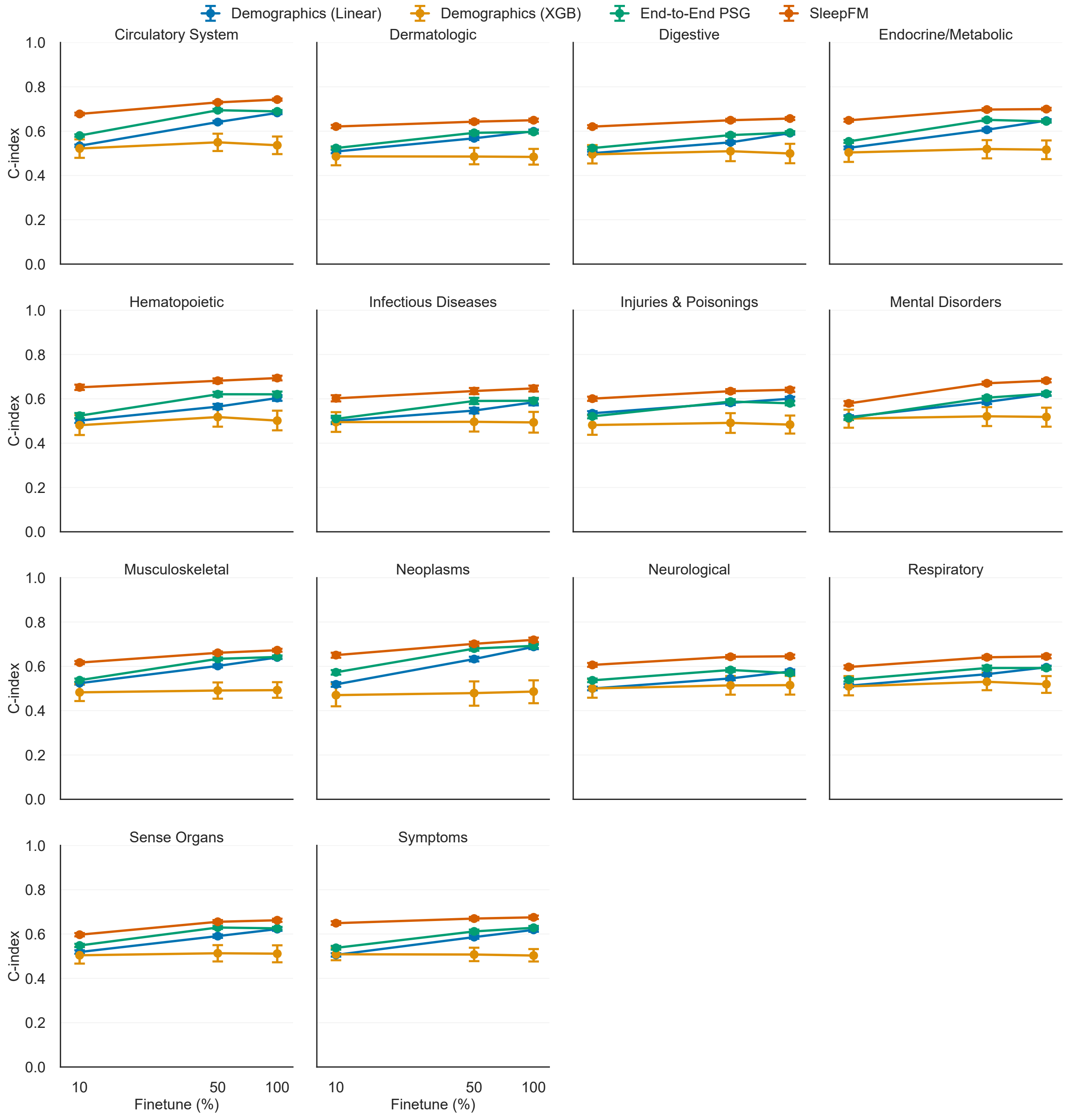

Supplementary Figure 12. Scaling behavior of fine-tuning SleepFM on the SSC dataset. We progressively increased the proportion of labeled SSC data used during fine-tuning, from 10% to 100%. The plots show C-Index performance across multiple outcome categories. Notably, SleepFM achieves strong predictive performance even with just 10% of the training data, with performance improving consistently as more labeled data is used. Here, 100% corresponds to 6,000 SSC examples—substantially fewer than the full fine-tuning dataset of 24,137 examples. Note that while the same SSC data was used during fine-tuning and seen by the SleepFM model during pretraining, the pretraining objective was entirely different and lacked disease supervision, minimizing potential bias.

Supplementary Table 1. F1 scores for SleepFM and PhysioEx models on DCSM and HMC in an external validation setting (no site-specific fine-tuning).

	**DCSM**	**HMC**
SeqSleepNet	0.62	0.64
Chambon2018	0.43	0.65
TinySleepNet	0.34	0.63
SleepFM	0.68	0.55

Supplementary Table 2. Sleep staging results (macro F1 score) when pretrained on different cohorts, finetuned and tested of SHHS.

**Pretraining data set**			
**SSC**	**MESA**	**MROS**	**Bioserenity**
0.65	0.66	0.66	0.65

Supplementary Table 3. Cross-dataset generalization results (macro F1 score) for sleep staging models trained on one cohort and tested on others. Diagonal values represent within-dataset performance.

**Pretrain + Finetune**	**MESA (test)**	**MrOS (test)**	**Stanford (test)**	**Avg. transfer**
**MESA**	0.64	0.55	0.32	0.44
**MrOS**	0.40	0.65	0.39	0.40
**Stanford**	0.30	0.38	0.54	0.34
**Avg. test reception**	0.35	0.47	0.36	–

Supplementary Table 4. Sleep staging results (macro F1 score) using multi-dataset pretraining and fine-tuning. All models were pretrained on MESA, MrOS, Stanford, and Bioserenity, and fine-tuned on MESA, MrOS, Stanford, and SHHS.

**Test dataset**	**MESA**	**MrOS**	**Stanford**
**Macro F1**	0.74	0.74	0.67

Phecode	Phenotype	Category	C-Index	AUROC (6 Years)	Prevalence (%)
290.11	Alzheimer's disease	mental disorders	0.91 (0.87, 0.94)	0.96 (0.94, 0.98)	1.18
332.0	Parkinson's disease	neurological	0.89 (0.85, 0.92)	0.93 (0.89, 0.96)	1.33
185.0	Cancer of prostate	neoplasms	0.89 (0.86, 0.91)	0.90 (0.87, 0.93)	1.97
290.1	Dementias	mental disorders	0.88 (0.85, 0.90)	0.91 (0.87, 0.94)	2.99
218.1	Uterine leiomyoma	neoplasms	0.87 (0.85, 0.89)	0.88 (0.85, 0.91)	1.68
428.4	Heart failure with preserved EF [Diastolic heart failure]	circulatory system	0.87 (0.85, 0.90)	0.90 (0.86, 0.93)	2.98
218.0	Benign neoplasm of uterus	neoplasms	0.87 (0.85, 0.89)	0.88 (0.84, 0.91)	1.71
250.25	Diabetes type 2 with peripheral circulatory disorders	endocrine/metabolic	0.87 (0.83, 0.91)	0.89 (0.85, 0.93)	1.07
174.0	Breast cancer	neoplasms	0.87 (0.83, 0.90)	0.90 (0.86, 0.93)	1.47
174.11	Malignant neoplasm of female breast	neoplasms	0.87 (0.83, 0.90)	0.89 (0.85, 0.93)	1.52
504.0	Other alveolar and parietoalveolar pneumonopathy	respiratory	0.85 (0.80, 0.89)	0.90 (0.84, 0.95)	1.1
290.0	Delirium dementia and amnestic and other cognitive disorders	mental disorders	0.85 (0.82, 0.87)	0.87 (0.84, 0.91)	4.78
401.21	Hypertensive heart disease	circulatory system	0.84 (0.82, 0.86)	0.88 (0.85, 0.91)	4.01
496.21	Obstructive chronic bronchitis	respiratory	0.83 (0.79, 0.87)	0.90 (0.86, 0.93)	1.38
276.42	Alkalosis	endocrine/metabolic	0.83 (0.77, 0.88)	0.87 (0.77, 0.93)	1.07
447.7	Aortic ectasia	circulatory system	0.83 (0.80, 0.86)	0.90 (0.87, 0.93)	2.36
172.22	Squamous cell carcinoma	neoplasms	0.83 (0.78, 0.87)	0.83 (0.78, 0.88)	1.52
474.2	Chronic tonsillitis and adenoiditis	respiratory	0.83 (0.81, 0.85)	0.82 (0.79, 0.84)	7.59
250.22	Type 2 diabetes with renal manifestations	endocrine/metabolic	0.83 (0.80, 0.85)	0.87 (0.84, 0.90)	3.16
172.11	Melanomas of skin	neoplasms	0.83 (0.77, 0.87)	0.83 (0.76, 0.90)	1.2
395.2	Nonrheumatic aortic valve disorders	circulatory system	0.83 (0.79, 0.86)	0.86 (0.82, 0.90)	2.95
741.3	Difficulty in walking	musculoskeletal	0.83 (0.79, 0.86)	0.89 (0.84, 0.93)	2.51
474.0	Acute and chronic tonsillitis	respiratory	0.82 (0.80, 0.84)	0.81 (0.79, 0.84)	7.86
440.9	Atherosclerosis of aorta	circulatory system	0.82 (0.79, 0.86)	0.86 (0.82, 0.89)	1.83
427.22	Atrial flutter	circulatory system	0.82 (0.78, 0.85)	0.88 (0.84, 0.92)	3.04
443.9	Peripheral vascular disease, unspecified	circulatory system	0.82 (0.79, 0.85)	0.85 (0.81, 0.89)	3.3
433.1	Occlusion and stenosis of precerebral arteries	circulatory system	0.82 (0.79, 0.84)	0.85 (0.81, 0.88)	3.51
426.23	Second degree AV block	circulatory system	0.82 (0.77, 0.86)	0.89 (0.85, 0.92)	1.14
290.2	Delirium due to conditions classified elsewhere	mental disorders	0.82 (0.76, 0.87)	0.81 (0.74, 0.88)	1.98
250.24	Type 2 diabetes with neurological manifestations	endocrine/metabolic	0.81 (0.77, 0.85)	0.86 (0.81, 0.90)	2.42
250.7	Diabetic retinopathy	endocrine/metabolic	0.81 (0.77, 0.85)	0.84 (0.79, 0.89)	1.79
274.2	Crystal arthropathies	endocrine/metabolic	0.81 (0.76, 0.85)	0.83 (0.75, 0.90)	1.16
433.21	Cerebral artery occlusion, with cerebral infarction	circulatory system	0.81 (0.78, 0.84)	0.85 (0.80, 0.89)	2.7
411.2	Myocardial infarction	circulatory system	0.81 (0.78, 0.84)	0.85 (0.82, 0.88)	5.53
362.27	Drusen (degenerative) of retina	sense organs	0.81 (0.77, 0.85)	0.83 (0.78, 0.88)	1.37
426.92	Cardiac defibrillator in situ	circulatory system	0.81 (0.75, 0.87)	0.85 (0.79, 0.89)	1.08
292.2	Mild cognitive impairment	mental disorders	0.81 (0.78, 0.84)	0.84 (0.80, 0.88)	3.29
440.2	Atherosclerosis of the extremities	circulatory system	0.81 (0.74, 0.86)	0.84 (0.75, 0.90)	1.21
526.3	Anomalies of jaw size/symmetry	digestive	0.81 (0.77, 0.84)	0.84 (0.80, 0.88)	2.66
433.2	Occlusion of cerebral arteries	circulatory system	0.81 (0.77, 0.84)	0.85 (0.80, 0.89)	2.81
362.2	Degeneration of macula and posterior pole of retina	sense organs	0.81 (0.78, 0.83)	0.84 (0.81, 0.87)	4.34
411.8	Other chronic ischemic heart disease, unspecified	circulatory system	0.81 (0.76, 0.85)	0.81 (0.76, 0.86)	1.64
707.2	Chronic ulcer of leg or foot	dermatologic	0.80 (0.76, 0.85)	0.84 (0.79, 0.88)	1.12
428.3	Heart failure with reduced EF [Systolic or combined heart failure]	circulatory system	0.80 (0.77, 0.84)	0.83 (0.78, 0.87)	3.7
430.0	Intracranial hemorrhage	circulatory system	0.80 (0.74, 0.86)	0.82 (0.73, 0.90)	1.49
315.0	Develomental delays and disorders	mental disorders	0.80 (0.77, 0.84)	0.84 (0.79, 0.87)	2.65
415.1	Acute pulmonary heart disease	circulatory system	0.80 (0.75, 0.85)	0.84 (0.77, 0.90)	1.73
172.1	Melanomas of skin, dx or hx	neoplasms	0.80 (0.74, 0.86)	0.79 (0.69, 0.88)	1.34
415.11	Pulmonary embolism and infarction, acute	circulatory system	0.80 (0.75, 0.85)	0.85 (0.78, 0.91)	1.71
440.0	Atherosclerosis	circulatory system	0.80 (0.77, 0.83)	0.84 (0.80, 0.87)	3.66
401.2	Hypertensive heart and/or renal disease	circulatory system	0.80 (0.77, 0.82)	0.83 (0.80, 0.86)	7.58
428.0	Congestive heart failure; nonhypertensive	circulatory system	0.80 (0.77, 0.82)	0.83 (0.79, 0.85)	6.32
740.2	Osteoarthrosis, generalized	musculoskeletal	0.80 (0.76, 0.83)	0.85 (0.80, 0.89)	2.09
426.2	Atrioventricular [AV] block	circulatory system	0.79 (0.76, 0.83)	0.84 (0.80, 0.88)	4.26
374.6	Dermatochalasis	sense organs	0.79 (0.75, 0.83)	0.85 (0.80, 0.90)	2.21
249.0	Secondary diabetes mellitus	endocrine/metabolic	0.79 (0.74, 0.84)	0.82 (0.76, 0.88)	2.63
509.2	Respiratory insufficiency	respiratory	0.79 (0.72, 0.85)	0.82 (0.72, 0.91)	1.23
743.1	Osteoporosis	musculoskeletal	0.79 (0.77, 0.82)	0.81 (0.78, 0.85)	6
411.4	Coronary atherosclerosis	circulatory system	0.79 (0.77, 0.81)	0.83 (0.81, 0.86)	10.3
743.11	Osteoporosis NOS	musculoskeletal	0.79 (0.77, 0.82)	0.82 (0.78, 0.85)	6.24
426.32	Left bundle branch block	circulatory system	0.79 (0.76, 0.83)	0.82 (0.77, 0.87)	3.91
172.3	Carcinoma in situ of skin	neoplasms	0.79 (0.73, 0.84)	0.83 (0.76, 0.89)	1.01
285.21	Anemia in chronic kidney disease	hematopoietic	0.79 (0.74, 0.83)	0.78 (0.70, 0.85)	1.89
427.11	Paroxysmal supraventricular tachycardia	circulatory system	0.79 (0.76, 0.81)	0.87 (0.84, 0.90)	7.57
362.26	Macular puckering of retina	sense organs	0.79 (0.76, 0.82)	0.83 (0.79, 0.87)	2.4
275.6	Hypercalcemia	endocrine/metabolic	0.79 (0.73, 0.84)	0.83 (0.74, 0.91)	1.59
550.4	Umbilical hernia	digestive	0.79 (0.74, 0.83)	0.84 (0.78, 0.90)	1.25
365.1	Open-angle glaucoma	sense organs	0.78 (0.73, 0.83)	0.83 (0.77, 0.88)	1.16
427.1	Paroxysmal tachycardia, unspecified	circulatory system	0.78 (0.76, 0.81)	0.87 (0.84, 0.89)	8.33
350.2	Abnormality of gait	neurological	0.78 (0.76, 0.81)	0.83 (0.80, 0.86)	7.69
427.21	Atrial fibrillation	circulatory system	0.78 (0.75, 0.81)	0.81 (0.77, 0.84)	6.29
426.21	First degree AV block	circulatory system	0.78 (0.74, 0.82)	0.83 (0.78, 0.87)	3.45
427.8	Sinoatrial node dysfunction (Bradycardia)	circulatory system	0.78 (0.73, 0.83)	0.77 (0.70, 0.84)	2.23
411.1	Unstable angina (intermediate coronary syndrome)	circulatory system	0.78 (0.74, 0.82)	0.80 (0.75, 0.85)	1.66
285.1	Acute posthemorrhagic anemia	hematopoietic	0.78 (0.75, 0.81)	0.83 (0.79, 0.87)	4.57
427.2	Atrial fibrillation and flutter	circulatory system	0.78 (0.75, 0.81)	0.81 (0.78, 0.84)	6.38
427.12	Paroxysmal ventricular tachycardia	circulatory system	0.78 (0.74, 0.82)	0.84 (0.79, 0.88)	3.54
428.1	Congestive heart failure (CHF) NOS	circulatory system	0.78 (0.74, 0.81)	0.80 (0.76, 0.84)	5.06
442.1	Aortic aneurysm	circulatory system	0.78 (0.72, 0.83)	0.82 (0.74, 0.89)	1.55
366.2	Senile cataract	sense organs	0.78 (0.75, 0.80)	0.82 (0.79, 0.85)	9.07
427.61	Supraventricular premature beats	circulatory system	0.77 (0.75, 0.80)	0.83 (0.80, 0.86)	6.95
394.1	Mitral valve stenosis and aortic valve stenosis	circulatory system	0.77 (0.72, 0.83)	0.78 (0.71, 0.85)	1.35
411.0	Ischemic Heart Disease	circulatory system	0.77 (0.75, 0.79)	0.81 (0.78, 0.84)	12.22

Supplementary Table 5. Summary of SleepFM performance metrics, including C-Index and AUROC (6 years) on the Stanford dataset. Results are sorted by C-Index, ensuring that both AUROC and C-Index exceed 0.75, with 95% confidence intervals that do not overlap with 0.5 (random chance). All conditions have statistically significant p-values (<0.01) after Bonferroni correction. Notable top conditions include Alzheimer's disease, Parkinson's disease, and dementias.

Disease	Phecode	Phenotype
Dementia	290.0	Delirium dementia and amnestic and other cognitive disorders
Dementia	290.1	Dementias
Dementia	290.16	Vascular dementia
Dementia	290.12	Dementia with cerebral degenerations
Dementia	290.11	Alzheimer’s disease
Dementia	290.13	Senile dementia
Myocardial Infarction	411.0	Ischemic Heart Disease
Myocardial Infarction	411.2	Myocardial infarction
Ischemic Heart Disease	411.0	Ischemic Heart Disease
Ischemic Heart Disease	411.8	Other chronic ischemic heart disease, unspecified
Ischemic Heart Disease	411.9	Other acute and subacute forms of ischemic heart disease
Angina	411.1	Unstable angina (intermediate coronary syndrome)
Angina	411.3	Angina pectoris
Hypertension	401.0	Hypertension
Hypertension	401.1	Essential hypertension
Hypertension	401.2	Hypertensive heart and/or renal disease
Hypertension	401.21	Hypertensive heart disease
Hypertension	401.3	Other hypertensive complications
Hypertension	401.22	Hypertensive chronic kidney disease
Hypotension	458.0	Hypotension
Hypotension	458.1	Orthostatic hypotension
Hypotension	458.2	Iatrogenic hypotension
Hypotension	458.9	Hypotension NOS
Pulmonary Heart Disease	415.0	Pulmonary heart disease
Pulmonary Heart Disease	415.2	Chronic pulmonary heart disease
Pulmonary Heart Disease	415.1	Acute pulmonary heart disease
Pulmonary Heart Disease	415.21	Primary pulmonary hypertension
Pulmonary Heart Disease	415.11	Pulmonary embolism and infarction, acute
Atrial Fibrillation and Flutter	427.0	Cardiac dysrhythmias
Atrial Fibrillation and Flutter	427.2	Atrial fibrillation and flutter
Atrial Fibrillation and Flutter	427.21	Atrial fibrillation
Atrial Fibrillation and Flutter	427.22	Atrial flutter
Respiratory Failure	509.0	Respiratory failure, insufficiency, arrest
Respiratory Failure	509.1	Respiratory failure
Respiratory Failure	509.2	Respiratory insufficiency
Respiratory Failure	509.3	Pulmonary insufficiency or respiratory failure following trauma and surgery
Respiratory Failure	509.5	Respiratory arrest
Respiratory Failure	509.8	Dependence on respirator [Ventilator] or supplemental oxygen

Supplementary Table 6. Hand-selected diseases and their corresponding phecode groupings. These diseases were chosen for their relevance to sleep, in consultation with a sleep expert. All phecode groupings were reviewed and curated by a medical professional.

Phecode	Phenotype	Category	Wake	Stage1/2	Stage3	REM	Best
290.12	Dementia With Cerebral Degenerations	Mental Disorders	0.88 (0.82, 0.93)	0.87 (0.78, 0.93)	0.80 (0.68, 0.90)	0.82 (0.69, 0.91)	Wake
440.1	Atherosclerosis Of Renal Artery	Circulatory System	0.78 (0.65, 0.88)	0.76 (0.53, 0.94)	0.73 (0.50, 0.89)	0.74 (0.55, 0.90)	Wake
427.22	Atrial Flutter	Circulatory System	0.77 (0.73, 0.81)	0.77 (0.72, 0.81)	0.75 (0.70, 0.79)	0.75 (0.72, 0.79)	Wake
290.1	Dementias	Mental Disorders	0.77 (0.73, 0.80)	0.75 (0.71, 0.79)	0.75 (0.71, 0.79)	0.75 (0.71, 0.80)	Wake
306.1	Mental Disorders Durring/After Pregnancy	Mental Disorders	0.77 (0.68, 0.86)	0.72 (0.53, 0.86)	0.70 (0.51, 0.87)	0.67 (0.48, 0.83)	Wake
474.0	Acute And Chronic Tonsillitis	Respiratory	0.76 (0.74, 0.79)	0.76 (0.73, 0.79)	0.75 (0.72, 0.78)	0.76 (0.74, 0.79)	Wake
440.21	Atherosclerosis Of Native Arteries Of The Extremities With Ulceration Or Gangrene	Circulatory System	0.82 (0.63, 0.95)	0.95 (0.89, 1.00)	0.83 (0.74, 0.93)	0.83 (0.71, 0.95)	Stage 1/2
290.13	Senile Dementia	Mental Disorders	0.90 (0.85, 0.97)	0.94 (0.89, 0.99)	0.66 (0.39, 1.00)	0.89 (0.85, 0.94)	Stage 1/2
264.9	Lack Of Normal Physiological Development, Unspecified	Endocrine/Metabolic	0.87 (0.78, 0.95)	0.91 (0.84, 0.96)	0.91 (0.84, 0.96)	0.86 (0.77, 0.94)	Stage 1/2
313.3	Autism	Mental Disorders	0.88 (0.82, 0.93)	0.88 (0.83, 0.93)	0.88 (0.81, 0.93)	0.87 (0.79, 0.92)	Stage 1/2
411.8	Other Chronic Ischemic Heart Disease, Unspecified	Circulatory System	0.77 (0.73, 0.82)	0.83 (0.79, 0.86)	0.73 (0.69, 0.77)	0.78 (0.72, 0.82)	Stage 1/2
426.24	Atrioventricular Block, Complete	Circulatory System	0.82 (0.76, 0.87)	0.82 (0.75, 0.89)	0.80 (0.74, 0.87)	0.82 (0.76, 0.87)	Stage 1/2
250.25	Diabetes Type 2 With Peripheral Circulatory Disorders	Endocrine/Metabolic	0.81 (0.76, 0.87)	0.82 (0.76, 0.88)	0.75 (0.69, 0.80)	0.81 (0.76, 0.86)	Stage 1/2
496.21	Obstructive Chronic Bronchitis	Respiratory	0.74 (0.68, 0.79)	0.82 (0.78, 0.86)	0.71 (0.65, 0.76)	0.78 (0.73, 0.83)	Stage 1/2
264.0	Lack Of Normal Physiological Development	Endocrine/Metabolic	0.78 (0.68, 0.87)	0.81 (0.72, 0.88)	0.78 (0.67, 0.87)	0.79 (0.70, 0.87)	Stage 1/2
426.92	Cardiac Defibrillator In Situ	Circulatory System	0.76 (0.68, 0.82)	0.79 (0.72, 0.87)	0.79 (0.74, 0.85)	0.74 (0.67, 0.81)	Stage 1/2
276.42	Alkalosis	Endocrine/Metabolic	0.74 (0.65, 0.82)	0.79 (0.71, 0.85)	0.72 (0.63, 0.80)	0.77 (0.70, 0.83)	Stage 1/2
250.22	Type 2 Diabetes With Renal Manifestations	Endocrine/Metabolic	0.72 (0.68, 0.76)	0.79 (0.75, 0.82)	0.75 (0.70, 0.79)	0.77 (0.73, 0.81)	Stage 1/2
331.0	Other Cerebral Degenerations	Neurological	0.74 (0.65, 0.81)	0.79 (0.72, 0.85)	0.75 (0.67, 0.81)	0.74 (0.67, 0.80)	Stage 1/2
427.4	Cardiac Arrest And Ventricular Fibrillation	Circulatory System	0.69 (0.60, 0.77)	0.78 (0.70, 0.85)	0.68 (0.58, 0.78)	0.66 (0.58, 0.75)	Stage 1/2
256.4	Polycystic Ovaries	Endocrine/Metabolic	0.76 (0.66, 0.84)	0.78 (0.70, 0.85)	0.73 (0.66, 0.79)	0.63 (0.54, 0.73)	Stage 1/2
250.1	Type 1 Diabetes	Endocrine/Metabolic	0.69 (0.61, 0.76)	0.78 (0.72, 0.83)	0.64 (0.56, 0.71)	0.71 (0.65, 0.77)	Stage 1/2
440.9	Atherosclerosis Of Aorta	Circulatory System	0.73 (0.68, 0.79)	0.77 (0.72, 0.81)	0.69 (0.63, 0.75)	0.75 (0.70, 0.80)	Stage 1/2
707.2	Chronic Ulcer Of Leg Or Foot	Dermatologic	0.72 (0.64, 0.79)	0.77 (0.71, 0.82)	0.69 (0.63, 0.75)	0.71 (0.63, 0.77)	Stage 1/2
707.1	Decubitus Ulcer	Dermatologic	0.74 (0.67, 0.81)	0.77 (0.72, 0.82)	0.69 (0.61, 0.76)	0.75 (0.68, 0.81)	Stage 1/2
256.0	Ovarian Dysfunction	Endocrine/Metabolic	0.74 (0.65, 0.81)	0.77 (0.69, 0.84)	0.71 (0.64, 0.77)	0.63 (0.54, 0.71)	Stage 1/2
287.32	Secondary Thrombocytopenia	Hematopoietic	0.75 (0.67, 0.82)	0.77 (0.68, 0.84)	0.68 (0.57, 0.77)	0.72 (0.62, 0.80)	Stage 1/2
426.9	Cardiac Pacemaker/Device In Situ	Circulatory System	0.72 (0.68, 0.76)	0.76 (0.72, 0.81)	0.75 (0.71, 0.80)	0.71 (0.67, 0.75)	Stage 1/2
426.21	First Degree Av Block	Circulatory System	0.75 (0.71, 0.79)	0.76 (0.72, 0.80)	0.69 (0.64, 0.74)	0.74 (0.69, 0.79)	Stage 1/2
428.3	Heart Failure With Reduced Ef [Systolic Or Combined Heart Failure]	Circulatory System	0.74 (0.71, 0.78)	0.76 (0.73, 0.80)	0.71 (0.67, 0.75)	0.73 (0.70, 0.77)	Stage 1/2
430.0	Intracranial Hemorrhage	Circulatory System	0.73 (0.65, 0.80)	0.76 (0.70, 0.81)	0.70 (0.63, 0.77)	0.74 (0.67, 0.81)	Stage 1/2
433.8	Late Effects Of Cerebrovascular Disease	Circulatory System	0.74 (0.66, 0.80)	0.76 (0.69, 0.81)	0.70 (0.62, 0.76)	0.69 (0.61, 0.76)	Stage 1/2
443.9	Peripheral Vascular Disease, Unspecified	Circulatory System	0.75 (0.71, 0.78)	0.76 (0.72, 0.80)	0.69 (0.65, 0.73)	0.76 (0.72, 0.79)	Stage 1/2
415.11	Pulmonary Embolism And Infarction, Acute	Circulatory System	0.72 (0.66, 0.77)	0.76 (0.70, 0.81)	0.74 (0.68, 0.79)	0.70 (0.63, 0.77)	Stage 1/2
250.24	Type 2 Diabetes With Neurological Manifestations	Endocrine/Metabolic	0.72 (0.66, 0.77)	0.76 (0.71, 0.81)	0.68 (0.63, 0.72)	0.75 (0.71, 0.79)	Stage 1/2
172.11	Melanomas Of Skin	Neoplasms	0.68 (0.60, 0.75)	0.76 (0.70, 0.82)	0.75 (0.67, 0.82)	0.70 (0.63, 0.77)	Stage 1/2
332.0	Parkinson'S Disease	Neurological	0.76 (0.69, 0.81)	0.76 (0.70, 0.81)	0.73 (0.67, 0.78)	0.75 (0.68, 0.82)	Stage 1/2
377.1	Optic Atrophy	Sense Organs	0.70 (0.64, 0.77)	0.76 (0.70, 0.82)	0.66 (0.59, 0.73)	0.69 (0.63, 0.77)	Stage 1/2
415.1	Acute Pulmonary Heart Disease	Circulatory System	0.72 (0.66, 0.77)	0.75 (0.69, 0.81)	0.74 (0.68, 0.80)	0.70 (0.63, 0.76)	Stage 1/2
440.0	Atherosclerosis	Circulatory System	0.72 (0.68, 0.75)	0.75 (0.71, 0.79)	0.69 (0.65, 0.73)	0.71 (0.67, 0.75)	Stage 1/2
428.0	Congestive Heart Failure; Nonhypertensive	Circulatory System	0.72 (0.69, 0.75)	0.75 (0.72, 0.78)	0.72 (0.69, 0.75)	0.73 (0.70, 0.76)	Stage 1/2
457.2	Encounter For Long-Term (Current) Use Of Antiplatelets/Antithrombotics	Circulatory System	0.71 (0.66, 0.76)	0.75 (0.72, 0.79)	0.71 (0.67, 0.76)	0.72 (0.67, 0.77)	Stage 1/2
426.32	Left Bundle Branch Block	Circulatory System	0.74 (0.71, 0.78)	0.75 (0.71, 0.79)	0.72 (0.68, 0.76)	0.74 (0.70, 0.78)	Stage 1/2
509.1	Respiratory Failure	Respiratory	0.70 (0.66, 0.74)	0.75 (0.71, 0.79)	0.68 (0.64, 0.73)	0.72 (0.67, 0.76)	Stage 1/2
509.2	Respiratory Insufficiency	Respiratory	0.71 (0.64, 0.77)	0.75 (0.70, 0.81)	0.71 (0.63, 0.78)	0.69 (0.61, 0.76)	Stage 1/2
426.91	Cardiac Pacemaker In Situ	Circulatory System	0.74 (0.69, 0.78)	0.78 (0.73, 0.82)	0.79 (0.74, 0.83)	0.73 (0.68, 0.77)	Stage 3
425.2	Secondary/Extrinsic Cardiomyopathies	Circulatory System	0.59 (0.49, 0.69)	0.60 (0.51, 0.68)	0.77 (0.66, 0.86)	0.58 (0.48, 0.69)	Stage 3
428.4	Heart Failure With Preserved Ef [Diastolic Heart Failure]	Circulatory System	0.80 (0.76, 0.83)	0.81 (0.78, 0.85)	0.76 (0.73, 0.80)	0.82 (0.78, 0.85)	REM
290.16	Vascular Dementia	Mental Disorders	0.80 (0.72, 0.87)	0.76 (0.67, 0.84)	0.77 (0.67, 0.86)	0.80 (0.71, 0.87)	REM
362.29	Macular Degeneration (Senile) Of Retina Nos	Sense Organs	0.77 (0.72, 0.83)	0.78 (0.72, 0.83)	0.70 (0.62, 0.77)	0.79 (0.74, 0.84)	REM
315.2	Speech And Language Disorder	Mental Disorders	0.77 (0.67, 0.85)	0.74 (0.65, 0.82)	0.77 (0.68, 0.84)	0.78 (0.68, 0.85)	REM
401.21	Hypertensive Heart Disease	Circulatory System	0.72 (0.68, 0.76)	0.74 (0.71, 0.78)	0.74 (0.70, 0.77)	0.77 (0.73, 0.80)	REM
411.9	Other Acute And Subacute Forms Of Ischemic Heart Disease	Circulatory System	0.71 (0.62, 0.79)	0.69 (0.59, 0.79)	0.67 (0.58, 0.77)	0.77 (0.69, 0.85)	REM
426.8	Other Cardiac Conduction Disorders	Circulatory System	0.76 (0.72, 0.79)	0.75 (0.71, 0.79)	0.69 (0.65, 0.73)	0.77 (0.74, 0.81)	REM
290.0	Delirium Dementia And Amnestic And Other Cognitive Disorders	Mental Disorders	0.75 (0.72, 0.79)	0.74 (0.70, 0.77)	0.73 (0.70, 0.76)	0.77 (0.73, 0.80)	REM
290.2	Delirium Due To Conditions Classified Elsewhere	Mental Disorders	0.75 (0.70, 0.80)	0.73 (0.67, 0.79)	0.69 (0.63, 0.75)	0.77 (0.72, 0.82)	REM
474.2	Chronic Tonsillitis And Adenoiditis	Respiratory	0.77 (0.74, 0.79)	0.77 (0.74, 0.79)	0.75 (0.72, 0.78)	0.77 (0.74, 0.79)	REM
250.7	Diabetic Retinopathy	Endocrine/Metabolic	0.74 (0.67, 0.79)	0.73 (0.68, 0.78)	0.67 (0.61, 0.73)	0.76 (0.71, 0.81)	REM
264.2	Failure To Thrive (Childhood)	Endocrine/Metabolic	0.74 (0.62, 0.86)	0.73 (0.56, 0.86)	0.66 (0.51, 0.83)	0.75 (0.66, 0.86)	REM
260.2	Severe Protein-Calorie Malnutrition	Endocrine/Metabolic	0.73 (0.67, 0.78)	0.70 (0.64, 0.75)	0.62 (0.55, 0.68)	0.75 (0.70, 0.80)	REM
333.1	Essential Tremor	Neurological	0.69 (0.63, 0.76)	0.70 (0.62, 0.77)	0.65 (0.58, 0.71)	0.75 (0.68, 0.82)	REM
504.0	Other Alveolar And Parietoalveolar Pneumonopathy	Respiratory	0.72 (0.65, 0.79)	0.73 (0.65, 0.80)	0.75 (0.67, 0.83)	0.75 (0.67, 0.83)	REM

Supplementary Table 7. Performance comparison of models trained on different sleep stages across conditions. For each sleep stage, C-Index values are reported with 95% confidence intervals in parentheses. Only conditions where at least one sleep stage achieves a C-Index above 0.75 are included. Results are grouped by disease category and sorted alphabetically within each category. Stage 1/2 demonstrates the highest predictive performance across 39 conditions, followed by REM sleep with 15 conditions.

Phecode	Phenotype	Category	BAS	Respiratory	EKG	EMG	Best
313.3	Autism	Mental Disorders	0.89 (0.85, 0.93)	0.84 (0.77, 0.90)	0.86 (0.80, 0.91)	0.85 (0.80, 0.91)	BAS
290.12	Dementia With Cerebral Degenerations	Mental Disorders	0.88 (0.78, 0.95)	0.82 (0.75, 0.89)	0.83 (0.73, 0.91)	0.76 (0.67, 0.84)	BAS
290.16	Vascular Dementia	Mental Disorders	0.87 (0.82, 0.92)	0.78 (0.68, 0.87)	0.83 (0.75, 0.89)	0.78 (0.70, 0.85)	BAS
331.0	Other Cerebral Degenerations	Neurological	0.85 (0.80, 0.90)	0.74 (0.64, 0.82)	0.71 (0.62, 0.77)	0.74 (0.65, 0.82)	BAS
447.7	Aortic Ectasia	Circulatory System	0.82 (0.79, 0.85)	0.81 (0.78, 0.85)	0.80 (0.76, 0.84)	0.82 (0.78, 0.85)	BAS
250.7	Diabetic Retinopathy	Endocrine/Metabolic	0.82 (0.77, 0.86)	0.78 (0.74, 0.83)	0.80 (0.76, 0.84)	0.75 (0.69, 0.80)	BAS
264.0	Lack Of Normal Physiological Development	Endocrine/Metabolic	0.82 (0.74, 0.89)	0.77 (0.68, 0.85)	0.75 (0.65, 0.85)	0.80 (0.72, 0.87)	BAS
332.0	Parkinson'S Disease	Neurological	0.82 (0.76, 0.87)	0.82 (0.77, 0.86)	0.79 (0.73, 0.84)	0.78 (0.72, 0.83)	BAS
250.22	Type 2 Diabetes With Renal Manifestations	Endocrine/Metabolic	0.81 (0.78, 0.84)	0.78 (0.75, 0.81)	0.81 (0.78, 0.84)	0.77 (0.73, 0.80)	BAS
290.1	Dementias	Mental Disorders	0.81 (0.77, 0.84)	0.80 (0.76, 0.83)	0.78 (0.74, 0.81)	0.78 (0.74, 0.81)	BAS
362.29	Macular Degeneration (Senile) Of Retina Nos	Sense Organs	0.81 (0.76, 0.86)	0.74 (0.68, 0.80)	0.69 (0.61, 0.77)	0.75 (0.69, 0.81)	BAS
707.1	Decubitus Ulcer	Dermatologic	0.80 (0.74, 0.86)	0.71 (0.63, 0.79)	0.74 (0.67, 0.81)	0.78 (0.72, 0.83)	BAS
290.0	Delirium Dementia And Amnestic And Other Cognitive Disorders	Mental Disorders	0.80 (0.76, 0.83)	0.79 (0.75, 0.81)	0.78 (0.75, 0.81)	0.76 (0.72, 0.78)	BAS
504.0	Other Alveolar And Parietoalveolar Pneumonopathy	Respiratory	0.80 (0.73, 0.87)	0.80 (0.72, 0.87)	0.77 (0.70, 0.84)	0.79 (0.73, 0.86)	BAS
290.11	Alzheimer'S Disease	Mental Disorders	0.79 (0.74, 0.84)	0.76 (0.69, 0.82)	0.72 (0.66, 0.79)	0.74 (0.68, 0.79)	BAS
433.21	Cerebral Artery Occlusion, With Cerebral Infarction	Circulatory System	0.78 (0.74, 0.82)	0.75 (0.70, 0.79)	0.75 (0.71, 0.79)	0.77 (0.73, 0.81)	BAS
433.2	Occlusion Of Cerebral Arteries	Circulatory System	0.78 (0.73, 0.82)	0.74 (0.70, 0.79)	0.75 (0.70, 0.79)	0.77 (0.73, 0.81)	BAS
443.9	Peripheral Vascular Disease, Unspecified	Circulatory System	0.78 (0.74, 0.82)	0.75 (0.71, 0.79)	0.77 (0.73, 0.80)	0.75 (0.71, 0.79)	BAS
250.23	Type 2 Diabetes With Ophthalmic Manifestations	Endocrine/Metabolic	0.78 (0.71, 0.83)	0.74 (0.68, 0.79)	0.76 (0.71, 0.82)	0.71 (0.65, 0.77)	BAS
315.0	Develomental Delays And Disorders	Mental Disorders	0.78 (0.73, 0.82)	0.77 (0.73, 0.81)	0.77 (0.73, 0.81)	0.72 (0.67, 0.77)	BAS
333.2	Myoclonus	Neurological	0.78 (0.70, 0.85)	0.62 (0.54, 0.70)	0.68 (0.58, 0.77)	0.69 (0.60, 0.78)	BAS
433.1	Occlusion And Stenosis Of Precerebral Arteries	Circulatory System	0.77 (0.73, 0.80)	0.74 (0.70, 0.78)	0.73 (0.69, 0.77)	0.75 (0.71, 0.79)	BAS
264.2	Failure To Thrive (Childhood)	Endocrine/Metabolic	0.77 (0.67, 0.86)	0.67 (0.51, 0.83)	0.62 (0.46, 0.80)	0.76 (0.63, 0.86)	BAS
292.2	Mild Cognitive Impairment	Mental Disorders	0.77 (0.73, 0.81)	0.73 (0.68, 0.77)	0.73 (0.68, 0.77)	0.73 (0.69, 0.76)	BAS
202.0	Cancer Of Other Lymphoid, Histiocytic Tissue	Neoplasms	0.77 (0.70, 0.84)	0.75 (0.65, 0.83)	0.75 (0.66, 0.83)	0.73 (0.65, 0.81)	BAS
430.0	Intracranial Hemorrhage	Circulatory System	0.76 (0.70, 0.82)	0.69 (0.63, 0.76)	0.71 (0.64, 0.78)	0.70 (0.64, 0.76)	BAS
433.8	Late Effects Of Cerebrovascular Disease	Circulatory System	0.76 (0.70, 0.81)	0.69 (0.63, 0.76)	0.72 (0.65, 0.79)	0.70 (0.64, 0.75)	BAS
260.6	Anorexia	Endocrine/Metabolic	0.76 (0.72, 0.80)	0.72 (0.67, 0.77)	0.70 (0.64, 0.75)	0.73 (0.68, 0.77)	BAS
202.2	Non-Hodgkins Lymphoma	Neoplasms	0.76 (0.67, 0.84)	0.74 (0.62, 0.84)	0.72 (0.62, 0.82)	0.71 (0.62, 0.79)	BAS
350.2	Abnormality Of Gait	Neurological	0.76 (0.73, 0.78)	0.73 (0.70, 0.76)	0.73 (0.70, 0.76)	0.73 (0.70, 0.76)	BAS
362.26	Macular Puckering Of Retina	Sense Organs	0.76 (0.71, 0.79)	0.73 (0.68, 0.78)	0.71 (0.67, 0.76)	0.73 (0.69, 0.78)	BAS
362.2	Degeneration Of Macula And Posterior Pole Of Retina	Sense Organs	0.75 (0.72, 0.78)	0.73 (0.69, 0.76)	0.70 (0.67, 0.73)	0.72 (0.69, 0.75)	BAS
290.13	Senile Dementia	Mental Disorders	0.88 (0.78, 1.00)	0.92 (0.86, 0.99)	0.89 (0.87, 0.91)	0.67 (0.54, 0.83)	RESP
428.4	Heart Failure With Preserved Ef [Diastolic Heart Failure]	Circulatory System	0.83 (0.79, 0.86)	0.85 (0.82, 0.87)	0.84 (0.80, 0.87)	0.81 (0.78, 0.84)	RESP
401.21	Hypertensive Heart Disease	Circulatory System	0.81 (0.78, 0.84)	0.83 (0.80, 0.86)	0.82 (0.79, 0.85)	0.79 (0.76, 0.82)	RESP
440.1	Atherosclerosis Of Renal Artery	Circulatory System	0.73 (0.52, 0.94)	0.82 (0.69, 0.94)	0.81 (0.65, 0.93)	0.77 (0.62, 0.96)	RESP
496.21	Obstructive Chronic Bronchitis	Respiratory	0.76 (0.71, 0.81)	0.82 (0.77, 0.86)	0.80 (0.76, 0.85)	0.77 (0.71, 0.83)	RESP
426.23	Second Degree Av Block	Circulatory System	0.78 (0.73, 0.83)	0.81 (0.76, 0.84)	0.76 (0.70, 0.81)	0.79 (0.73, 0.83)	RESP
250.24	Type 2 Diabetes With Neurological Manifestations	Endocrine/Metabolic	0.78 (0.74, 0.83)	0.81 (0.76, 0.85)	0.80 (0.76, 0.83)	0.76 (0.71, 0.80)	RESP
741.3	Difficulty In Walking	Musculoskeletal	0.77 (0.72, 0.81)	0.81 (0.77, 0.84)	0.78 (0.73, 0.82)	0.76 (0.72, 0.81)	RESP
277.4	Disorders Of Bilirubin Excretion	Endocrine/Metabolic	0.79 (0.71, 0.85)	0.80 (0.72, 0.86)	0.70 (0.62, 0.77)	0.74 (0.65, 0.81)	RESP
276.11	Hyperosmolality And/Or Hypernatremia	Endocrine/Metabolic	0.74 (0.63, 0.83)	0.80 (0.72, 0.88)	0.78 (0.71, 0.85)	0.76 (0.66, 0.83)	RESP
315.2	Speech And Language Disorder	Mental Disorders	0.78 (0.69, 0.86)	0.80 (0.73, 0.86)	0.77 (0.69, 0.84)	0.74 (0.65, 0.82)	RESP
411.2	Myocardial Infarction	Circulatory System	0.78 (0.75, 0.81)	0.79 (0.76, 0.81)	0.78 (0.76, 0.81)	0.76 (0.73, 0.79)	RESP
250.1	Type 1 Diabetes	Endocrine/Metabolic	0.74 (0.67, 0.80)	0.78 (0.73, 0.83)	0.75 (0.69, 0.80)	0.71 (0.63, 0.77)	RESP
172.11	Melanomas Of Skin	Neoplasms	0.72 (0.65, 0.78)	0.78 (0.72, 0.83)	0.76 (0.69, 0.83)	0.71 (0.65, 0.77)	RESP
172.22	Squamous Cell Carcinoma	Neoplasms	0.71 (0.65, 0.77)	0.78 (0.73, 0.83)	0.75 (0.70, 0.80)	0.74 (0.68, 0.80)	RESP
427.11	Paroxysmal Supraventricular Tachycardia	Circulatory System	0.76 (0.73, 0.78)	0.77 (0.75, 0.80)	0.77 (0.74, 0.79)	0.76 (0.73, 0.79)	RESP
427.1	Paroxysmal Tachycardia, Unspecified	Circulatory System	0.76 (0.73, 0.78)	0.77 (0.75, 0.79)	0.77 (0.75, 0.79)	0.76 (0.73, 0.78)	RESP
505.0	Other Pulmonary Inflammation Or Edema	Respiratory	0.73 (0.65, 0.81)	0.77 (0.71, 0.83)	0.72 (0.65, 0.79)	0.70 (0.63, 0.77)	RESP
509.1	Respiratory Failure	Respiratory	0.72 (0.68, 0.76)	0.77 (0.72, 0.81)	0.76 (0.72, 0.80)	0.75 (0.71, 0.79)	RESP
509.2	Respiratory Insufficiency	Respiratory	0.69 (0.62, 0.77)	0.77 (0.69, 0.83)	0.76 (0.70, 0.82)	0.72 (0.64, 0.80)	RESP
415.1	Acute Pulmonary Heart Disease	Circulatory System	0.72 (0.67, 0.78)	0.76 (0.71, 0.82)	0.76 (0.71, 0.81)	0.73 (0.67, 0.78)	RESP
428.0	Congestive Heart Failure; Nonhypertensive	Circulatory System	0.74 (0.71, 0.77)	0.76 (0.73, 0.79)	0.76 (0.72, 0.79)	0.72 (0.69, 0.75)	RESP
415.11	Pulmonary Embolism And Infarction, Acute	Circulatory System	0.73 (0.67, 0.79)	0.76 (0.71, 0.81)	0.76 (0.71, 0.81)	0.73 (0.67, 0.78)	RESP
440.21	Atherosclerosis Of Native Arteries Of The Extremities With Ulceration Or Gangrene	Circulatory System	0.90 (0.82, 0.95)	0.86 (0.75, 0.94)	0.93 (0.87, 0.98)	0.86 (0.79, 0.92)	EKG
264.9	Lack Of Normal Physiological Development, Unspecified	Endocrine/Metabolic	0.86 (0.77, 0.93)	0.88 (0.82, 0.93)	0.90 (0.84, 0.95)	0.88 (0.81, 0.94)	EKG
426.24	Atrioventricular Block, Complete	Circulatory System	0.87 (0.80, 0.92)	0.85 (0.77, 0.91)	0.87 (0.82, 0.91)	0.81 (0.75, 0.88)	EKG
426.92	Cardiac Defibrillator In Situ	Circulatory System	0.79 (0.73, 0.84)	0.79 (0.73, 0.85)	0.87 (0.82, 0.91)	0.75 (0.69, 0.82)	EKG
250.25	Diabetes Type 2 With Peripheral Circulatory Disorders	Endocrine/Metabolic	0.83 (0.78, 0.88)	0.83 (0.80, 0.87)	0.86 (0.82, 0.89)	0.81 (0.75, 0.86)	EKG
411.8	Other Chronic Ischemic Heart Disease, Unspecified	Circulatory System	0.76 (0.70, 0.81)	0.77 (0.72, 0.82)	0.84 (0.80, 0.88)	0.75 (0.69, 0.80)	EKG
426.91	Cardiac Pacemaker In Situ	Circulatory System	0.77 (0.73, 0.81)	0.74 (0.69, 0.79)	0.82 (0.78, 0.86)	0.74 (0.69, 0.79)	EKG
276.42	Alkalosis	Endocrine/Metabolic	0.81 (0.74, 0.87)	0.81 (0.74, 0.87)	0.81 (0.74, 0.87)	0.77 (0.70, 0.84)	EKG
287.32	Secondary Thrombocytopenia	Hematopoietic	0.62 (0.52, 0.73)	0.77 (0.69, 0.84)	0.81 (0.73, 0.87)	0.69 (0.59, 0.77)	EKG
427.22	Atrial Flutter	Circulatory System	0.77 (0.73, 0.80)	0.80 (0.76, 0.83)	0.80 (0.77, 0.84)	0.74 (0.70, 0.78)	EKG
428.3	Heart Failure With Reduced Ef [Systolic Or Combined Heart Failure]	Circulatory System	0.75 (0.71, 0.79)	0.78 (0.75, 0.82)	0.80 (0.75, 0.84)	0.74 (0.70, 0.77)	EKG
426.8	Other Cardiac Conduction Disorders	Circulatory System	0.74 (0.70, 0.78)	0.80 (0.76, 0.83)	0.80 (0.77, 0.83)	0.72 (0.68, 0.76)	EKG
474.0	Acute And Chronic Tonsillitis	Respiratory	0.78 (0.75, 0.80)	0.77 (0.74, 0.79)	0.80 (0.78, 0.82)	0.74 (0.71, 0.77)	EKG
474.2	Chronic Tonsillitis And Adenoiditis	Respiratory	0.78 (0.76, 0.81)	0.78 (0.75, 0.80)	0.80 (0.78, 0.83)	0.75 (0.72, 0.77)	EKG
427.4	Cardiac Arrest And Ventricular Fibrillation	Circulatory System	0.66 (0.57, 0.74)	0.72 (0.63, 0.80)	0.79 (0.72, 0.85)	0.71 (0.64, 0.78)	EKG
426.9	Cardiac Pacemaker/Device In Situ	Circulatory System	0.74 (0.70, 0.78)	0.72 (0.68, 0.77)	0.79 (0.75, 0.83)	0.71 (0.66, 0.75)	EKG
249.0	Secondary Diabetes Mellitus	Endocrine/Metabolic	0.78 (0.73, 0.83)	0.78 (0.74, 0.82)	0.79 (0.75, 0.83)	0.75 (0.70, 0.79)	EKG
440.9	Atherosclerosis Of Aorta	Circulatory System	0.74 (0.70, 0.78)	0.76 (0.71, 0.81)	0.78 (0.73, 0.82)	0.70 (0.65, 0.74)	EKG
426.2	Atrioventricular [Av] Block	Circulatory System	0.72 (0.69, 0.76)	0.76 (0.72, 0.79)	0.78 (0.74, 0.81)	0.72 (0.67, 0.75)	EKG
428.2	Heart Failure Nos	Circulatory System	0.73 (0.64, 0.81)	0.74 (0.65, 0.82)	0.78 (0.70, 0.86)	0.69 (0.59, 0.77)	EKG
395.2	Nonrheumatic Aortic Valve Disorders	Circulatory System	0.77 (0.73, 0.81)	0.74 (0.70, 0.78)	0.78 (0.74, 0.81)	0.71 (0.66, 0.76)	EKG
427.8	Sinoatrial Node Dysfunction (Bradycardia)	Circulatory System	0.73 (0.67, 0.78)	0.78 (0.74, 0.82)	0.78 (0.74, 0.83)	0.70 (0.65, 0.75)	EKG
285.21	Anemia In Chronic Kidney Disease	Hematopoietic	0.76 (0.70, 0.81)	0.77 (0.72, 0.82)	0.78 (0.74, 0.82)	0.73 (0.67, 0.78)	EKG
426.21	First Degree Av Block	Circulatory System	0.70 (0.66, 0.74)	0.74 (0.70, 0.78)	0.77 (0.73, 0.81)	0.70 (0.66, 0.74)	EKG
401.2	Hypertensive Heart And/Or Renal Disease	Circulatory System	0.75 (0.72, 0.78)	0.77 (0.74, 0.79)	0.77 (0.74, 0.79)	0.73 (0.70, 0.75)	EKG
426.32	Left Bundle Branch Block	Circulatory System	0.72 (0.68, 0.75)	0.74 (0.70, 0.77)	0.77 (0.73, 0.81)	0.72 (0.68, 0.75)	EKG
395.3	Nonrheumatic Tricuspid Valve Disorders	Circulatory System	0.63 (0.56, 0.70)	0.75 (0.69, 0.80)	0.77 (0.72, 0.82)	0.65 (0.59, 0.71)	EKG
411.9	Other Acute And Subacute Forms Of Ischemic Heart Disease	Circulatory System	0.73 (0.64, 0.81)	0.76 (0.69, 0.83)	0.77 (0.70, 0.82)	0.70 (0.61, 0.78)	EKG
275.6	Hypercalcemia	Endocrine/Metabolic	0.75 (0.69, 0.80)	0.75 (0.69, 0.81)	0.77 (0.70, 0.82)	0.74 (0.66, 0.80)	EKG
427.12	Paroxysmal Ventricular Tachycardia	Circulatory System	0.75 (0.71, 0.79)	0.76 (0.71, 0.79)	0.76 (0.72, 0.80)	0.76 (0.71, 0.80)	EKG
571.51	Cirrhosis Of Liver Without Mention Of Alcohol	Digestive	0.67 (0.58, 0.76)	0.73 (0.65, 0.80)	0.76 (0.67, 0.82)	0.71 (0.62, 0.79)	EKG
440.2	Atherosclerosis Of The Extremities	Circulatory System	0.74 (0.68, 0.80)	0.72 (0.65, 0.79)	0.75 (0.68, 0.81)	0.68 (0.62, 0.74)	EKG
426.3	Bundle Branch Block	Circulatory System	0.71 (0.68, 0.74)	0.73 (0.69, 0.76)	0.75 (0.72, 0.78)	0.70 (0.67, 0.73)	EKG
420.3	Endocarditis	Circulatory System	0.66 (0.54, 0.77)	0.73 (0.63, 0.82)	0.75 (0.68, 0.82)	0.61 (0.50, 0.71)	EKG
427.6	Premature Beats	Circulatory System	0.73 (0.71, 0.75)	0.74 (0.71, 0.76)	0.75 (0.73, 0.77)	0.72 (0.70, 0.74)	EKG

Supplementary Table 8. Performance comparison of different modalities across conditions. For each modality, C-Index values are reported with 95% confidence intervals in parentheses. Only conditions where at least one modality achieves a C-Index above 0.75 are included. Results are grouped by disease category and sorted alphabetically within each category. BAS and EKG demonstrate the highest predictive performance, followed closely by RESP. EMG is the least predictive, with no top conditions where it performs best.

Category	BAS	EKG	EMG	RESP	SleepFM
circulatory system	0.71	0.73	0.69	0.72	0.75
dermatologic	0.63	0.63	0.63	0.63	0.66
digestive	0.63	0.65	0.64	0.65	0.67
endocrine/metabolic	0.67	0.68	0.66	0.68	0.71
hematopoietic	0.67	0.69	0.66	0.69	0.70
infectious diseases	0.63	0.63	0.63	0.64	0.66
injuries & poisonings	0.61	0.64	0.62	0.64	0.66
mental disorders	0.66	0.66	0.65	0.66	0.70
musculoskeletal	0.64	0.64	0.64	0.64	0.68
neoplasms	0.67	0.68	0.66	0.67	0.73
neurological	0.65	0.65	0.64	0.64	0.67
respiratory	0.62	0.64	0.62	0.64	0.66
sense organs	0.65	0.64	0.63	0.64	0.68
symptoms	0.66	0.66	0.66	0.66	0.68

Supplementary Table 9. Performance comparison between SleepFM trained on all modalities versus models trained on individual modalities alone. The table shows average C-Index across disease categories for each configuration. While certain modalities perform relatively well on their own, combining all modalities yields the highest performance, demonstrating the complementary nature of multimodal signals in PSG. These results support the benefit of leveraging multimodal data during training and highlight how different physiological signals contribute to disease prediction.

Category	BAS	EKG	EMG	RESP	SleepFM
circulatory system	0.75	0.77	0.74	0.76	0.79
dermatologic	0.69	0.69	0.69	0.69	0.70
digestive	0.69	0.70	0.70	0.70	0.72
endocrine/metabolic	0.72	0.72	0.71	0.72	0.75
hematopoietic	0.72	0.73	0.72	0.74	0.74
infectious diseases	0.68	0.67	0.68	0.68	0.70
injuries & poisonings	0.66	0.69	0.67	0.68	0.70
mental disorders	0.71	0.70	0.70	0.69	0.74
musculoskeletal	0.70	0.69	0.69	0.68	0.72
neoplasms	0.72	0.72	0.71	0.72	0.76
neurological	0.71	0.70	0.70	0.70	0.72
respiratory	0.67	0.68	0.67	0.68	0.70
sense organs	0.71	0.69	0.69	0.70	0.73
symptoms	0.73	0.73	0.73	0.73	0.75

Supplementary Table 10. Performance comparison between SleepFM trained on all modalities versus models trained on individual modalities alone. The table shows average AUROC across disease categories for each configuration. While certain modalities perform relatively well on their own, combining all modalities yields the highest performance, demonstrating the complementary nature of multimodal signals in PSG. These results support the benefit of leveraging multimodal data during training and highlight how different physiological signals contribute to disease prediction.

Phecode	Phenotype	Category	SleepFM	Demographics	End-to-End PSG
290.13	Senile dementia	mental disorders	0.99 (0.98, 1.00)	0.87 (0.75, 0.96)	0.95 (0.91, 0.98)
440.21	Atherosclerosis of native arteries of the extremities with ulceration or gangrene	circulatory system	0.92 (0.88, 0.95)	0.74 (0.64, 0.89)	0.66 (0.50, 0.89)
358.0	Myoneural disorders	neurological	0.81 (0.73, 0.88)	0.42 (0.28, 0.55)	0.60 (0.48, 0.71)
264.2	Failure to thrive (childhood)	endocrine/metabolic	0.77 (0.68, 0.88)	0.44 (0.26, 0.67)	0.45 (0.26, 0.67)
315.0	Develomental delays and disorders	mental disorders	0.80 (0.77, 0.84)	0.58 (0.51, 0.64)	0.61 (0.54, 0.67)
509.2	Respiratory insufficiency	respiratory	0.79 (0.72, 0.85)	0.59 (0.51, 0.67)	0.62 (0.53, 0.69)
277.4	Disorders of bilirubin excretion	endocrine/metabolic	0.79 (0.70, 0.85)	0.60 (0.46, 0.75)	0.61 (0.47, 0.75)
344.0	Other paralytic syndromes	neurological	0.77 (0.72, 0.83)	0.58 (0.48, 0.69)	0.60 (0.48, 0.69)
626.8	Infertility, female	genitourinary	0.89 (0.84, 0.93)	0.80 (0.73, 0.87)	0.88 (0.83, 0.92)
614.51	Cervicitis and endocervicitis	genitourinary	0.88 (0.85, 0.91)	0.79 (0.74, 0.83)	0.86 (0.83, 0.89)
619.2	Disorders of uterus, NEC	genitourinary	0.87 (0.84, 0.90)	0.79 (0.76, 0.82)	0.84 (0.81, 0.88)
609.0	Male infertility and abnormal spermatozoa	genitourinary	0.82 (0.78, 0.86)	0.71 (0.66, 0.76)	0.82 (0.78, 0.86)
504.0	Other alveolar and parietoalveolar pneumonopathy	respiratory	0.85 (0.80, 0.89)	0.75 (0.68, 0.81)	0.75 (0.68, 0.81)
619.3	Noninflammatory disorders of cervix	genitourinary	0.86 (0.82, 0.89)	0.77 (0.72, 0.81)	0.82 (0.79, 0.85)
250.25	Diabetes type 2 with peripheral circulatory disorders	endocrine/metabolic	0.87 (0.83, 0.91)	0.79 (0.74, 0.85)	0.78 (0.72, 0.84)
426.24	Atrioventricular block, complete	circulatory system	0.87 (0.83, 0.92)	0.80 (0.73, 0.87)	0.83 (0.76, 0.89)
348.8	Encephalopathy, not elsewhere classified	neurological	0.77 (0.70, 0.83)	0.63 (0.56, 0.71)	0.71 (0.64, 0.77)
276.42	Alkalosis	endocrine/metabolic	0.83 (0.77, 0.88)	0.74 (0.66, 0.80)	0.71 (0.63, 0.78)
249.0	Secondary diabetes mellitus	endocrine/metabolic	0.79 (0.74, 0.84)	0.68 (0.63, 0.72)	0.67 (0.62, 0.71)
447.7	Aortic ectasia	circulatory system	0.83 (0.80, 0.86)	0.74 (0.69, 0.79)	0.78 (0.74, 0.82)
280.2	Iron deficiency anemia secondary to blood loss (chronic)	hematopoietic	0.77 (0.73, 0.81)	0.64 (0.59, 0.69)	0.64 (0.60, 0.69)
218.1	Uterine leiomyoma	neoplasms	0.87 (0.85, 0.89)	0.81 (0.79, 0.83)	0.87 (0.84, 0.89)
300.9	Posttraumatic stress disorder	mental disorders	0.75 (0.70, 0.79)	0.62 (0.56, 0.68)	0.64 (0.59, 0.69)
331.0	Other cerebral degenerations	neurological	0.83 (0.76, 0.88)	0.74 (0.65, 0.82)	0.79 (0.70, 0.87)
290.2	Delirium due to conditions classified elsewhere	mental disorders	0.82 (0.76, 0.87)	0.73 (0.67, 0.78)	0.77 (0.71, 0.82)
218.0	Benign neoplasm of uterus	neoplasms	0.87 (0.85, 0.89)	0.81 (0.79, 0.83)	0.87 (0.85, 0.89)
276.4	Acid-base balance disorder	endocrine/metabolic	0.77 (0.72, 0.82)	0.66 (0.61, 0.72)	0.67 (0.61, 0.72)
250.24	Type 2 diabetes with neurological manifestations	endocrine/metabolic	0.81 (0.77, 0.85)	0.73 (0.68, 0.77)	0.71 (0.68, 0.75)
315.2	Speech and language disorder	mental disorders	0.81 (0.74, 0.87)	0.73 (0.63, 0.82)	0.73 (0.64, 0.82)
530.2	Esophageal bleeding (varices/hemorrhage)	digestive	0.76 (0.72, 0.79)	0.65 (0.60, 0.71)	0.69 (0.64, 0.74)
260.6	Anorexia	endocrine/metabolic	0.76 (0.71, 0.80)	0.65 (0.59, 0.72)	0.68 (0.62, 0.74)
458.2	Iatrogenic hypotension	circulatory system	0.76 (0.68, 0.83)	0.66 (0.58, 0.74)	0.67 (0.60, 0.74)
585.31	Renal dialysis	genitourinary	0.78 (0.73, 0.82)	0.68 (0.63, 0.74)	0.66 (0.60, 0.71)
426.8	Other cardiac conduction disorders	circulatory system	0.79 (0.75, 0.82)	0.70 (0.65, 0.74)	0.71 (0.66, 0.75)
276.11	Hyperosmolality and/or hypernatremia	endocrine/metabolic	0.76 (0.66, 0.86)	0.67 (0.58, 0.75)	0.68 (0.59, 0.76)
264.0	Lack of normal physiological development	endocrine/metabolic	0.77 (0.67, 0.86)	0.69 (0.56, 0.81)	0.69 (0.55, 0.81)
707.2	Chronic ulcer of leg or foot	dermatologic	0.80 (0.76, 0.85)	0.73 (0.68, 0.79)	0.70 (0.65, 0.75)
622.1	Polyp of corpus uteri	genitourinary	0.85 (0.81, 0.89)	0.79 (0.75, 0.83)	0.85 (0.81, 0.88)
594.3	Calculus of ureter	genitourinary	0.75 (0.69, 0.81)	0.67 (0.60, 0.73)	0.67 (0.62, 0.73)
250.7	Diabetic retinopathy	endocrine/metabolic	0.81 (0.77, 0.85)	0.75 (0.69, 0.80)	0.74 (0.69, 0.79)
513.8	Disorders of diaphragm	respiratory	0.75 (0.65, 0.84)	0.67 (0.56, 0.76)	0.68 (0.57, 0.77)
592.11	Acute cystitis	genitourinary	0.81 (0.78, 0.83)	0.74 (0.72, 0.77)	0.74 (0.71, 0.77)
285.1	Acute posthemorrhagic anemia	hematopoietic	0.78 (0.75, 0.81)	0.71 (0.67, 0.74)	0.72 (0.68, 0.75)
440.2	Atherosclerosis of the extremities	circulatory system	0.81 (0.74, 0.86)	0.75 (0.68, 0.80)	0.75 (0.69, 0.81)
362.27	Drusen (degenerative) of retina	sense organs	0.81 (0.77, 0.85)	0.75 (0.69, 0.80)	0.83 (0.79, 0.87)
501.0	Pneumonitis due to inhalation of food or vomitus	respiratory	0.75 (0.70, 0.80)	0.68 (0.62, 0.73)	0.69 (0.62, 0.76)
415.11	Pulmonary embolism and infarction, acute	circulatory system	0.80 (0.75, 0.85)	0.74 (0.68, 0.79)	0.75 (0.69, 0.81)
427.11	Paroxysmal supraventricular tachycardia	circulatory system	0.79 (0.76, 0.81)	0.72 (0.69, 0.76)	0.73 (0.70, 0.76)
415.1	Acute pulmonary heart disease	circulatory system	0.80 (0.75, 0.85)	0.74 (0.68, 0.80)	0.75 (0.69, 0.81)
411.8	Other chronic ischemic heart disease, unspecified	circulatory system	0.81 (0.76, 0.85)	0.75 (0.69, 0.79)	0.77 (0.72, 0.81)
440.22	Atherosclerosis of native arteries of the extremities with intermittent claudication	circulatory system	0.80 (0.73, 0.86)	0.75 (0.68, 0.83)	0.74 (0.65, 0.81)
411.9	Other acute and subacute forms of ischemic heart disease	circulatory system	0.78 (0.72, 0.85)	0.72 (0.64, 0.79)	0.72 (0.66, 0.79)
509.1	Respiratory failure	respiratory	0.77 (0.73, 0.80)	0.70 (0.65, 0.74)	0.69 (0.64, 0.73)
740.2	Osteoarthrosis, generalized	musculoskeletal	0.80 (0.76, 0.83)	0.74 (0.69, 0.79)	0.79 (0.74, 0.82)

Supplementary Table 11. Top conditions where sleep recordings provide substantial predictive value beyond demographic information alone. For each condition, we compare the predictive performance (C-Index) of SleepFM against demographic-only and end-to-end PSG models. The table highlights conditions where SleepFM achieved both strong absolute performance (C-Index > 0.75) and demonstrated meaningful improvement over demographic features (difference in C-Index > 0.5). Conditions are ranked by their relative improvement over demographics, emphasizing areas where sleep patterns contribute most significantly to prediction accuracy. Notable top conditions where SleepFM performs much better than baseline, include dementia, respiratory insufficiency, and developmental delays and disorders.

Phecode	Phenotype	Category	SleepFM	Demographics	End-to-End PSG
290.13	Senile dementia	mental disorders	0.99 (0.98, 1.00)	0.87 (0.75, 0.96)	0.95 (0.91, 0.98)
440.21	Atherosclerosis of native arteries of the extremities with ulceration or gangrene	circulatory system	0.92 (0.88, 0.95)	0.74 (0.64, 0.89)	0.66 (0.50, 0.89)
358.0	Myoneural disorders	neurological	0.81 (0.73, 0.88)	0.42 (0.28, 0.55)	0.60 (0.48, 0.71)
264.2	Failure to thrive (childhood)	endocrine/metabolic	0.77 (0.68, 0.88)	0.44 (0.26, 0.67)	0.45 (0.26, 0.67)
315.0	Develomental delays and disorders	mental disorders	0.80 (0.77, 0.84)	0.58 (0.51, 0.64)	0.61 (0.54, 0.67)
509.2	Respiratory insufficiency	respiratory	0.79 (0.72, 0.85)	0.59 (0.51, 0.67)	0.62 (0.53, 0.69)
277.4	Disorders of bilirubin excretion	endocrine/metabolic	0.79 (0.70, 0.85)	0.60 (0.46, 0.75)	0.61 (0.47, 0.75)
344.0	Other paralytic syndromes	neurological	0.77 (0.72, 0.83)	0.58 (0.48, 0.69)	0.60 (0.48, 0.69)
504.0	Other alveolar and parietoalveolar pneumonopathy	respiratory	0.85 (0.80, 0.89)	0.75 (0.68, 0.81)	0.75 (0.68, 0.81)
250.25	Diabetes type 2 with peripheral circulatory disorders	endocrine/metabolic	0.87 (0.83, 0.91)	0.79 (0.74, 0.85)	0.78 (0.72, 0.84)
426.24	Atrioventricular block, complete	circulatory system	0.87 (0.83, 0.92)	0.80 (0.73, 0.87)	0.83 (0.76, 0.89)
348.8	Encephalopathy, not elsewhere classified	neurological	0.77 (0.70, 0.83)	0.63 (0.56, 0.71)	0.71 (0.64, 0.77)
276.42	Alkalosis	endocrine/metabolic	0.83 (0.77, 0.88)	0.74 (0.66, 0.80)	0.71 (0.63, 0.78)
249.0	Secondary diabetes mellitus	endocrine/metabolic	0.79 (0.74, 0.84)	0.68 (0.63, 0.72)	0.67 (0.62, 0.71)
447.7	Aortic ectasia	circulatory system	0.83 (0.80, 0.86)	0.74 (0.69, 0.79)	0.78 (0.74, 0.82)
280.2	Iron deficiency anemia secondary to blood loss (chronic)	hematopoietic	0.77 (0.73, 0.81)	0.64 (0.59, 0.69)	0.64 (0.60, 0.69)
218.1	Uterine leiomyoma	neoplasms	0.87 (0.85, 0.89)	0.81 (0.79, 0.83)	0.87 (0.84, 0.89)
300.9	Posttraumatic stress disorder	mental disorders	0.75 (0.70, 0.79)	0.62 (0.56, 0.68)	0.64 (0.59, 0.69)
331.0	Other cerebral degenerations	neurological	0.83 (0.76, 0.88)	0.74 (0.65, 0.82)	0.79 (0.70, 0.87)
290.2	Delirium due to conditions classified elsewhere	mental disorders	0.82 (0.76, 0.87)	0.73 (0.67, 0.78)	0.77 (0.71, 0.82)
218.0	Benign neoplasm of uterus	neoplasms	0.87 (0.85, 0.89)	0.81 (0.79, 0.83)	0.87 (0.85, 0.89)
276.4	Acid-base balance disorder	endocrine/metabolic	0.77 (0.72, 0.82)	0.66 (0.61, 0.72)	0.67 (0.61, 0.72)
250.24	Type 2 diabetes with neurological manifestations	endocrine/metabolic	0.81 (0.77, 0.85)	0.73 (0.68, 0.77)	0.71 (0.68, 0.75)
315.2	Speech and language disorder	mental disorders	0.81 (0.74, 0.87)	0.73 (0.63, 0.82)	0.73 (0.64, 0.82)
530.2	Esophageal bleeding (varices/hemorrhage)	digestive	0.76 (0.72, 0.79)	0.65 (0.60, 0.71)	0.69 (0.64, 0.74)
260.6	Anorexia	endocrine/metabolic	0.76 (0.71, 0.80)	0.65 (0.59, 0.72)	0.68 (0.62, 0.74)
458.2	Iatrogenic hypotension	circulatory system	0.76 (0.68, 0.83)	0.66 (0.58, 0.74)	0.67 (0.60, 0.74)
426.8	Other cardiac conduction disorders	circulatory system	0.79 (0.75, 0.82)	0.70 (0.65, 0.74)	0.71 (0.66, 0.75)
276.11	Hyperosmolality and/or hypernatremia	endocrine/metabolic	0.76 (0.66, 0.86)	0.67 (0.58, 0.75)	0.68 (0.59, 0.76)
264.0	Lack of normal physiological development	endocrine/metabolic	0.77 (0.67, 0.86)	0.69 (0.56, 0.81)	0.69 (0.55, 0.81)
707.2	Chronic ulcer of leg or foot	dermatologic	0.80 (0.76, 0.85)	0.73 (0.68, 0.79)	0.70 (0.65, 0.75)
250.7	Diabetic retinopathy	endocrine/metabolic	0.81 (0.77, 0.85)	0.75 (0.69, 0.80)	0.74 (0.69, 0.79)
513.8	Disorders of diaphragm	respiratory	0.75 (0.65, 0.84)	0.67 (0.56, 0.76)	0.68 (0.57, 0.77)
285.1	Acute posthemorrhagic anemia	hematopoietic	0.78 (0.75, 0.81)	0.71 (0.67, 0.74)	0.72 (0.68, 0.75)
440.2	Atherosclerosis of the extremities	circulatory system	0.81 (0.74, 0.86)	0.75 (0.68, 0.80)	0.75 (0.69, 0.81)
362.27	Drusen (degenerative) of retina	sense organs	0.81 (0.77, 0.85)	0.75 (0.69, 0.80)	0.83 (0.79, 0.87)
501.0	Pneumonitis due to inhalation of food or vomitus	respiratory	0.75 (0.70, 0.80)	0.68 (0.62, 0.73)	0.69 (0.62, 0.76)
415.11	Pulmonary embolism and infarction, acute	circulatory system	0.80 (0.75, 0.85)	0.74 (0.68, 0.79)	0.75 (0.69, 0.81)
427.11	Paroxysmal supraventricular tachycardia	circulatory system	0.79 (0.76, 0.81)	0.72 (0.69, 0.76)	0.73 (0.70, 0.76)
415.1	Acute pulmonary heart disease	circulatory system	0.80 (0.75, 0.85)	0.74 (0.68, 0.80)	0.75 (0.69, 0.81)
411.8	Other chronic ischemic heart disease, unspecified	circulatory system	0.81 (0.76, 0.85)	0.75 (0.69, 0.79)	0.77 (0.72, 0.81)
440.22	Atherosclerosis of native arteries of the extremities with intermittent claudication	circulatory system	0.80 (0.73, 0.86)	0.75 (0.68, 0.83)	0.74 (0.65, 0.81)
411.9	Other acute and subacute forms of ischemic heart disease	circulatory system	0.78 (0.72, 0.85)	0.72 (0.64, 0.79)	0.72 (0.66, 0.79)
509.1	Respiratory failure	respiratory	0.77 (0.73, 0.80)	0.70 (0.65, 0.74)	0.69 (0.64, 0.73)
740.2	Osteoarthrosis, generalized	musculoskeletal	0.80 (0.76, 0.83)	0.74 (0.69, 0.79)	0.79 (0.74, 0.82)
427.1	Paroxysmal tachycardia, unspecified	circulatory system	0.78 (0.76, 0.81)	0.73 (0.70, 0.75)	0.73 (0.71, 0.76)
427.4	Cardiac arrest and ventricular fibrillation	circulatory system	0.77 (0.68, 0.84)	0.71 (0.63, 0.78)	0.73 (0.65, 0.81)
569.2	Gastrointestinal complications	digestive	0.76 (0.70, 0.81)	0.69 (0.62, 0.77)	0.66 (0.59, 0.72)
427.12	Paroxysmal ventricular tachycardia	circulatory system	0.78 (0.74, 0.82)	0.72 (0.68, 0.77)	0.72 (0.67, 0.77)
260.2	severe protein-calorie malnutrition	endocrine/metabolic	0.79 (0.74, 0.83)	0.73 (0.68, 0.78)	0.71 (0.67, 0.76)
285.2	Anemia of chronic disease	hematopoietic	0.76 (0.72, 0.79)	0.70 (0.66, 0.75)	0.71 (0.67, 0.75)
459.9	Circulatory disease NEC	circulatory system	0.77 (0.74, 0.79)	0.71 (0.68, 0.74)	0.71 (0.68, 0.75)
276.13	Hyperpotassemia	endocrine/metabolic	0.75 (0.71, 0.80)	0.70 (0.65, 0.75)	0.70 (0.65, 0.74)
574.2	Calculus of bile duct	digestive	0.76 (0.70, 0.80)	0.70 (0.63, 0.78)	0.70 (0.63, 0.78)
250.1	Type 1 diabetes	endocrine/metabolic	0.75 (0.69, 0.81)	0.70 (0.64, 0.75)	0.68 (0.62, 0.75)

Supplementary Table 12. Top conditions where sleep recordings demonstrate strong predictive performance for 6-year risk assessment. For each condition, we compare the 6-year AUROC of SleepFM against demographic-only and end-to-end PSG models. The table includes conditions where SleepFM achieved both strong absolute performance (AUROC > 0.75) and substantial improvement over the demographic baseline. Conditions are ranked by their relative improvement over demographics, highlighting diseases where sleep patterns provide the most significant additional predictive value. Notable top conditions where SleepFM performs much better than baseline, include dementia, respiratory insufficiency, and developmental delays and disorders.

Phecode	Phenotype	Category	C-Index		AUROC (6 Years)	
			SleepFM	Demographics	SleepFM	Demographics
290.13	Senile dementia	mental disorders	0.99 (0.98, 1.00)	0.87 (0.75, 0.96)	0.99 (0.98, 1.00)	0.85 (0.75, 0.97)
440.21	Atherosclerosis of native arteries of the extremities with ulceration or gangrene	circulatory system	0.92 (0.88, 0.95)	0.74 (0.64, 0.89)	0.95 (0.91, 0.98)	0.76 (0.72, 0.80)
358.0	Myoneural disorders	neurological	0.81 (0.73, 0.88)	0.42 (0.28, 0.55)	0.84 (0.75, 0.91)	0.38 (0.25, 0.53)
264.2	Failure to thrive (childhood)	endocrine/metabolic	0.77 (0.68, 0.88)	0.44 (0.26, 0.67)	0.75 (0.65, 0.87)	0.46 (0.29, 0.66)
315.0	Develomental delays and disorders	mental disorders	0.80 (0.77, 0.84)	0.58 (0.51, 0.64)	0.84 (0.79, 0.87)	0.58 (0.49, 0.66)
509.2	Respiratory insufficiency	respiratory	0.79 (0.72, 0.85)	0.59 (0.51, 0.67)	0.82 (0.72, 0.91)	0.57 (0.46, 0.67)
277.4	Disorders of bilirubin excretion	endocrine/metabolic	0.79 (0.70, 0.85)	0.60 (0.46, 0.75)	0.89 (0.84, 0.93)	0.62 (0.41, 0.80)
344.0	Other paralytic syndromes	neurological	0.77 (0.72, 0.83)	0.58 (0.48, 0.69)	0.82 (0.75, 0.88)	0.55 (0.41, 0.68)
504.0	Other alveolar and parietoalveolar pneumonopathy	respiratory	0.85 (0.80, 0.89)	0.75 (0.68, 0.81)	0.90 (0.84, 0.95)	0.76 (0.66, 0.84)
250.25	Diabetes type 2 with peripheral circulatory disorders	endocrine/metabolic	0.87 (0.83, 0.91)	0.79 (0.74, 0.85)	0.89 (0.85, 0.93)	0.79 (0.72, 0.87)
426.24	Atrioventricular block, complete	circulatory system	0.87 (0.83, 0.92)	0.80 (0.73, 0.87)	0.89 (0.84, 0.94)	0.80 (0.70, 0.88)
348.8	Encephalopathy, not elsewhere classified	neurological	0.77 (0.70, 0.83)	0.63 (0.56, 0.71)	0.78 (0.70, 0.86)	0.63 (0.54, 0.73)
276.42	Alkalosis	endocrine/metabolic	0.83 (0.77, 0.88)	0.74 (0.66, 0.80)	0.87 (0.77, 0.93)	0.75 (0.64, 0.84)
249.0	Secondary diabetes mellitus	endocrine/metabolic	0.79 (0.74, 0.84)	0.68 (0.63, 0.72)	0.82 (0.76, 0.88)	0.67 (0.59, 0.74)
447.7	Aortic ectasia	circulatory system	0.83 (0.80, 0.86)	0.74 (0.69, 0.79)	0.90 (0.87, 0.93)	0.74 (0.67, 0.80)
280.2	Iron deficiency anemia secondary to blood loss (chronic)	hematopoietic	0.77 (0.73, 0.81)	0.64 (0.59, 0.69)	0.84 (0.79, 0.89)	0.66 (0.59, 0.72)
218.1	Uterine leiomyoma	neoplasms	0.87 (0.85, 0.89)	0.81 (0.79, 0.83)	0.88 (0.85, 0.91)	0.80 (0.77, 0.83)
300.9	Posttraumatic stress disorder	mental disorders	0.75 (0.70, 0.79)	0.62 (0.56, 0.68)	0.82 (0.76, 0.87)	0.61 (0.54, 0.68)
331.0	Other cerebral degenerations	neurological	0.83 (0.76, 0.88)	0.74 (0.65, 0.82)	0.85 (0.77, 0.91)	0.75 (0.65, 0.84)
290.2	Delirium due to conditions classified elsewhere	mental disorders	0.82 (0.76, 0.87)	0.73 (0.67, 0.78)	0.81 (0.74, 0.88)	0.71 (0.64, 0.78)
218.0	Benign neoplasm of uterus	neoplasms	0.87 (0.85, 0.89)	0.81 (0.79, 0.83)	0.88 (0.84, 0.91)	0.80 (0.77, 0.83)
276.4	Acid-base balance disorder	endocrine/metabolic	0.77 (0.72, 0.82)	0.66 (0.61, 0.72)	0.79 (0.73, 0.85)	0.67 (0.59, 0.74)
250.24	Type 2 diabetes with neurological manifestations	endocrine/metabolic	0.81 (0.77, 0.85)	0.73 (0.68, 0.77)	0.86 (0.81, 0.90)	0.74 (0.68, 0.80)
315.2	Speech and language disorder	mental disorders	0.81 (0.74, 0.87)	0.73 (0.63, 0.82)	0.83 (0.74, 0.90)	0.72 (0.60, 0.83)
530.2	Esophageal bleeding (varices/hemorrhage)	digestive	0.76 (0.72, 0.79)	0.65 (0.60, 0.71)	0.87 (0.84, 0.90)	0.66 (0.57, 0.73)
260.6	Anorexia	endocrine/metabolic	0.76 (0.71, 0.80)	0.65 (0.59, 0.72)	0.84 (0.79, 0.88)	0.65 (0.55, 0.74)
458.2	Iatrogenic hypotension	circulatory system	0.76 (0.68, 0.83)	0.66 (0.58, 0.74)	0.77 (0.65, 0.87)	0.66 (0.53, 0.77)
426.8	Other cardiac conduction disorders	circulatory system	0.79 (0.75, 0.82)	0.70 (0.65, 0.74)	0.82 (0.77, 0.87)	0.69 (0.62, 0.75)
276.11	Hyperosmolality and/or hypernatremia	endocrine/metabolic	0.76 (0.66, 0.86)	0.67 (0.58, 0.75)	0.77 (0.59, 0.94)	0.78 (0.68, 0.88)
264.0	Lack of normal physiological development	endocrine/metabolic	0.77 (0.67, 0.86)	0.69 (0.56, 0.81)	0.72 (0.60, 0.84)	0.64 (0.49, 0.78)
707.2	Chronic ulcer of leg or foot	dermatologic	0.80 (0.76, 0.85)	0.73 (0.68, 0.79)	0.84 (0.79, 0.88)	0.75 (0.67, 0.81)
250.7	Diabetic retinopathy	endocrine/metabolic	0.81 (0.77, 0.85)	0.75 (0.69, 0.80)	0.84 (0.79, 0.89)	0.77 (0.70, 0.82)
513.8	Disorders of diaphragm	respiratory	0.75 (0.65, 0.84)	0.67 (0.56, 0.76)	0.73 (0.58, 0.86)	0.63 (0.48, 0.77)
285.1	Acute posthemorrhagic anemia	hematopoietic	0.78 (0.75, 0.81)	0.71 (0.67, 0.74)	0.83 (0.79, 0.87)	0.71 (0.66, 0.77)
440.2	Atherosclerosis of the extremities	circulatory system	0.81 (0.74, 0.86)	0.75 (0.68, 0.80)	0.84 (0.75, 0.90)	0.77 (0.68, 0.84)
362.27	Drusen (degenerative) of retina	sense organs	0.81 (0.77, 0.85)	0.75 (0.69, 0.80)	0.83 (0.78, 0.88)	0.72 (0.65, 0.78)
501.0	Pneumonitis due to inhalation of food or vomitus	respiratory	0.75 (0.70, 0.80)	0.68 (0.62, 0.73)	0.75 (0.69, 0.81)	0.65 (0.56, 0.72)
415.11	Pulmonary embolism and infarction, acute	circulatory system	0.80 (0.75, 0.85)	0.74 (0.68, 0.79)	0.85 (0.78, 0.91)	0.76 (0.68, 0.83)
427.11	Paroxysmal supraventricular tachycardia	circulatory system	0.79 (0.76, 0.81)	0.72 (0.69, 0.76)	0.87 (0.84, 0.90)	0.74 (0.69, 0.78)
415.1	Acute pulmonary heart disease	circulatory system	0.80 (0.75, 0.85)	0.74 (0.68, 0.80)	0.84 (0.77, 0.90)	0.76 (0.68, 0.82)
411.8	Other chronic ischemic heart disease, unspecified	circulatory system	0.81 (0.76, 0.85)	0.75 (0.69, 0.79)	0.81 (0.76, 0.86)	0.73 (0.66, 0.79)
440.22	Atherosclerosis of native arteries of the extremities with intermittent claudication	circulatory system	0.80 (0.73, 0.86)	0.75 (0.68, 0.83)	0.82 (0.74, 0.89)	0.75 (0.67, 0.84)
411.9	Other acute and subacute forms of ischemic heart disease	circulatory system	0.78 (0.72, 0.85)	0.72 (0.64, 0.79)	0.79 (0.71, 0.88)	0.74 (0.65, 0.82)
509.1	Respiratory failure	respiratory	0.77 (0.73, 0.80)	0.70 (0.65, 0.74)	0.76 (0.71, 0.82)	0.69 (0.63, 0.75)
740.2	Osteoarthrosis, generalized	musculoskeletal	0.80 (0.76, 0.83)	0.74 (0.69, 0.79)	0.85 (0.80, 0.89)	0.77 (0.71, 0.83)
427.1	Paroxysmal tachycardia, unspecified	circulatory system	0.78 (0.76, 0.81)	0.73 (0.70, 0.75)	0.87 (0.84, 0.89)	0.74 (0.70, 0.77)
427.4	Cardiac arrest and ventricular fibrillation	circulatory system	0.77 (0.68, 0.84)	0.71 (0.63, 0.78)	0.76 (0.66, 0.86)	0.73 (0.65, 0.81)
569.2	Gastrointestinal complications	digestive	0.76 (0.70, 0.81)	0.69 (0.62, 0.77)	0.79 (0.71, 0.87)	0.68 (0.59, 0.77)
427.12	Paroxysmal ventricular tachycardia	circulatory system	0.78 (0.74, 0.82)	0.72 (0.68, 0.77)	0.84 (0.79, 0.88)	0.71 (0.65, 0.77)
260.2	severe protein-calorie malnutrition	endocrine/metabolic	0.79 (0.74, 0.83)	0.73 (0.68, 0.78)	0.83 (0.75, 0.90)	0.77 (0.70, 0.84)
285.2	Anemia of chronic disease	hematopoietic	0.76 (0.72, 0.79)	0.70 (0.66, 0.75)	0.77 (0.72, 0.82)	0.69 (0.62, 0.75)
459.9	Circulatory disease NEC	circulatory system	0.77 (0.74, 0.79)	0.71 (0.68, 0.74)	0.84 (0.80, 0.87)	0.74 (0.69, 0.78)
276.13	Hyperpotassemia	endocrine/metabolic	0.75 (0.71, 0.80)	0.70 (0.65, 0.75)	0.79 (0.73, 0.85)	0.69 (0.64, 0.75)
574.2	Calculus of bile duct	digestive	0.76 (0.70, 0.80)	0.70 (0.63, 0.78)	0.79 (0.72, 0.85)	0.72 (0.65, 0.79)
250.1	Type 1 diabetes	endocrine/metabolic	0.75 (0.69, 0.81)	0.70 (0.64, 0.75)	0.77 (0.70, 0.82)	0.72 (0.65, 0.78)

Supplementary Table 13. Comparison of performance metrics, including C-Index and AUROC (6 Years) for SleepFM and demographics model. Conditions are ranked by their relative improvement over demographics model, retaining only conditions where the C-Index for SleepFM exceeds 0.75. All metrics include 95% confidence intervals calculated using bootstrapping.

Phecode	Phenotype	Category	C-Index		AUROC (6 Years)	
			SleepFM	End-to-End PSG	SleepFM	End-to-End PSG
440.21	Atherosclerosis of native arteries of the extremities with ulceration or gangrene	circulatory system	0.92 (0.88, 0.95)	0.66 (0.50, 0.89)	0.95 (0.92, 0.98)	0.65 (0.61, 0.69)
264.2	Failure to thrive (childhood)	endocrine/metabolic	0.77 (0.68, 0.88)	0.45 (0.26, 0.67)	0.75 (0.65, 0.87)	0.44 (0.26, 0.66)
358.0	Myoneural disorders	neurological	0.81 (0.73, 0.88)	0.60 (0.48, 0.71)	0.84 (0.75, 0.91)	0.54 (0.40, 0.69)
315.0	Develomental delays and disorders	mental disorders	0.80 (0.77, 0.84)	0.61 (0.54, 0.67)	0.84 (0.79, 0.87)	0.61 (0.52, 0.69)
509.2	Respiratory insufficiency	respiratory	0.79 (0.72, 0.85)	0.62 (0.53, 0.69)	0.82 (0.72, 0.91)	0.64 (0.54, 0.73)
277.4	Disorders of bilirubin excretion	endocrine/metabolic	0.79 (0.70, 0.85)	0.61 (0.47, 0.75)	0.89 (0.84, 0.93)	0.60 (0.41, 0.78)
344.0	Other paralytic syndromes	neurological	0.77 (0.72, 0.83)	0.60 (0.48, 0.69)	0.82 (0.75, 0.88)	0.54 (0.40, 0.68)
276.42	Alkalosis	endocrine/metabolic	0.83 (0.77, 0.88)	0.71 (0.63, 0.78)	0.87 (0.77, 0.93)	0.71 (0.59, 0.81)
250.25	Diabetes type 2 with peripheral circulatory disorders	endocrine/metabolic	0.87 (0.83, 0.91)	0.78 (0.72, 0.84)	0.89 (0.85, 0.93)	0.79 (0.70, 0.87)
504.0	Other alveolar and parietoalveolar pneumonopathy	respiratory	0.85 (0.80, 0.89)	0.75 (0.68, 0.81)	0.90 (0.84, 0.95)	0.76 (0.66, 0.84)
249.0	Secondary diabetes mellitus	endocrine/metabolic	0.79 (0.74, 0.84)	0.67 (0.62, 0.71)	0.82 (0.76, 0.88)	0.66 (0.59, 0.72)
250.24	Type 2 diabetes with neurological manifestations	endocrine/metabolic	0.81 (0.77, 0.85)	0.71 (0.68, 0.75)	0.86 (0.81, 0.90)	0.73 (0.67, 0.78)
280.2	Iron deficiency anemia secondary to blood loss (chronic)	hematopoietic	0.77 (0.73, 0.81)	0.64 (0.60, 0.69)	0.84 (0.79, 0.89)	0.66 (0.60, 0.71)
707.2	Chronic ulcer of leg or foot	dermatologic	0.80 (0.76, 0.85)	0.70 (0.65, 0.75)	0.84 (0.79, 0.88)	0.73 (0.67, 0.79)
276.4	Acid-base balance disorder	endocrine/metabolic	0.77 (0.72, 0.82)	0.67 (0.61, 0.72)	0.79 (0.73, 0.85)	0.67 (0.60, 0.74)
300.9	Posttraumatic stress disorder	mental disorders	0.75 (0.70, 0.79)	0.64 (0.59, 0.69)	0.82 (0.76, 0.87)	0.63 (0.56, 0.69)
315.2	Speech and language disorder	mental disorders	0.81 (0.74, 0.87)	0.73 (0.64, 0.82)	0.83 (0.74, 0.90)	0.71 (0.60, 0.83)
569.2	Gastrointestinal complications	digestive	0.76 (0.70, 0.81)	0.66 (0.59, 0.72)	0.79 (0.71, 0.87)	0.65 (0.57, 0.73)
426.8	Other cardiac conduction disorders	circulatory system	0.79 (0.75, 0.82)	0.71 (0.66, 0.75)	0.82 (0.77, 0.87)	0.69 (0.62, 0.76)
458.2	Iatrogenic hypotension	circulatory system	0.76 (0.68, 0.83)	0.67 (0.60, 0.74)	0.77 (0.65, 0.87)	0.67 (0.56, 0.77)
250.7	Diabetic retinopathy	endocrine/metabolic	0.81 (0.77, 0.85)	0.74 (0.69, 0.79)	0.84 (0.79, 0.89)	0.76 (0.69, 0.82)
276.11	Hyperosmolality and/or hypernatremia	endocrine/metabolic	0.76 (0.66, 0.86)	0.68 (0.59, 0.76)	0.77 (0.59, 0.94)	0.74 (0.62, 0.85)
264.0	Lack of normal physiological development	endocrine/metabolic	0.77 (0.67, 0.86)	0.69 (0.55, 0.81)	0.72 (0.60, 0.84)	0.63 (0.48, 0.77)
440.22	Atherosclerosis of native arteries of the extremities with intermittent claudication	circulatory system	0.80 (0.73, 0.86)	0.74 (0.65, 0.81)	0.82 (0.74, 0.89)	0.74 (0.65, 0.83)
274.11	Gouty arthropathy	endocrine/metabolic	0.76 (0.71, 0.81)	0.68 (0.63, 0.73)	0.77 (0.70, 0.84)	0.67 (0.60, 0.74)
509.1	Respiratory failure	respiratory	0.77 (0.73, 0.80)	0.69 (0.64, 0.73)	0.76 (0.71, 0.82)	0.68 (0.62, 0.74)
260.2	severe protein-calorie malnutrition	endocrine/metabolic	0.79 (0.74, 0.83)	0.71 (0.67, 0.76)	0.83 (0.75, 0.90)	0.72 (0.64, 0.80)
550.4	Umbilical hernia	digestive	0.79 (0.74, 0.83)	0.72 (0.64, 0.79)	0.84 (0.78, 0.90)	0.75 (0.66, 0.83)
513.8	Disorders of diaphragm	respiratory	0.75 (0.65, 0.84)	0.68 (0.57, 0.77)	0.73 (0.58, 0.86)	0.63 (0.48, 0.79)
260.6	Anorexia	endocrine/metabolic	0.76 (0.71, 0.80)	0.68 (0.62, 0.74)	0.84 (0.79, 0.88)	0.68 (0.58, 0.77)
530.2	Esophageal bleeding (varices/hemorrhage)	digestive	0.76 (0.72, 0.79)	0.69 (0.64, 0.74)	0.87 (0.84, 0.90)	0.69 (0.61, 0.75)
440.2	Atherosclerosis of the extremities	circulatory system	0.81 (0.74, 0.86)	0.75 (0.69, 0.81)	0.84 (0.75, 0.90)	0.78 (0.71, 0.85)
411.9	Other acute and subacute forms of ischemic heart disease	circulatory system	0.78 (0.72, 0.85)	0.72 (0.66, 0.79)	0.79 (0.71, 0.88)	0.75 (0.68, 0.82)
227.0	Benign neoplasm of other endocrine glands and related structures	neoplasms	0.75 (0.66, 0.84)	0.69 (0.59, 0.77)	0.82 (0.69, 0.93)	0.72 (0.59, 0.83)
250.1	Type 1 diabetes	endocrine/metabolic	0.75 (0.69, 0.81)	0.68 (0.62, 0.75)	0.77 (0.70, 0.82)	0.70 (0.64, 0.78)
285.1	Acute posthemorrhagic anemia	hematopoietic	0.78 (0.75, 0.81)	0.72 (0.68, 0.75)	0.83 (0.79, 0.87)	0.73 (0.68, 0.78)
415.1	Acute pulmonary heart disease	circulatory system	0.80 (0.75, 0.85)	0.75 (0.69, 0.81)	0.84 (0.77, 0.90)	0.76 (0.69, 0.83)
348.8	Encephalopathy, not elsewhere classified	neurological	0.77 (0.70, 0.83)	0.71 (0.64, 0.77)	0.78 (0.70, 0.86)	0.70 (0.62, 0.78)
427.11	Paroxysmal supraventricular tachycardia	circulatory system	0.79 (0.76, 0.81)	0.73 (0.70, 0.76)	0.87 (0.84, 0.90)	0.75 (0.70, 0.79)
427.12	Paroxysmal ventricular tachycardia	circulatory system	0.78 (0.74, 0.82)	0.72 (0.67, 0.77)	0.84 (0.79, 0.88)	0.72 (0.65, 0.78)
428.2	Heart failure NOS	circulatory system	0.77 (0.70, 0.84)	0.71 (0.64, 0.78)	0.81 (0.74, 0.88)	0.72 (0.64, 0.80)
427.1	Paroxysmal tachycardia, unspecified	circulatory system	0.78 (0.76, 0.81)	0.73 (0.71, 0.76)	0.87 (0.84, 0.89)	0.75 (0.71, 0.79)
501.0	Pneumonitis due to inhalation of food or vomitus	respiratory	0.75 (0.70, 0.80)	0.69 (0.62, 0.76)	0.75 (0.69, 0.81)	0.66 (0.57, 0.74)
276.13	Hyperpotassemia	endocrine/metabolic	0.75 (0.71, 0.80)	0.70 (0.65, 0.74)	0.79 (0.73, 0.85)	0.69 (0.63, 0.74)
459.9	Circulatory disease NEC	circulatory system	0.77 (0.74, 0.79)	0.71 (0.68, 0.75)	0.84 (0.80, 0.87)	0.74 (0.69, 0.78)
574.2	Calculus of bile duct	digestive	0.76 (0.70, 0.80)	0.70 (0.63, 0.78)	0.79 (0.72, 0.85)	0.72 (0.64, 0.80)

Supplementary Table 14. Comparison of performance metrics, including C-Index and AUROC (6 Years) for SleepFM and End-to-End PSG model. Conditions are ranked by their relative improvement over End-to-End PSG model, retaining only conditions where the C-Index for SleepFM exceeds 0.75. All metrics include 95% confidence intervals calculated using bootstrapping.

Modality	Channel	Prevalence (%)
RESP	Airflow	100.00
RESP	HR	99.92
RESP	Abdominal	73.92
RESP	SaO2	73.92
RESP	Thoracic	73.92
RESP	ABD	26.08
RESP	Chest	26.08
RESP	SpO2	26.08
BAS	C3	99.85
BAS	C4	99.85
BAS	LOC	73.92
BAS	ROC	73.92
BAS	A1	73.74
BAS	A2	73.74
BAS	M1	26.11
BAS	M2	26.11
BAS	E1	26.08
BAS	E2	26.08
EKG	ECG L	73.77
EKG	ECG R	73.77
EKG	ECGL	26.08
EKG	ECGR	26.08
EKG	ECG L-ECG R	0.15
EMG	Leg L	73.92
EMG	Leg R	73.92
EMG	L Chin	73.77
EMG	R Chin	73.77
EMG	LChin	26.08
EMG	LegL	26.08
EMG	LegR	26.08
EMG	RChin	26.08
EMG	L Chin-R Chin	0.15

Supplementary Table 15. Channels and prevalence percentages by modality for MROS.

Modality	Channel	Prevalence (%)
RESP	Abdo	100.00
RESP	HR	100.00
RESP	Snore	100.00
RESP	SpO2	100.00
RESP	Thor	100.00
RESP	Therm	99.37
BAS	EEG1	100.00
BAS	EEG2	100.00
BAS	EEG3	100.00
BAS	EOG-L	100.00
BAS	EOG-R	100.00
EKG	EKG	100.00
EMG	EMG	100.00
EMG	Leg	100.00
EMG	Pleth	100.00

Supplementary Table 16. Channels and prevalence percentages by modality for MESA.

Modality	Channel	Prevalence (%)
RESP	ABDO RES	100.00
RESP	SaO2	100.00
RESP	THOR RES	100.00
RESP	AIRFLOW	75.94
RESP	H.R.	68.69
RESP	NEW AIR	46.64
RESP	New Air	9.43
RESP	NEWAIR	4.14
RESP	AIRFLOW-0	1.08
RESP	AIRFLOW-1	1.08
EKG	ECG	100.00
BAS	EEG	100.00
BAS	EOG(L)	100.00
BAS	EOG(R)	100.00
BAS	EEG(sec)	97.95
BAS	EEG 2	1.15
BAS	EEG2	0.65
BAS	EEG sec	0.15
BAS	EEG(SEC)	0.09
EMG	EMG	100.00
EMG	LEG(L)	0.02
EMG	LEG(R)	0.02

Supplementary Table 17. Channels and prevalence percentages by modality for SHHS.

Modality	Channel	Prevalence (%)
BAS	LOC	70.10
BAS	ROC	70.10
BAS	A1A2	68.81
BAS	C3M2	60.02
BAS	C4M1	60.02
BAS	O1M2	60.02
BAS	O2M1	60.02
BAS	F3M2	58.78
BAS	F4M1	58.78
BAS	C3A2	36.61
RESP	Abd	83.75
RESP	SpO2	72.65
RESP	Snore	70.40
RESP	Pulse	27.17
RESP	SAO2	26.81
RESP	SNOR	16.34
RESP	PPG	13.07
RESP	ABD	12.29
RESP	Abdomen	2.03
RESP	Thorax	2.03
EMG	LLeg	75.01
EMG	RLeg	75.01
EMG	Chin	67.73
EMG	CHIN	26.86
EMG	LLEG	18.28
EMG	RLEG	18.28
EMG	InterEMG	2.91
EMG	Pleth	2.42
EMG	CHINEMG	1.96
EMG	LLEGEMG	1.96
EKG	ECG	92.22
EKG	EKG	2.35
EKG	ECGI-0	1.83
EKG	ECGI-1	1.83
EKG	ECGII-0	1.83
EKG	ECGII-1	1.83
EKG	ECG1	1.27
EKG	ECGI	1.23
EKG	ECG2	0.41
EKG	ECG II	0.23

Supplementary Table 18. Channels and prevalence percentages by modality for BioSerenity.

Modality	Channel	Prevalence (%)
RESP	Snore	88.04
RESP	Chest	83.96
RESP	SpO2	66.98
RESP	Abd	65.96
RESP	Pulse Rate	45.14
RESP	Nasal	43.81
RESP	Nasal Pressure	43.49
RESP	Oral Therm	42.88
RESP	Pulse	39.97
RESP	Oral-CO2	37.94
BAS	E1-Cz	43.53
BAS	C3-Cz	43.31
BAS	C4-Cz	43.31
BAS	M1-Cz	43.31
BAS	M2-Cz	43.31
BAS	O1-Cz	43.31
BAS	C3	43.11
BAS	C4	43.11
BAS	O1	43.11
BAS	O2	43.11
EMG	Chin	31.40
EMG	Chin2	28.35
EMG	Chin EMG	27.09
EMG	Chin_Ctr-Cz	26.01
EMG	Chin_L-Cz	26.01
EMG	Chin_R-Cz	26.01
EMG	Feet-R	19.18
EMG	Pleth	17.60
EMG	Arms-1	17.52
EMG	Arms-2	17.52
EKG	EKG_L-Cz	43.31
EKG	EKG_R-Cz	43.31
EKG	ECG	17.78
EKG	EKG-L	12.01
EKG	EKG-R	12.01
EKG	ECG II	11.87
EKG	EKG	9.07
EKG	ECG 2	1.48
EKG	EKG2	0.02
EKG	ECG1-EC	0.00

Supplementary Table 19. Channels and prevalence percentages by modality for SSC.
