# Supplementary material for: A multimodal sleep foundation model for disease prediction
Source: Nat Med. 2026 Jan 6;32(2):752–62. doi: 10.1038/s41591-025-04133-4 (PMC12920147; doi:10.1038/s41591-025-04133-4)
Supplement: Supplementary file 2 — Reporting Summary [file 41591_2025_4133_MOESM2_ESM.pdf]

nature portfolio

Corresponding author(s): James zou

Last updated by author(s): 10/14/2025

Reporting Summary

Nature Portfolio wishes to improve the reproducibility of the work that we publish. This form provides structure for consistency and transparency in reporting. For further information on Nature Portfolio policies, see our Editorial Policies and the Editorial Policy Checklist.

Statistics

For all statistical analyses, confirm that the following items are present in the figure legend, table legend, main text, or Methods section.

n/a	Confirmed	
☐	☒	The exact sample size (*n*) for each experimental group/condition, given as a discrete number and unit of measurement
☐	☒	A statement on whether measurements were taken from distinct samples or whether the same sample was measured repeatedly
☐	☒	The statistical test(s) used AND whether they are one- or two-sided<br>*Only common tests should be described solely by name; describe more complex techniques in the Methods section.*
☐	☒	A description of all covariates tested
☐	☒	A description of any assumptions or corrections, such as tests of normality and adjustment for multiple comparisons
☐	☒	A full description of the statistical parameters including central tendency (e.g. means) or other basic estimates (e.g. regression coefficient) AND variation (e.g. standard deviation) or associated estimates of uncertainty (e.g. confidence intervals)
☐	☒	For null hypothesis testing, the test statistic (e.g. *F*, *t*, *r*) with confidence intervals, effect sizes, degrees of freedom and *P* value noted<br>*Give P values as exact values whenever suitable.*
☒	☐	For Bayesian analysis, information on the choice of priors and Markov chain Monte Carlo settings
☒	☐	For hierarchical and complex designs, identification of the appropriate level for tests and full reporting of outcomes
☒	☐	Estimates of effect sizes (e.g. Cohen's *d*, Pearson's *r*), indicating how they were calculated

Our web collection on statistics for biologists contains articles on many of the points above.

Software and code

Policy information about availability of computer code

Data collection	The PSG data was gathered in a hippa compliant and secure compute cluster in Google Cloud Platform. Patient electronic health record data was also stored in the secure compute cluster and used exclusively within the cluster.
Data analysis	All data analysis and processing were performed using Python (version 3.10.14) and its data analysis libraries, including Pandas (2.1.1), NumPy (1.25.2), SciPy (1.11.3), scikit-survival (0.23.0), scikit-learn (1.5.2), and PyTorch (2.0.1). Our codebase is available at https://github.com/zou-group/sleepfm-clinical.

For manuscripts utilizing custom algorithms or software that are central to the research but not yet described in published literature, software must be made available to editors and reviewers. We strongly encourage code deposition in a community repository (e.g. GitHub). See the Nature Portfolio guidelines for submitting code & software for further information.

Data

Policy information about availability of data

All manuscripts must include a data availability statement. This statement should provide the following information, where applicable:

- Accession codes, unique identifiers, or web links for publicly available datasets
- A description of any restrictions on data availability
- For clinical datasets or third party data, please ensure that the statement adheres to our policy

Of the five data sources used in this study, three datasets are publicly available and can be accessed at the following links: SHHS (https://archive.physionet.org/physiobank/database/shhpsgdb), MrOS (https://sleepdata.org/datasets/mros), and MESA (https://sleepdata.org/datasets/mesa). The Bioserenity dataset is proprietary and has been shared with Stanford for research and development purposes. We will also release the SSC PSG data upon paper publication. Our codebase is includes in (https://github.com/zou-group/sleepfm-clinical)

Research involving human participants, their data, or biological material

Policy information about studies with human participants or human data. See also policy information about sex, gender (identity/presentation), and sexual orientation and race, ethnicity and racism.

Reporting on sex and gender	Gender information was collected from patient electronic health records and included as a feature in both the main and baseline models to evaluate performance. We also report gender statistics in the demographics table. Informed consent was obtained for the use of electronic health record data, and no individual-level data is reported in this paper.
Reporting on race, ethnicity, or other socially relevant groupings	Race and ethnicity information was obtained from the electronic health record system and was entirely self-reported. Informed consent was obtained for its use. This information was not used as a proxy for other determinants such as socio-economic status but was included in the demographics model as a baseline for comparison with the main model. Summary statistics for race and ethnicity are provided in the demographics table in the paper.
Population characteristics	Age information was directly extracted from participants' electronic health records (EHR). Past and current patient diagnosis data were also obtained from EHR. This information was used to build the main model and define the outcome of interest for disease prediction. Age statistics are presented in the demographics table in the main paper, while summary statistics on disease prevalence are provided in the supplementary section.
Recruitment	The publicly available datasets were downloaded from the internet following appropriate protocols. The SSC dataset was collected from the Stanford Sleep Clinic as part of a regular clinical sleep study. The BioSerenity dataset was also obtained from a sleep study conducted by the private company BioSerenity. Consent was obtained for the use of these datasets for research p
Ethics oversight	Ethical approval for this study was obtained from the Stanford University Institutional Review Board (IRB), protocol number: 69873, and consent was obtained for the use of these datasets for research purposes.

Note that full information on the approval of the study protocol must also be provided in the manuscript.

Field-specific reporting

Please select the one below that is the best fit for your research. If you are not sure, read the appropriate sections before making your selection.

☒ Life sciences ☐ Behavioural & social sciences ☐ Ecological, evolutionary & environmental sciences

For a reference copy of the document with all sections, see nature.com/documents/nr-reporting-summary-flat.pdf

Life sciences study design

All studies must disclose on these points even when the disclosure is negative.

Sample size	The sample size was determined by the datasets available to us, including publicly available datasets and our two internal datasets from Stanford and BioSerenity.
Data exclusions	Some datasets were filtered out due to corrupted files or missing the necessary minimum information.
Replication	All results reported in this paper are reproducible, given data access and code availability. The code is publicly released, and the dataset from the Stanford cohort will be made available in the future. Performance on publicly available datasets should also be reproducible.
Randomization	Participants were randomly assigned to different training and testing splits that comprised our analysis.

Blinding	The random allocation of data to training and testing splits was performed after all data had been collected, ensuring no prior knowledge of the splits.

Reporting for specific materials, systems and methods

We require information from authors about some types of materials, experimental systems and methods used in many studies. Here, indicate whether each material, system or method listed is relevant to your study. If you are not sure if a list item applies to your research, read the appropriate section before selecting a response.

Materials & experimental systems

n/a	Involved in the study	
☒	☐	Antibodies
☒	☐	Eukaryotic cell lines
☒	☐	Palaeontology and archaeology
☒	☐	Animals and other organisms
☐	☒	Clinical data
☒	☐	Dual use research of concern
☒	☐	Plants

Methods

n/a	Involved in the study	
☒	☐	ChIP-seq
☒	☐	Flow cytometry
☒	☐	MRI-based neuroimaging

Clinical data

Policy information about clinical studies

All manuscripts should comply with the ICMJE guidelines for publication of clinical research and a completed CONSORT checklist must be included with all submissions.

Clinical trial registration	This study does not involve a clinical trial and is not subject to clinical trial registration requirements. The dataset consists of retrospective electronic health record (EHR) and polysomnography (PSG) data, collected as part of routine clinical practice. Ethical approval was obtained from the Stanford University Institutional Review Board (IRB), protocol number: 69873, and informed consent was obtained where applicable. Data collection and outcome measures are described in the Methods section.
Study protocol	Ethical approval was obtained from the Stanford University Institutional Review Board (IRB), protocol number: 69873
Data collection	N/A
Outcomes	N/A

Plants

Seed stocks	N/A
Novel plant genotypes	N/A
Authentication	N/A
